# Aberrant R-loop–mediated immune evasion, cellular communication, and metabolic reprogramming affect cancer progression: a single-cell analysis

**DOI:** 10.1186/s12943-023-01924-6

**Published:** 2024-01-10

**Authors:** Shichao Zhang, Yang Liu, Yichi Sun, Qin Liu, Yan Gu, Ya Huang, Zhu Zeng, Fuzhou Tang, Yan Ouyang

**Affiliations:** 1 Key Laboratory of Infectious Immune and Antibody Engineering of Guizhou Province, Engineering Research Center of Cellular Immunotherapy of Guizhou Province, Guizhou Medical University, Guiyang, China; 2 Immune Cells and Antibody Engineering Research Center of Guizhou Province, Key Laboratory of Biology and Medical Engineering, Guizhou Medical University, Guiyang, China

**Keywords:** R-loop, Tumor immune escape phenotype, Cell communication, Immunotherapy, FANCI, Non-small cell lung cancer

## Abstract

Dysregulation of R-loop homeostasis is closely related to various human diseases, including cancer. However, the causality of aberrant R-loops in tumor progression remains unclear. In this study, using single-cell RNA-sequencing datasets from lung adenocarcinoma (LUAD), we constructed an R-loop scoring model to characterize the R-loop state according to the identified R-loop regulators related to EGFR mutations, tissue origins, and TNM stage. We then evaluated the relationships of the R-loop score with the tumor microenvironment (TME) and treatment response. Furthermore, the potential roles of FANCI-mediated R-loops in LUAD were explored using a series of in *vitro* experiments. Results showed that malignant cells with low R-loop scores displayed glycolysis and epithelial–mesenchymal transition pathway activation and immune escape promotion, thereby hampering the antitumor therapeutic effects. Cell communication analysis suggested that low R-loop scores contributed to T cell exhaustion. We subsequently validated the prognostic value of R-loop scores by using bulk transcriptome datasets across 33 tumor types. The R-loop scoring model well predicted patients’ therapeutic response to targeted therapy, chemotherapy, or immunotherapy in 32 independent cohorts. Remarkably, changes in R-loop distribution mediated by FANCI deficiency blocked the activity of Ras signaling pathway, suppressing tumor-cell proliferation and dissemination. In conclusion, this study reveals the underlying molecular mechanism of metabolic reprogramming and T cell exhaustion under R-loop score patterns, and the changes in R-loops mediated by R-loop regulators resulting in tumor progression. Therefore, incorporating anticancer methods based on R-loop or R-loop regulators into the treatment schemes of precision medicine may be beneficial.

**Supplementary Information:**

The online version contains supplementary material available at 10.1186/s12943-023-01924-6.

## Introduction

R-loops are dynamic triple-stranded nucleic acid structures that form during transcription when a nascent guanine-rich RNA transcript segment invades the DNA template [1]. R-loops, which are ubiquitous in mammalian genomes, participate in multiple biological processes, leading to both positive and negative outcomes. Physiological R-loops contribute to DNA repair, cell proliferation and differentiation, and RNA transcription and processing, as well as regulating gene expression and DNA methylation [2, 3]. However, changes in the R-loop homeostasis disrupt R-loop-regulated processes, causing replication stress and genomic instability; hence, such changes may be associated with various human diseases, including cancer [4–6]. Thus, the R-loop must be regulated finely. Currently, over 1,000 R-loop regulators that control R-loop structures and levels have been reported [7]. Bhatia et al., found that the loss of BRCA2, an R-loop regulator, results in unscheduled accumulation of R-loops, suggesting that R-loops are responsible for triggering chromosome abnormalities in BRCA2-deficient cells [8]. Wang et al. suggested that ZFP281 has a critical role in inhibiting R-loop formation and maintaining DNA replication [9]. Robbiani et al., showed that RNase H overexpression degrades R-loops, thereby suppressing genomic instability related to CFS [10]. The findings of the functional roles of R-loops in health and disease may contribute to the development of new targeted treatment strategies for human diseases.

Given that R-loops contribute to genomic instability and replication stress, which are the hallmarks of pre-neoplastic and neoplastic cells, they are viewed as potential cancer drivers [11]. Numerous studies have correlated the alterations of R-loop frequency, stability, or genomic position with cancerous states such as proto-oncogene activation and tumor suppressor gene inactivation. For instance, an R-loop produced through translocations between MYC proto-oncogene and Ig S region promotes tumorigenic process [12]. R-loop dissolution driven by inositol auxotroph 80 facilitates the proliferation of multiple cancer cell types, including prostate, breast, and melanoma cancer cells [13, 14]. R-loop accumulation induced by topoisomerase III beta gene deletion predisposes individuals to renal cancer [15]. Furthermore, abnormal R-loops generated by the loss of BRE1, a tumor suppressor gene, are related to carcinogenesis [16]. Methyltransferase-like 8-mediated 3-methylcytidine modification in RNA also modulates R-loops to facilitate carcinogenesis [17]. However, direct evidence that R-loops themselves lead to tumorigenesis and tumor progression is limited.

Cancer cells exist in a tumor microenvironment (TME) composed of stromal cells such as fibroblasts, endothelial cells, epithelial cells, and myeloid cells, and TME regulates both tumor progression and tumor response to therapy [18]. R-loop accumulation in the cytoplasm has been reported to activate an innate immune response that contributes to diseases such as neurodegeneration and cancer [19]. During chronic inflammation and carcinogenesis, R-loops regulate cytokine-mediated inflammatory cascades by inducing the reorganization of immune cell interactions [20]. Ten-eleven translocation is a methylcytosine dioxygenase that is responsible for the optimal differentiation and function of inducible regulatory T cells, and it also maintains the steady state of mature B cells by degrading accumulated R-loops [21, 22]. Nevertheless, function of R-loops in TME remains unclear. Therefore, a single-cell analysis should be conducted to provide insights into the roles of R-loops within and between malignant and nonmalignant cells inside the tumor tissue at a single-cell resolution.

Using single-cell RNA-sequencing (RNA-seq) datasets from lung adenocarcinoma (LUAD), this study constructed an R-loop scoring model to characterize the R-loop state based on the identified R-loop regulators related to EGFR mutations, tissue origins, and TNM stage. Our results imply that R-loop scoring can characterize TME features and predict the antitumor treatment effects. Low R-loop scores were linked to unfavorable outcomes, resistance to anticancer therapies (e.g., targeted therapy and chemotherapy), and nonresponse to immunotherapy. Importantly, we revealed the causal role of changes in R-loop distribution mediated by an R-loop regulator for LUAD development. Altogether, our study provided a theoretical basis for targeting R-loops or R-loop regulators to improve clinical responses to cancer therapy.

## Material and methods

### Data acquisition and processing

Three single-cell RNA-seq (scRNA-seq) datasets of patients with LUAD and associated clinical information were collected from the Gene Expression Omnibus (GEO) database (http://www.ncbi.nlm.nih.gov/geo/: GSE123904, GSE131907, and GSE146100). Moreover, an independent dataset containing the data of LUAD samples that underwent tyrosine kinase inhibitor (TKI) therapy was collected from the National Center for Biotechnology Information (NCBI) BioProject (PRJNA591860).

Based on a previous reported method [23], we obtained four lung cancer scRNA-seq datasets (GSE123904, GSE131907, PRJNA591860, and GSE146100). The dataset selection procedure in the PubMed was summarized as follows: (((((lung cancer[Title]) OR (lung adenocarcinomas[Title])) OR (NSCLC[Title/Abstract])) OR (lung tumor[Title/Abstract])) OR (neoadjuvant immunotherapy[Title])) AND (((single-cell transcript*[Title/Abstract]) OR (single-cell RNA[Title/Abstract])) OR (scRNA-seq[Title/Abstract])) AND (y_5[Filter]). The results were shown in Figure S1. Finally, four scRNA-seq datasets from published studies of LUAD patients based on the above criterion were identified and further analyzed in followed study (Table S1).

Lung cancer scRNA-seq data were processed using the scRNA-seq package ‘Seurat’ in R. We first created a Seurat object using CreateSeuratObject function in ‘Seurat’. The raw data were filtered by removing cells expressing less than 300 genes and genes that were expressed in fewer than three cells, and by removing cells with the total mitochondrial gene expression more than 8%. Counts were normalized by the LogNormalize method and were scaled using the ScaleData function for downstream analysis. We identified the highly variable genes based on the the Seurat function FindVariableFeatures with a cut-off value (2000). We integrated the GSE123904 and GSE131907 datasets. The batch effect was eliminated by RunHarmony function in ‘Harmony’ R package.

To evaluate the clinical performances of the R-loop score according to the independent bulk data, we collected samples with RNA-seq expression profiles and complete clinical information from The Cancer Genome Atlas (TCGA, https://www.cancer. gov/tcga) database across the following 33 tumor types: thyroid carcinoma (THCA), prostate adenocarcinoma (PRAD), pancreatic adenocarcinoma (PAAD), adrenocortical carcinoma, bladder urothelial carcinoma (BLCA), breast cancer (BRCA), cervical squamous cell carcinoma and endocervical adenocarcinoma, cholangiocarcinoma (CHOL), colon adenocarcinoma, lymphoid neoplasm diffuse large B-cell lymphoma (DLBC), esophageal carcinoma, glioblastoma multiforme (GBM), head and neck squamous carcinoma (HNSC), kidney chromophobe, kidney renal clear cell carcinoma, acute myeloid leukemia, kidney renal papillary cell carcinoma, brain low-grade glioma, liver hepatocellular carcinoma (LIHC), lung squamous cell carcinoma, mesothelioma, ovarian serous cystadenocarcinoma (OV), pheochromocytoma and paraganglioma, rectal adenocarcinoma, sarcoma, skin cutaneous melanoma (SKCM), stomach adenocarcinoma, testicular germ cell tumor, thymoma, uterine corpus endometrial carcinoma, uterine carcinosarcoma, uveal melanoma, and LUAD. Furthermore, 38 published high-throughput microarray datasets were obtained from GEO and NCBI BioProject databases for subsequent analysis. The fragments per kilobase million (FPKM) values from RNA-seq data were acquired. For each gene, RNA-Seq data read counts were normalized as FPKM using log2-transformation. The code used in analysis for this study is provided in Supplementary Code.

### Weighted gene coexpression network analysis (WGCNA) of single-cell data for identifying gene network modules

To determine groups of cells sharing similar origin, we conducted WGCNA applying the functions in the ‘WGCNA’ R package [24]. We used R-loop regulators for building a signed network and selected the smallest beta value that satisfies the scale-free topology criterion (optimal beta = 14). We set minModuleSize = 10 as a parameter for the dynamic tree-cut function. Highly similar modules were determined through clustering and were further merged, with a height cutoff value of 0.25. According to the function “Eigengenes,” we found that coexpressed gene modules were related to several clinical characteristics; the modules that were significantly associated with mutation, stage, origin, and smoking were chosen.

### Construction of an R-loop scoring model based on R-loop regulators

According to a previously reported algorithm [23], R-loop score was calculated based on the cal_CRDscore function from R package ‘CRDscore’. Briefly, we first calculated the average expression levels of genes in all cells or samples. Subsequently, a random R-loop score (S_ramdom_) was obtained using a random sampling strategy. Next, we analyzed a score for the centered expression data of each sample or cell (S_center_). Finally, we subtracted S_ramdom_ with S_center_ to obtain the R-loop score. In the bulk samples, R-loop score was calculated based on the expression profiles of prognosis-associated differentially expressed R-loop regulators. Cells or samples were divided into high- and low-score subgroups in terms of the median value of R-loop scores.

### Analysis of cell–cell interaction and cytokine signaling activity

Clustering analysis was conducted according to the integrated joint embedding generated by Harmony with the Louvain algorithm. Cell clustering results were then visualized through uniform manifold approximation and projection (UMAP) or t-Distributed stochastic neighbor embedding (t-SNE) analysis. The cell populations were annotated as known cell types based on the expression of cells’ typical marker genes. Moreover, cell communication was analyzed using CellphoneDB [25]. According to the annotated ligand-receptor pairs gained from the STRING database [26], their average expression levels were calculated. Subsequently, we collected the ligand-receptor pairs with significant (*p* < 0.05) values returned by CellphoneDB. Finally, we used these identified pairs to analyze the interaction between two cell types. Considering the critical role of cytokines in cell communication, the activity of cytokine signaling from transcriptomic profiles was determined using CytoSig [27].

### Gene set variation analysis (GSVA)

The difference in the signal pathways involving genes between the high and low R-loop score subgroups was investigated by extracting the Kyoto Encyclopedia of Genes and Genomes (KEGG) pathways and conducting GSVA enrichment analysis using these pathways [28]. An adjusted *p* value less than 0.05 suggests statistically significant differences.

### Cell lines and cell culture

Human non-small-cell lung cancer (NSCLC) cell lines PC-9 and A549 were purchased from National Collection of Authenticated Cell Cultures. Cells were cultivated in glucose-containing Roswell Park Memorial Institute 1640 (RPMI-1640) medium (1185093, Gibco, Waltham, USA) with 10% fetal bovine serum (C04001, Biological, Industries, Beit, HaEmek, Israel) in a humidified incubator at 37 °C with 5% CO_2_. To generate stable FANCI-knockdown cells, we transiently transfected PC-9 and A549 with lentiviral vectors harboring the shRNA vector GV493 (hU6-MCS-CBh-gcGFP-IRES-puromycin) acquired from GeneChem (Shanghai, China). The shRNA sequences were as follows: sh-con, 5′-TTCTCCGAACGTGTCACGT -3′; shRNA-1, 5′-ATGTAAGCTCGGAGCTAATAT-3′; shRNA-2, 5′-CTAGTTCCTCATAGATCTTAT-3′.

### Gene expression analysis

Gene mRNA levels were quantified using a quantitative real-time PCR (Q-PCR) method. The total RNA was extracted using an RNA extraction reagent (9108, Takara, Osaka, Japan). The 2X Super SYBR Green qPCR Master Mix (OP220, ES Science, Guangzhou, China) was used to analyze mRNA levels. The primer sequences for FANCI were: forward, 5′-CCACCTTTGGTCTATCAGCTTC-3′; reverse, 5′-CAACATCCAATAGCTCGTCACC-3′. The primer sequences for GAPDH were: forward, 5′-GGAGCGAGATCCCTCCAAAAT-3′; reverse, 5′-GGCTGTTGTCATACTTCTCATGG-3′.

In addition, protein expression levels were determined by western blot analysis. Proteins from the collected cells were resolved by 12% sodium dodecyl sulfate–polyacrylamide gel electrophoresis and then transferred onto polyvinylidene fluoride membranes (IPVH00010, Sigma, St. Louis, USA). These membranes were blocked with 5% nonfat dry milk for 2 h at room temperature, followed by overnight incubation with the first antibody, such as rabbit anti-FANCI (1:1,000, 20,789–1-AP, proteintech, WuHan, China), rabbit anti-NFκB-p65 (1:1,000, YT3108, ImmounWay Biotechnology, Plano, TX, USA), rabbit anti-Phospho-PI3-kinase p85/p55 (Y467/199) (1:1,000, YP0224, ImmounWay, Biotechnology, Plano, TX, USA), rabbit anti-phospho-IKKα/β (Ser176/177) (1:1,000,YP0141, ImmounWay Biotechnology, Plano, TX, USA), rabbit anti-Phospho-Akt (S473) (1:1,000, YP0006, ImmounWay Biotechnology, Plano, TX, USA), rabbit anti-phospho-MEK-1/2 (Ser218/222) (1:1,000, YP0167, ImmounWay Biotechnology, Plano, TX, USA), rabbit anti-phospho-ERK1/2 (Tyr204) (1:1,000, YP0101, ImmounWay Biotechnology, Plano, TX, USA), rabbit anti-RasGRF1(1:1,000, YT4009, ImmounWay Biotechnology, Plano, TX, USA), rabbit anti-phospho-Raf-1(S259) (1:1,000, YP0239, ImmounWay Biotechnology, Plano, TX, USA), and rabbit anti-GAPDH (1:10,000, ab181602, Abcam, Cambridge, UK) at 4 °C. After washing, the membranes were further incubated for 1 h using the corresponding secondary antibody conjugated with horseradish peroxidase goat anti-rabbit IgG antibody (1:10,000, abs20040ss, Absin, Shanghai, China).

### Cell functional experiments

For proliferation assays, cells (3,000 per well) were grown in 96-well plates containing 100 μL of complete media at 37 °C. At selected time points (0, 24, 48, 72, and 96 h), 10 μL of Cell Counting Kit-8 (BS350B, Biosharp, Beijing, China) reagent was added, followed by incubation for 2 h. The optical density was read at 450 nm. For scratch assays, we starved confluent cells for 24 h and created a linear wound by using a 10 µL pipette tip. After cell scratching, we collected the images at 24 and 48 h. With regard to transwell migration, cells (5 × 10^4^) were plated into the upper chamber for 24 h. For invasion tests, cells were suspended in a serum-free medium and inoculated into a transwell plate precoated with Matrigel. After 24 h, cells in the bottom chambers were fixed with 4% paraformaldehyde and stained with 0.1% crystal violet. According to the manufacturer’s protocol, apoptosis cells were investigated using the Annexin V-PE/7-ADD Apoptosis Kit (MA049-1, Meilunbio, Dalian, China). For clone formation assays, cells were cultured for 14 days in a complete medium. Cell colonies were fixed with 4% paraformaldehyde for 15 min and further stained with 0.1% crystal violet for 10 min. For tumorigenicity assays, four-week-old nude mice (BALB/c-nude) were randomly distributed into three groups (5 mice each) according to the type of cells injected subcutaneously, namely, the stable sh-con and FANCI-depleted cells such as sh-FANCI-1 and sh-FANCI-2 cells. Every other week, we measured mice’s weight and tumor volume. After 5 weeks, the mice were euthanized, and the tumor volume and tumor weight were further measured.

### DNA/RNA immunoprecipitation and sequencing (DRIP-seq) analysis

DRIP-seq was performed by Aksomics (ShangHai, China) according to a previously reported method [29]. For DRIP-seq analysis, the DNA chain containing the R-loop structure was enriched in *vitro* using an S9.6 antibody that can specifically recognize the DNA:RNA hybrids. Adequate amount of DNA (10 μg) was used and sequencing depth of 6G were carried out. The NGS data were repeated twice. The peak calling regions signal boundaries were determined based on the window size (300 bp), signal-to-noise ratio (5 ~ 50), and *p-*value (< 0.001). The distribution of the R-loops was then determined by high-throughput sequencing.

### Statistical analysis

The differences of continuous variables between two groups were assessed by Student’s *t*-test (parametric) using GraphPad Prism 8 software or Wilcoxon rank sum test (nonparametric) using R software package. Experiments were repeated thrice, and all results are shown as mean ± standard deviation. For all analyses, a *p*-value less than 0.05 indicated a significant difference.

## Results

### R-loop score for malignant cells correlates with the disease outcome in LUAD

**Fig. 1:**
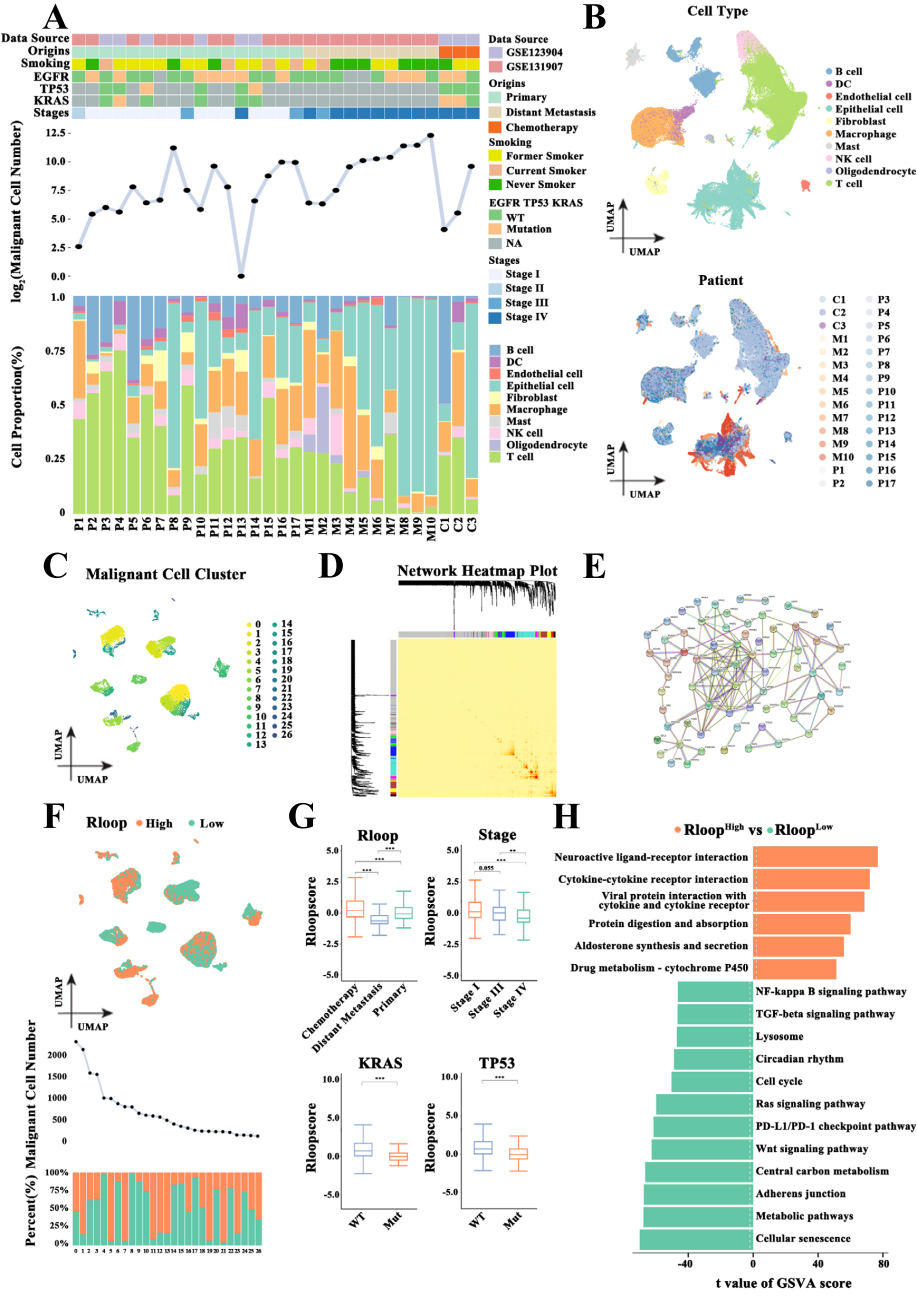
R-loop score from malignant cells related to lung adenocarcinoma (LUAD) progression. **A** Clinical and molecular characteristics of the 30 LUAD samples obtained from GEO datasets (GSE123904 and GSE131907), and the proportion of cell types in each sample. **B** Uniform manifold approximation and projection (UMAP) plot of 92,842 cells colored according to cell types or individual patients. **C** UMAP plot of 27 identified subgroups from malignant cells. **D** Weighted gene coexpression network analysis (WGCNA) of 1,185 R-loop regulators. A deeper color indicates a stronger interaction. **E** Interaction plots of 92 R-loop regulators from purple, brown, magenta, and pink modules. **F** Malignant cells are divided into high- and low-score groups according to the median value of the R-loop scores. The line graph shows the number of malignant cells in each subgroup, and malignant cells with high scores are in orange. **G** The differences in the R-loop score for malignant cells in various conditions (Wilcoxon rank test). **H** Gene set variation analysis (GSVA) showing the differences in the enrichment pathways between malignant cells with high R-loop score and those with low R-loop scores (Bayesian *t*-test). (*p < .05, **p < .01, ***p < .001)

The workflow of our study is shown in Figure S2. To explore the relationship between R-loops and LUAD progression, we first integrated two separate single-cell RNA-seq datasets (GSE123904 and GSE131907). As shown in Fig. 1A, 30 cancer samples were included in the combined dataset, among which three underwent neoadjuvant chemotherapy. Furthermore, 17 samples displayed primary tumor characteristics, and 10 had distant metastatic tumors (untreated patients during surgical resection or endobronchial ultrasound/bronchoscopy). In these samples, 12 contained EGFR mutations (oncogenic drivers) across all tumor development stages.

Using a quality control method, we extracted 26,248 RNA transcripts from 92,842 cells. The proportion of cells in each patient was shown in Figure S3A. Subsequently, 57 cell clusters were obtained through principal component analysis (PCA) followed by the UMAP method (Figure S3B-C). According to the expression levels of gene markers, these cell clusters were annotated into 10 known cell types, namely, B cells, DC cells, macrophage cells, mast cells, NK cells, T cells, endothelial cells, epithelial cells, fibroblasts, and oligodendrocytes (Figure S3D and Fig. 1B). To differentiate conclusive malignant cells from potentially nonmalignant cell populations, we assessed chromosomal copy number variations (CNVs) in the whole transcriptome data (Figure S3E). Malignant cells and epithelial cells showed significantly higher epithelial scores than nonmalignant cells (Figure S3F), indicating that malignant cells originated from epithelial cells. As anticipated, the number of malignant cells increased as the cancer stage progressed (Fig. 1A). Furthermore, malignant cells were gathered to different clusters corresponding to their sample origin, whereas nonmalignant cells did not show distinguishable differences between patients (Fig. 1B-C and Figure S3C). Thus, the heterogeneity of malignant cells was the main contributor to the interpatient heterogeneity.

According to the expression data of 1,185 R-loop regulators [7], we conducted WGCNA and screened out four modules related to EGFR mutations, tissue origins, and TNM stage (Fig. 1D and Figure S3G). The KEGG enrichment analysis revealed that 92 regulators from the four modules were remarkably enriched in DNA damage/repair-associated pathways (Fig. 1E, Figure S3H, and Table S2). Based on the expression profiles of these identified genes, a scoring model was then constructed to characterize the R-loop state. Malignant cell subgroups (e.g., subgroups 2, 4, 6, 8, and 10) originating from metastatic samples showed low R-loop scores, indicating that low-scoring cells predominated in the malignant-cell composition of advanced tumors (Fig. 1F and Figure S3I). Clearly, the R-loop score in malignant cells was lower than that in other cell types (Figure S3J). We further observed that malignant cell clusters could be evidently separated according to the R-loop score, indicative of R-loop heterogeneity (Fig. 1F). Moreover, malignant cells from patients who received chemotherapy had distinctly high R-loop scores (Fig. 1G). The R-loop score of malignant cells was distinctly inversely associated with clinical tumor stage, and the score was significantly low in both patients with KRAS and TP53 mutations. The malignant cells were subsequently classified into groups with high or low R-loop scores according to the median R-loop score. Both groups presented a significant difference in the enriched pathway, of which tumor progression–related signaling pathways were mainly enriched in the low-score group (Fig. 1H). Meanwhile, malignant cells with low R-loop scores showed an enhanced proliferative state, with noticeably upregulated cell cycle–related proteins (Figure S3K), indicating cell cycle activation in these cells. Collectively, R-loops may play a key role in tumor progression.

### R-loop score characterizes tumor immune escape phenotypes

**Fig. 2:**
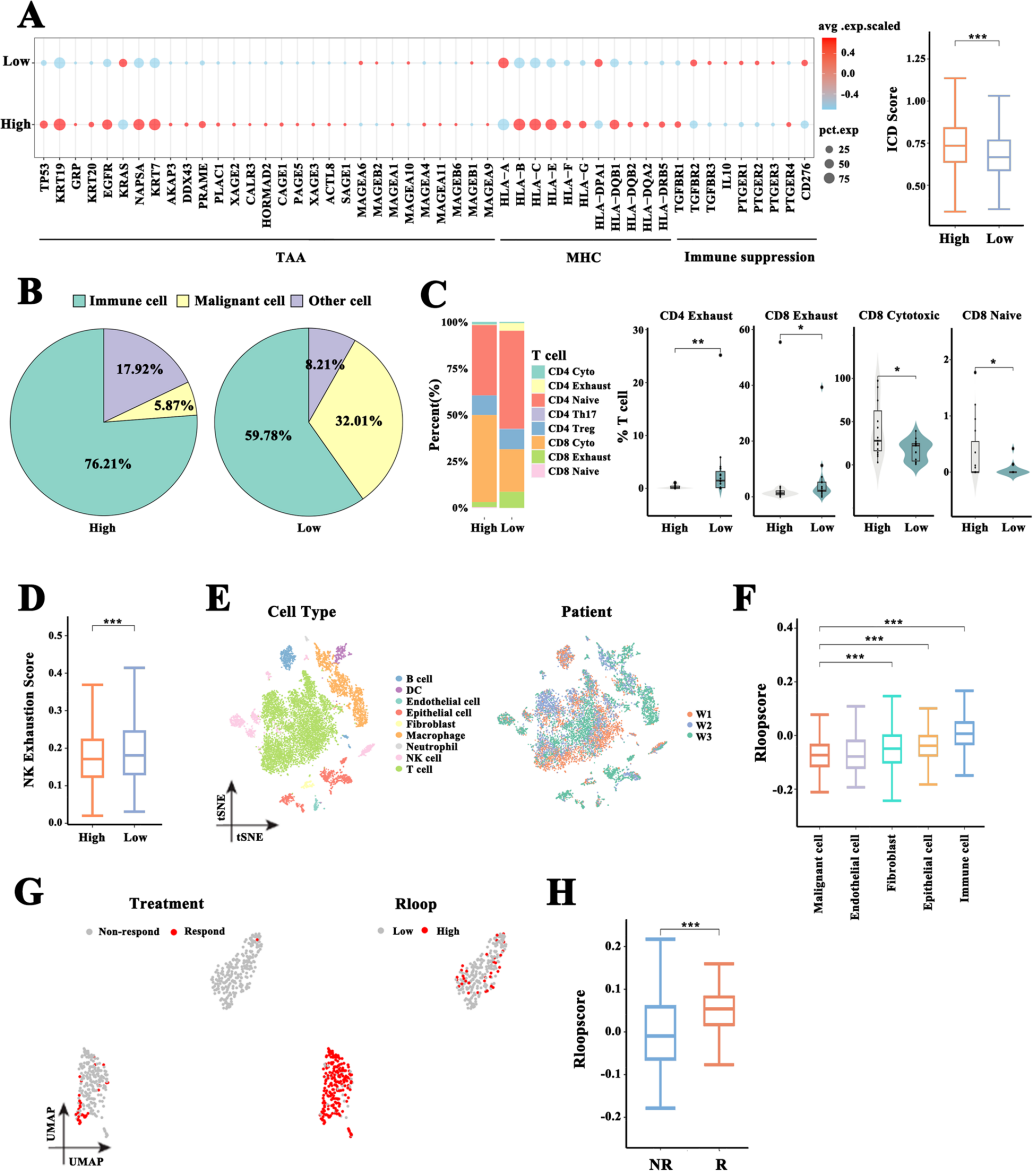
R-loop score positively correlated with anti-PD-1 immunotherapy response. **A** Changes in immune escape molecular profiles of malignant cells from GEO datasets (GSE123904 and GSE131907) between subgroups with high and low R-loop scores, and the difference in the score of enriched immunogenic cell death (ICD) pathways between two different scoring subgroups, using single-sample gene set enrichment analysis (ssGSEA, Wilcoxon rank test). **B** Proportions of immune cells, malignant cells, and other cells from samples with high R-loop scores and those with low R-loop scores. **C** Proportions of eight immune cell types and naive, cytotoxic, and dysfunctional T cells from patients with high R-loop scores and those with low R-loop scores (Wilcoxon rank test). **D** The difference in the NK cell exhaustion score between malignant cells with high and low R-loop scores (Wilcoxon rank test). **E** T-distributed stochastic neighbor embedding (t-SNE) plot of 11,515 cells from the single-cell RNA-sequencing data (GSE146100) colored according to cell types or individual patients. **F** R-loop scores for each cell type such as malignant cells, endothelial cells, fibroblasts, epithelial cells, and immune cells (Wilcoxon rank test). **G** Uniform manifold approximation and projection (UMAP) of malignant cells colored according to the treatment response or R-loop score. **H** The difference in the R-loop score between patients who responded to cancer treatment and those who did not (Wilcoxon rank test). (*p < .05, **p < .01, ***p < .001)

Given that immune escape is a critical factor in the inability of the immune system to control tumor progression [30], we investigated whether R-loops can affect immune escape according to the single-cell RNA-seq datasets (GSE123904 and GSE131907). Figure 2A illustrates changes in the molecular profiles of immune escape between the high and low R-loop score subgroups. Low R-loop scores downregulated the expression of tumor-associated antigens (TAA) and major histocompatibility complex (MHC) molecules but upregulated tumor-associated immunosuppressive factors in malignant cells. Using single-sample gene set enrichment analysis (GSEA), we discovered that the low R-loop subgroup had significantly low scores of immunogenic cell death pathway (Fig. 2A), suggesting a weak immunogenic ability [31]. The results revealed that malignant cells with low R-loop scores are potentially more likely to escape from immune response than those with high R-loop scores. Regarding functional consequences of the low-scoring R-loop in LUAD patients, dysfunctional CD4 + and CD8 + T cells, as well as the exhaustion scores of NK cells, remarkably increased (Fig. 2B-D). Considering that T and NK cell depletion in tumors is closely related to the immune escape of tumor cells [32], we validated these findings by collecting and examining bulk RNA-seq datasets from TCGA-LUAD samples. Patients with low R-loop scores had low-level infiltration of immune cells and displayed downregulation of immune response-related factors, costimulatory molecules, and MHC molecules and upregulation of coinhibitory molecules (Figure S4A-F), supporting the findings described above at the single-cell level. Malignant tumors may utilize multiple regulatory mechanisms to build a robust immunosuppressive microenvironment that supports tumor growth, promotes tumor immune escape, and impairs immunotherapy efficacy; hence, we evaluated the relationship of R-loop scoring with immunotherapy response, adopting the single-cell RNA-seq data (GSE146100). After dimensionality reduction clustering and annotation (Fig. 2E), the R-loop score for each cell type was calculated. As shown in Fig. 2F, malignant cells had a considerably low R-loop score as compared with the other cell types, including epithelial cells. In addition, malignant cells from patients who were responsive to anti-PD-1 immunotherapy were almost completely clustered into the high R-loop score subgroup (Fig. 2G). Notably, the R-loop scores of malignant cells in these patients were significantly higher than those in patients who were nonresponsive to such therapy (Fig. 2H). Altogether, R-loop score could characterize tumor immune escape phenotypes and serve as a predictor of the outcome of immunotherapy in LUAD.

### R-loop reshapes the intercellular interactions and cytokine signaling activity in TME

**Fig. 3:**
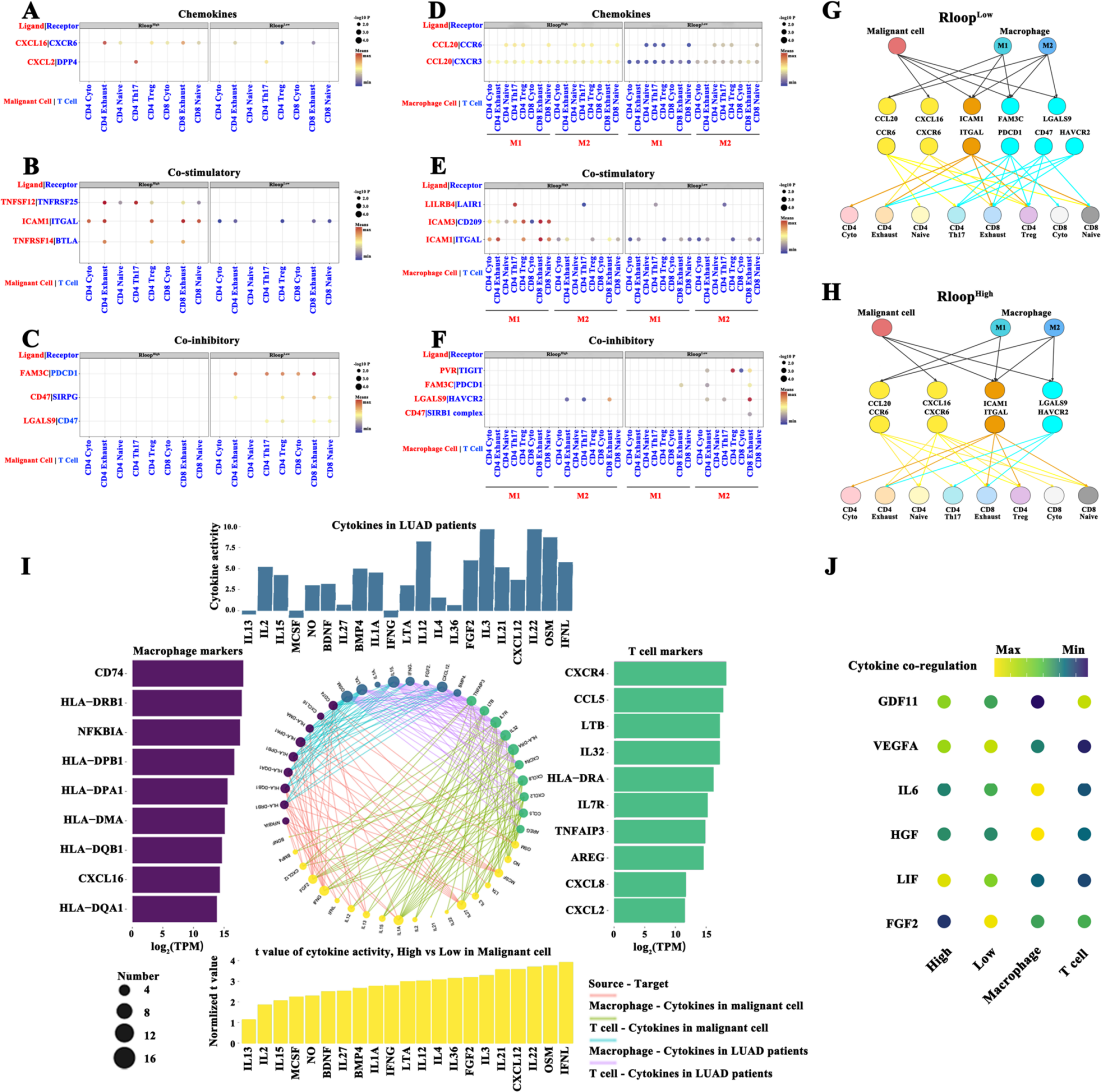
Cell communication and cytokine-associated pathway network analysis. **A**-**C** Intercellular interactions between malignant cells and T cell subtypes through chemokines (**A**), costimulatory molecules (**B**), and coinhibitory molecules (**C**). Circle size showing *p* values, and the color indicating the expression levels of ligand-receptor molecules. **D**-**F** Intercellular interactions between M1/M2-like macrophages and T cell subtypes via chemokines (**D**), costimulatory molecules (**E**), and coinhibitory molecules (**F**). **G**-**H** Ligand-receptor connections between malignant cells/macrophages and T cell subtypes under R-loop high-score (**G**) or low-score (**H**) conditions. Ligand-receptor molecules colored in yellow (chemokines), brown (costimulatory molecules), and cyan (coinhibitory molecules). **I** The activity of 21 cytokine pathways identified from malignant cells with high R-loop scores or patients with LUAD shows a positive relationship with macrophage marker and T cell marker genes (r > .2, *p* < .05). Circle size indicates the proportion of marker genes. **J** Signaling activities of multiple cytokines in malignant cells with high and low R-loop scores, T cells, and macrophages. The values of the activity of each cytokine are distinguished by color

Considering that intercellular interactions were a critical determinant of antitumor efficacy, we annotated T cell subtypes and macrophages (Figure S5A-F), followed by cell communication analysis using the single-cell RNA-seq datasets (GSE123904 and GSE131907). R-loop score remarkably positively correlated with chemokine levels from malignant cells such as CXCL16 and CXCL2 (Fig. 3A). Additionally, high R-loop scores clearly promoted costimulatory molecules including TNFSF12, ICAM1, and TNFRSF14 produced by malignant cells (Fig. 3B); meanwhile, coinhibitory signals in cells with R-loop high scores decreased appreciably (Fig. 3C). The communication between malignant cells and T cells through their coinhibitory molecules such as FAM3C and PDCD1, CD47 and SIRPG, and LGALS9 and CD47 was enhanced, contributing to T cell exhaustion. Moreover, CCL20 mediated the crosstalk between T cell subtypes and macrophages (Fig. 3D). Further analysis of the intercellular interaction between macrophages and T cells revealed that macrophages under R-loop score patterns showed differential effects on coinhibitory and costimulatory signals to T cells (Fig. 3E-F). Figure 3G-H depicts the difference in the malignant cell–macrophage cell communication under R-loop score patterns. Macrophages could act cooperatively with malignant cells to activate T cells with low R-loop scores, including exhausted T and Treg cells. Considering the essential role of cytokines in mediating intercellular communication, we explored whether cytokine signaling activity can be regulated by R-loops at single-cell levels. Consequently, we found 21 cytokine-related pathways that were considerably activated in malignant cells with high R-loop scores, and the levels of marker genes in T cells and/or macrophages were significantly positively associated with the activation of these pathways (Fig. 3I). For instance, CXCL2 overexpression in T cells was positively related to cytokine signaling in malignant cells such as FGF2 and IFNL. Especially, the differences in the activation of cytokine signaling pathways such as GDF11, VEGFA, and IL6 between high and low R-loop scores contributed to the synergistic regulatory effect within T cells and/or macrophages (Fig. 3J). Taken together, R-loop diversity indicates a difference in the immune composition of the TME, and R-loop could act as the main regulator of T cell activation by remodeling cell communication in LUAD.

### R-loop influences LUAD response to TKI therapy

**Fig. 4:**
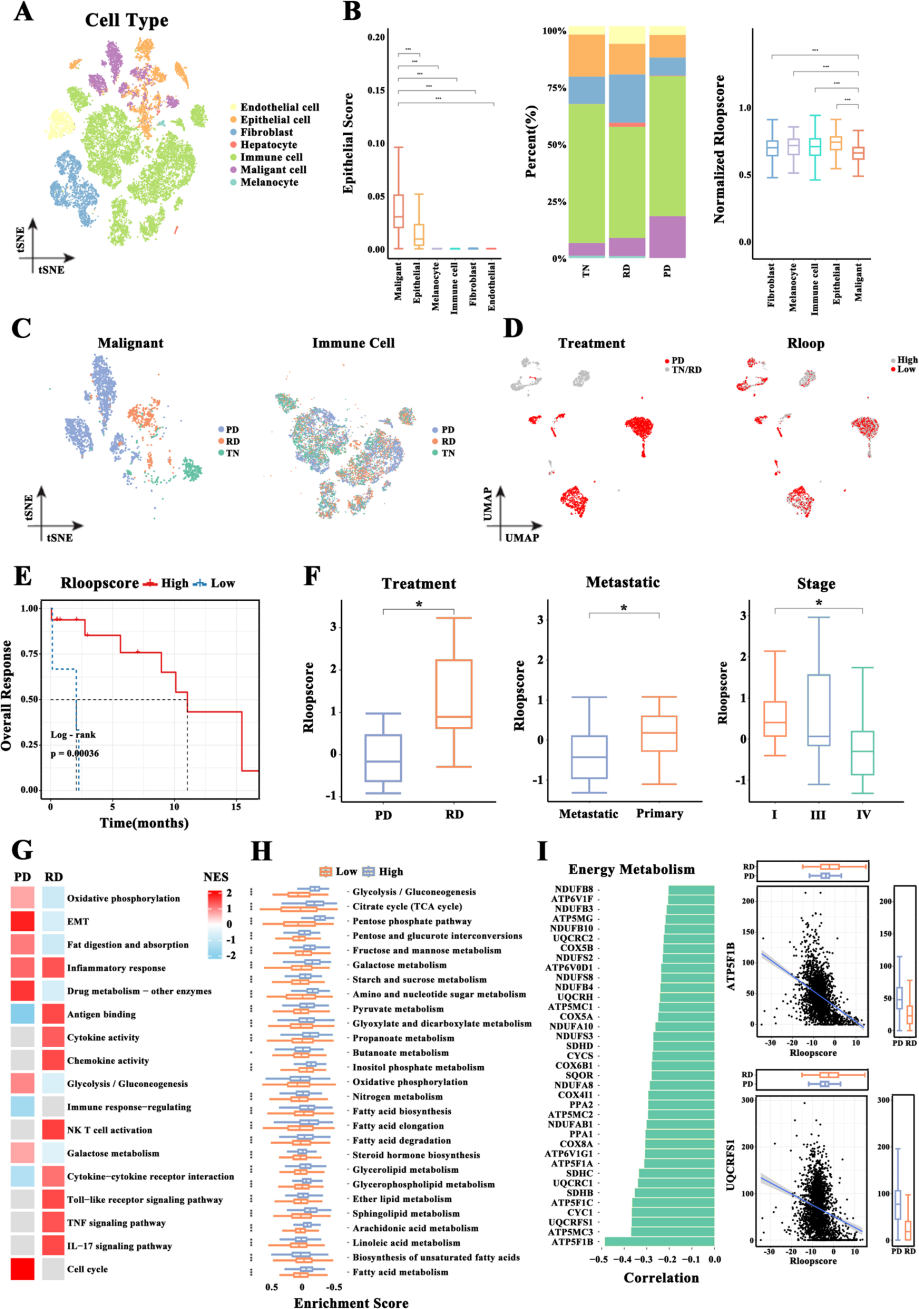
R-loop score positively correlated with tyrosine kinase inhibitor (TKI) treatment response. **A** Uniform manifold approximation and projection (UMAP) plot of 23,420 cells of 30 samples from the National Center for Biotechnology Information (NCBI) BioProject (PRJNA591860); they are colored according to cell type. **B** Epithelial scores for each cell type such as malignant cells, epithelial cells, melanocytes, immune cells, fibroblasts, and endothelial cells according to the expression of epithelial marker genes. The distribution of these cell types in patients with initiating targeted therapy (TN), complete or partial response state (RD), and progressive disease (PD). The differences in the R-loop score among these cell types (Wilcoxon rank test). **C** T-distributed stochastic neighbor embedding (t-SNE) plot of malignant cells or immune cells colored according to TKI subgroup. **D** UMAP plot of malignant cells colored according to TKI subgroup or R-loop score. **E** The difference in the overall survival rates between patients with high and low R-loop scores (log-rank test). **F** R-loop scores of cancer samples calculated according to the mean value of the total number of cells (Wilcoxon rank test). **G** The enriched pathways of malignant cells with high R-loop scores in PD and RD samples. Normalized enrichment score (NES) indicates the score for enriched signal pathways. **H** Gene set variation analysis (GSVA) showing the differences in the enriched metabolism–related pathways between malignant cells with high and low R-loop scores (Wilcoxon rank test). **I** Energy metabolism–associated genes negatively related to R-loop score. The scatterplot displaying the relationship between identified genes such as ATP5F1B and UQCRFS1, and R-loop score. The marginal boxplots on x-axis and y-axis show the distribution of R-loop scores and the expression level of identified genes, respectively. (*p < .05, **p < .01, ***p < .001)

Given that drug resistance limits the clinical efficacy of drugs such as TKI through metabolic reprogramming [33], we explored whether the therapeutic effect of drugs could be impacted by R-loops. We collected a single-cell dataset containing 49 tissue samples from 30 patients with LUAD treated with TKI from NCBI BioProject (PRJNA591860). These patients were divided into three subgroups according to the following time points: before initiating the targeted therapy (TN; *n* = 15), complete or partial response state (RD; *n* = 14), and progressive disease state (PD; *n* = 20). Tumor samples from the PD subgroup had gained resistance to TKI. Next, several cell clusters were obtained and respectively annotated to endothelial cells, epithelial cells, fibroblasts, hepatocytes, immune cells, and melanocytes (Fig. 4A). From the epithelial cells, 2,719 malignant cells were identified using inferCNV (Figure S6A-B). Analysis of cell cluster distributions among the TN, RD, and PD subgroups revealed that malignant cells were markedly highly specific for TKI and were significantly enriched in the PD subgroup (Fig. 4B-C and S6C-F). However, the immune cell distributions did not differ considerably between these subgroups. Subsequently, the R-loop scores of malignant cells were calculated according to the expression profiles of 92 R-loop regulators. Malignant cells with low R-loop scores mainly came from PD samples (Fig. 4D). Furthermore, patients with tumor (*n* = 22) were divided into two subgroups according to the R-loop scores of malignant cells. Patients with high R-loop scores had a significant advantage for survival (Fig. 4E). Conversely, metastatic and advanced tumors displayed a remarkably lower R-loop score, and the R-loop score of patients with RD was significantly high (Fig. 4F). However, the differences in the R-loop score were not influenced by patients’ age or sex (Figure S6G). Therefore, the R-loop score could be a strong predictor of TKI therapy efficacy.

Moreover, GSEA was separately conducted according to stratification factors such as the R-loop score and TKI result. The inflammatory response pathways and immune-related pathways were clearly enriched in RD samples with high R-loop scores, whereas metabolism-related pathways were significantly enriched in RD samples with low R-loop scores (Fig. 4G). Of these pathways, the activated inflammatory response signaling was associated with the R-loop score, regardless of the TKI result. Meanwhile, the metabolism-related pathways showed a clear difference in the activation between the PD and RD subgroups (Fig. 4G). We then focused on metabolism-related pathways and conducted GSVA according to the gene set of the previously defined pathways. The energy metabolic pathways responsible for tumor progression were significantly enriched in samples with low R-loop scores (Fig. 4H). Energy metabolism–associated genes such as ATP5F1B and UQCRFS1 were upregulated in the malignant cells of samples with low R-loop scores, and their expression was negatively related to R-loop score (Fig. 4I). Therefore, the determined link between energy metabolism and R-loop score possibly explains the drug tolerant state in malignant cells.

### R-loop score is closely linked to the prognosis of all cancers

**Fig. 5:**
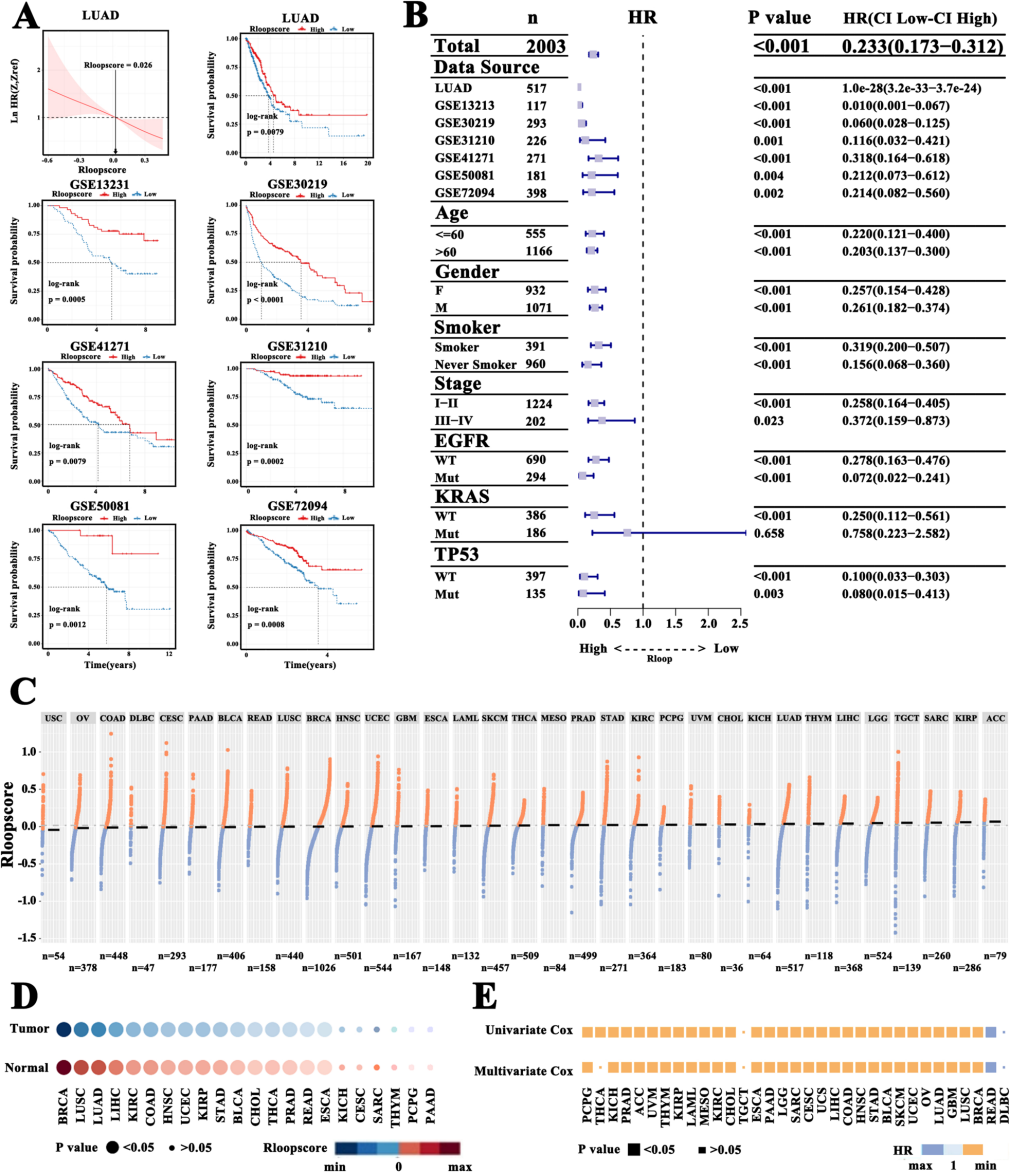
R-loop score correlated with prognosis. **A** Nonparametric estimates of the dependence of the all-time risk of mortality on R-loop scores using smoothHR, and the difference in the overall survival rate between patients with high and low R-loop scores (log-rank test). **B** Hazard ratios (HRs; 95% confidence intervals) based on Cox proportional hazard models (log-rank test). **C** R-loop scores across 33 tumor types in TCGA. **D** The difference in the R-loop score between tumor tissues and adjacent normal controls across 22 TCGA tumor types (log-rank test). **E** HRs (95% confidence intervals) based on univariate or multivariate Cox proportional hazards models in 33 tumor types (log-rank test)

Considering that R-loop plays a significant role in tumor progression, we evaluated the prognostic value of R-loop patterns. We first plotted the hazard ratio (HR) curve related to the R-loop score and found that the prognostic value of the R-loop score in seven independent cohorts of LUAD (TCGA-LUAD, GSE13213, GSE30219, GSE31210, GSE41271, GSE50081, and GSE72094) was linear (Fig. 5A and Figure S7A). The median value of the R-loop scores was set as the reference line. The 95% confidence interval (CI) of the Ln (HR) region was below the reference line when the R-loop score was less than the median value, and vice versa. Patients were classified into high- and low-score subgroups according to the median R-loop score. Kaplan–Meier survival analysis revealed that the high-score group had remarkably better outcomes than the low-score group in all LUAD cohorts (Fig. 5B and Figure S7B). Accordingly, R-loop score can act as a valuable prognostic marker for LUAD patients.

Moreover, we evaluated 9,757 tumor samples from 33 tumor types, including THCA, PRAD, PAAD, and invasive BRCA, from the TCGA dataset. The R-loop score for most of the tumor types was distributed similarly (Fig. 5C). Next, we divided these tumor samples into high- and low-score subgroups according to the median value of R-loop scores for each tumor type. Of note, tumor samples had a prominently reduced R-loop score compared with their matched adjacent normal tissues (Fig. 5D). Univariate and multivariate survival analyses further indicated that the low-score group consistently correlated with unfavorable outcomes in almost all tumor types (Fig. 5E). Thus, R-loop score classification could markedly predict cancer prognosis.

### R-loop score predicts therapeutic prognosis and therapeutic response

**Fig. 6:**
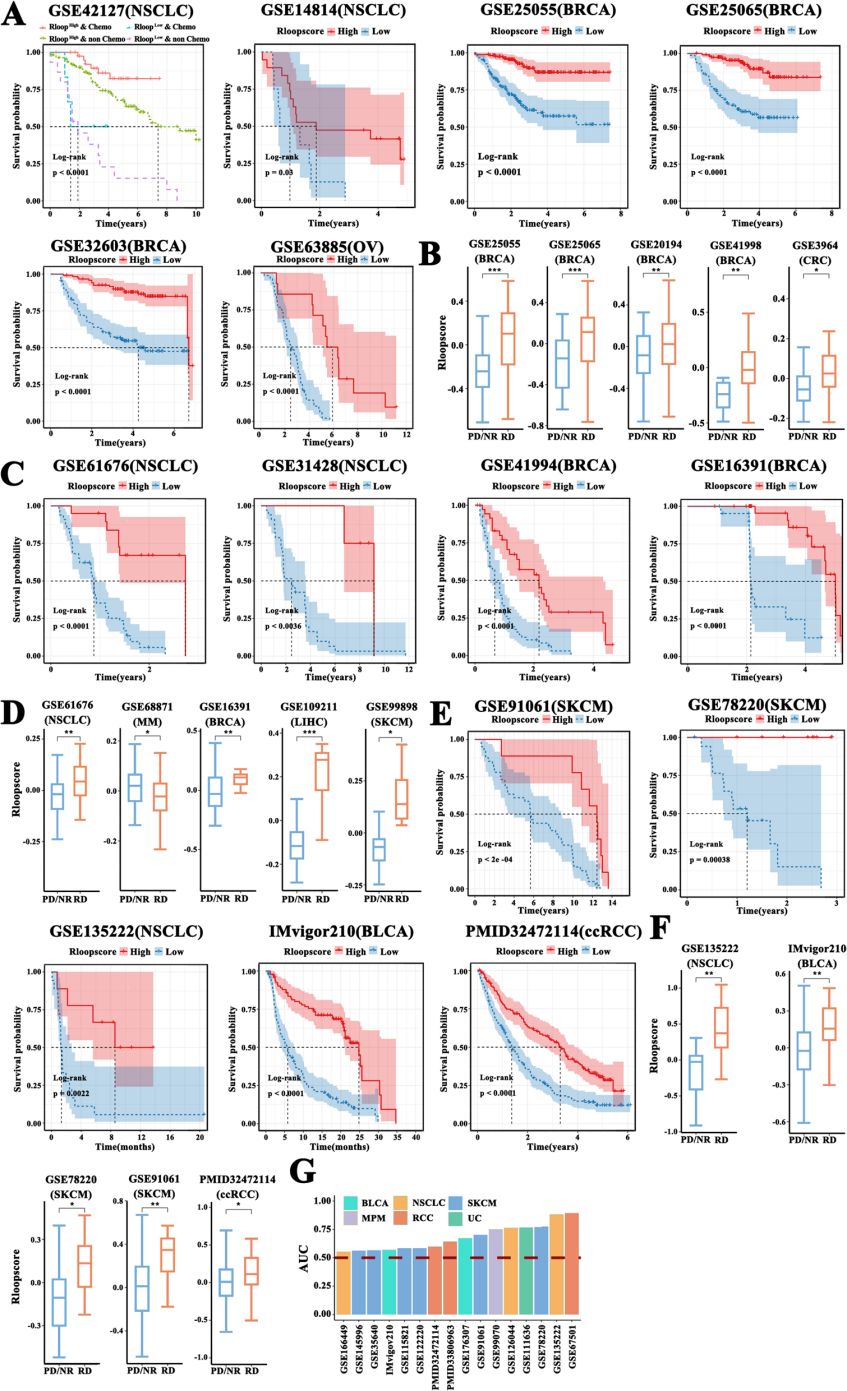
R-loop score correlated with prognosis and therapeutic response. **A** Comparison of overall survival probability according to R-loop score in chemotherapy-treated patients from GSE42127 (NSCLC), GSE14814 (NSCLC), GSE25055 (BRCA), GSE25056 (BRCA), GSE32603 (BRCA), and GSE63885 (OV) cohorts (log-rank test). **B** Comparison of R-loop score between the progressive disease/nonresponse (PD/NR) groups and the response (RD) group from GSE25055 (BRCA), GSE25056 (BRCA), GSE20194 (BRCA), GSE41998 (BRCA), and GSE3964 (CRC) cohorts who underwent chemotherapy (Wilcoxon rank test). **C** The difference in the overall survival probability between patients with high R-loop scores and those with low R-loop scores from GSE61676 (NSCLC), GSE31428 (NSCLC), GSE41994 (BRCA), and GSE16391 (BRCA) cohorts who received targeted therapy. **D** The difference in the R-loop score between PD/NR and RD samples from GSE61676 (NSCLC), GSE68871 (MM), GSE16391 (BRCA), GSE109211 (LIHC), and GSE99898 (SKCM) cohorts who underwent targeted therapy (Wilcoxon rank test). **E** The difference in the overall survival probability between patients with high and low R-loop scores from GSE78220 (SKCM), GSE135222 (NSCLC), IMvigor210 (BLCA), and PMID32472114 (ccRCC) cohorts who received immunotherapy. **F** The difference in the R-loop score between PD/NR and RD samples from GSE135222 (NSCLC), IMvigor210 (BLCA), GSE78220 (SKCM), GSE91061 (SKCM), and PMID32472114 (ccRCC) cohorts who underwent immunotherapy (Wilcoxon rank test). **G** The overall predictive accuracy of the R-loop scoring model for immunotherapy in 16 dependent datasets across 6 tumor types. (*p < .05, **p < .01, ***p < .001)

To explore the capacity of the R-loop score model to predict therapeutic effect in patients with cancer, we extracted 32 tumor RNA-seq datasets (including 11 tumor types) with chemotherapy, targeted therapy, or immunotherapy. Then, we divided the patients into two different score subgroups according to the cutoff value of the R-loop scores. Survival probabilities significantly differed between the two subgroups in six independent cohorts, namely, GSE42127 (NSCLC), GSE14814 (NSCLC), GSE25055 (BRCA), GSE25065 (BRCA), GSE32603 (BRCA), and GSE63885 (OV), undergoing chemotherapy (Fig. 6A). The overall survival of the high-score group was significantly superior to that of the low-score group. The performance of our model was then evaluated by analyzing the difference in the R-loop scores between patients with different degrees of chemotherapy response. Consistent with the results described above, the R-loop scoring displayed good classification performances on five cohorts such as GSE25055 (BRCA), GSE25065 (BRCA), GSE20194 (BRCA), GSE41998 (BRCA), and GSE3964 (CRC) undergoing chemotherapy (Fig. 6B). We also evaluated the predictive performance of the R-loop score model in patients receiving targeted therapy, and the independent cohorts included GSE61676 (NSCLC), GSE31428 (NSCLC), GSE41994 (BRCA), and GSE16391 (BRCA). As shown in Fig. 6C, the outcomes of the high-score group were significantly better than those of the low-score group. In addition, the RD group scored consistently higher than the other response-type groups in five cohorts, namely, GSE61676 (NSCLC), GSE68871 (MM), GSE16391 (BRCA), GSE109211 (LIHC), and GSE99898 (SKCM) (Fig. 6D). Moreover, patients who received immunotherapy in the high-score subgroup had a clearly favorable prognosis than those in the low-score group across five independent cohorts such as GSE91061 (SKCM), GSE78220 (SKCM), GSE135222 (NSCLC), IMvigor210 (BLCA), and PMID32472114 (ccRCC) (Fig. 6E). In five cohorts, namely, GSE135222 (NSCLC), IMvigor210 (BLCA), GSE78220 (SKCM), GSE91061 (SKCM), and PMID32472114 (ccRCC), the R-loop scores of the RD group were significantly higher than those in the NR or PD group (Fig. 6F). Figure 6G shows the area-under-the-curve values for the reliable performance of the R-loop scoring model in predicting immunotherapy prognosis and response. Collectively, the R-loop score could be an effective predictor of tumor treatment effects.

### FANCI affects R-loop distribution resulting in tumor progression in LUAD

**Fig. 7:**
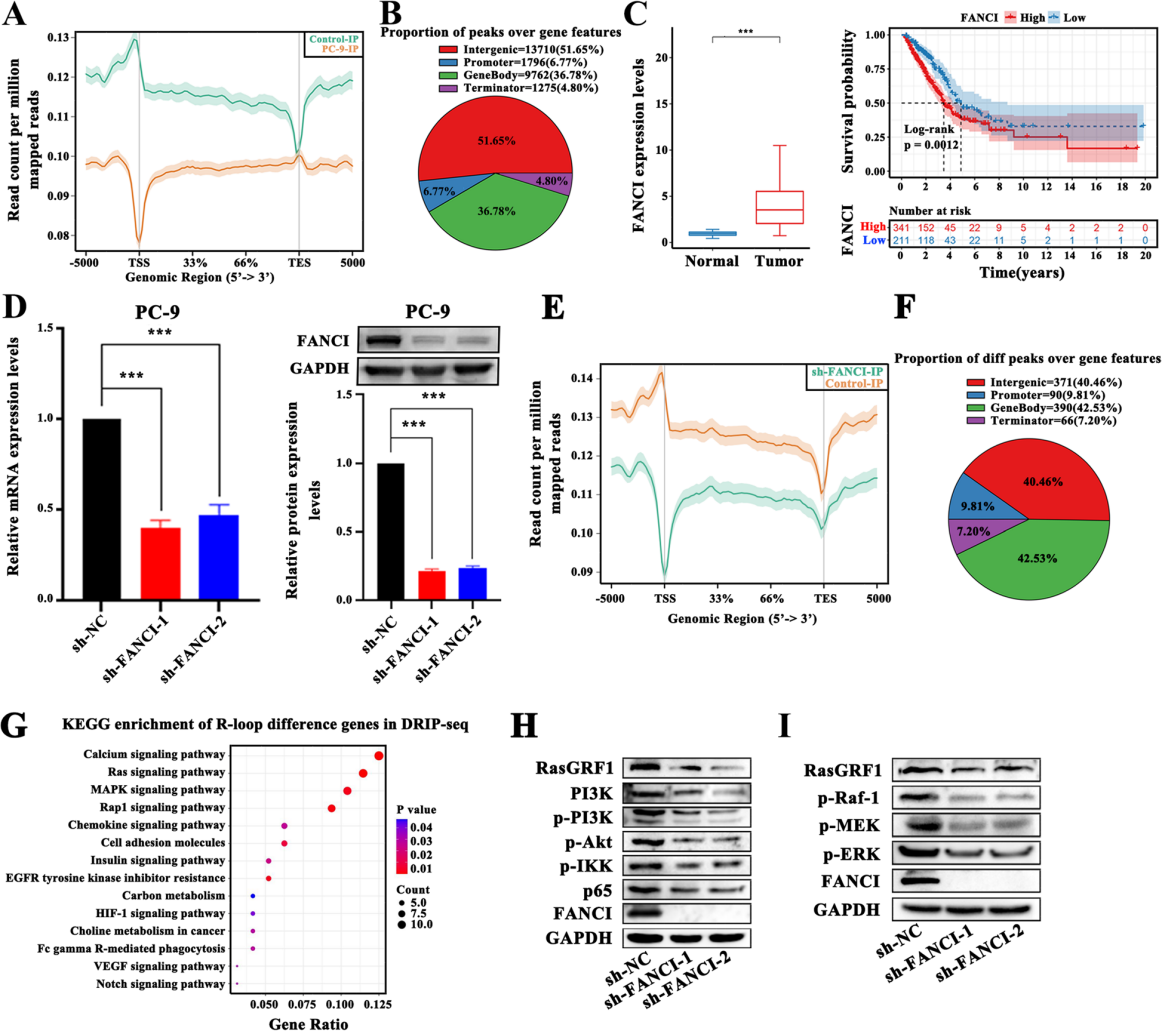
FANCI knockdown affected R-loop distribution inhibiting the Ras signaling pathway. **A** Genome-wide R-loop signal profile for PC-9. **B** The percentages of R-loop peaks are distributed as intergenic, promoter, genebody, and terminator regions. **C** The difference in FANCI expression between tumor and normal control tissues from TCGA-LUAD (Wilcoxon rank test), and the difference in the overall survival probability between patients with high and low FANCI expression (log-rank test). **D** shRNAs induced gene silencing, reducing FANCI mRNA and protein levels (Student’s *t*-test). **E** Comparison of R-loop signal profile between PC-9 and PC-9 with FANCI knockdown. **F** The proportion of different R-loop peaks in intergenic, promoter, genebody, and terminator regions between PC-9 and PC-9 with FANCI knockdown. **G** The Kyoto Encyclopedia of Genes and Genomes (KEGG) analysis for genes with a statistically significant difference in the R-loop distribution between control and FANCI-knockdown PC-9 cells. **H**-**I** FANCI silencing decreased the protein levels of RasGRF1, p-PI3K, p-AKT, p-IKK, p-65 (H), and p-Raf1, p-MEK, and p-ERK in PC-9 cells (**I**). Transcription start site (TSS) and transcription termination site (TTS). (*p < .05, **p < .01, ***p < .001)

**Fig. 8:**
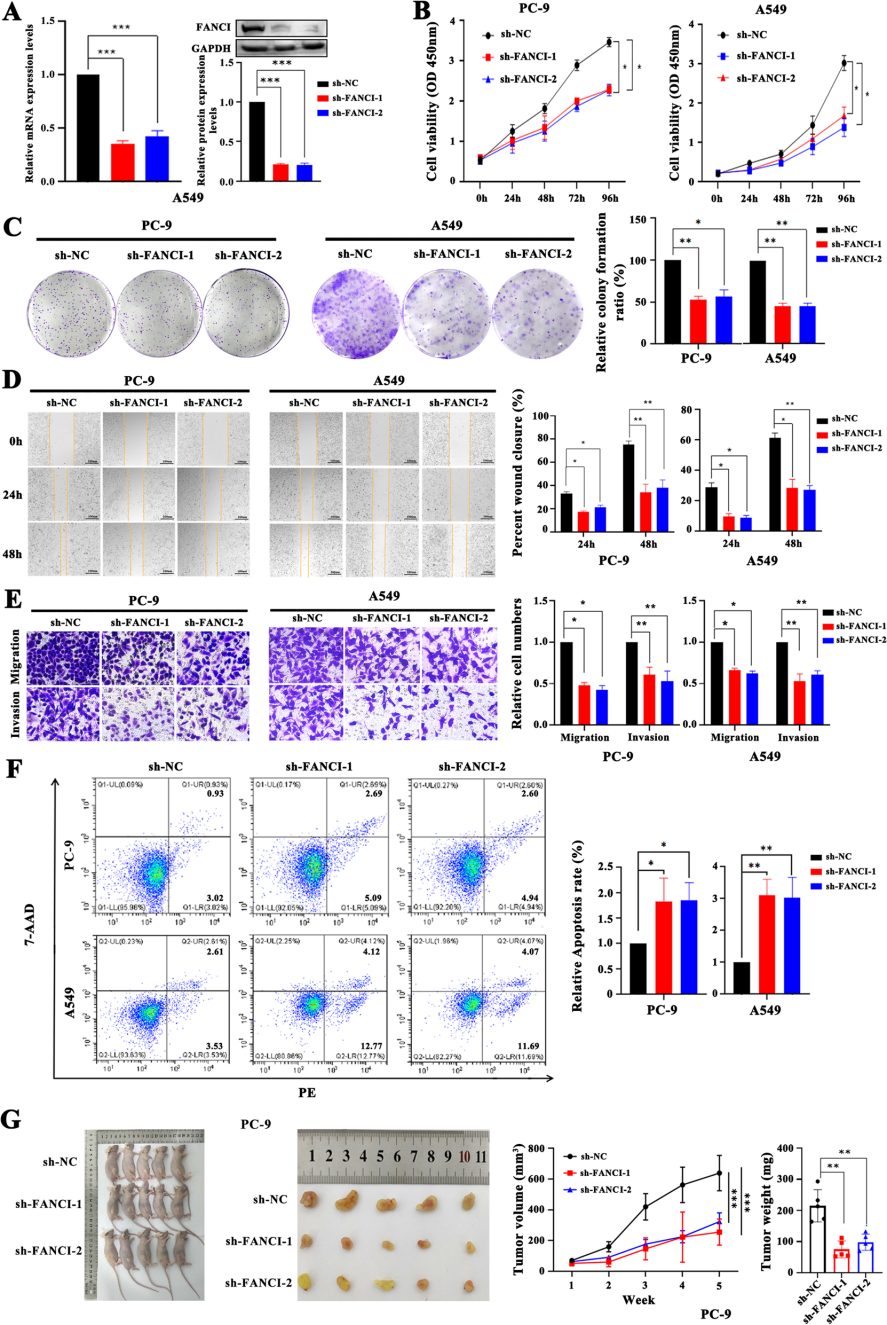
FANCI knockdown limited cell proliferation, colony formation, migration, invasion, and tumor-forming ability and promoted cell apoptosis. **A** shRNAs induced gene silencing, reducing FANCI mRNA and protein levels in A549. (B-E) FANCI knockdown clearly inhibited the proliferation (**B**), colony formation (**C**), scratch healing (**D**), migration, and invasion (**E**) abilities of PC-9 and A549. **F** FANCI silencing promoted apoptosis of PC-9 and A549. **G** FANCI silencing suppressed the tumor-forming ability of PC-9. (Student’s *t*-test; *p < 0.05; **p < 0.01; ***p < 0.001)

To plot the R-loop landscape of LUAD cells, we conducted DRIP-seq using S9.6, an anti-DNA/RNA hybrid antibody. In LUAD cell lines (PC-9), genome-wide R-loop signal profile showed that the number of reads at the peak was higher in promoter regions than in other regions (Fig. 7A). Out of the 26,534 R-loop peaks genome-wide, approximately half of the peaks were in intergenic regions, while 11.57% were located at promoter and terminator regions (Fig. 7B). FANCI, an R-loop regulator, was upregulated in almost all TCGA tumor types, including LUAD, and its high expression correlated with poor prognosis (Fig. 7C and Figure S8A-B). We next tested the effect of FANCI deficiency on R-loop distribution (Fig. 7D) and found that FANCI knockout clearly altered the R-loop landscape, showing a strong loss trend (Fig. 7E). We then analyzed the differences in the number of R-loop peaks (Fig. 7F). Such changes were observed in over all types of genic regions, such as intergenic (40.46%), genebody (42.53%), promoter (9.81%), and terminator (7.20%) regions. Furthermore, genes with R-loop changes were significantly enriched in cancer progression–associated pathways, such as Ras and MAPK signaling pathways (Fig. 7G, and Table S3); thus, FANCI may be involved in LUAD progression by regulating the R-loops. We further detected the activity of the Ras signaling pathway and found that silencing FANCI clearly attenuated the activity of Ras, leading to the strong inhibition of its downstream PI3K/AKT/NF-κB and MAPK/ERK signaling cascades in LUAD cells (Fig. 7H-I, and Figure S9). Finally, cell-function experiments revealed that FANCI depletion significantly suppressed cell proliferation, colony formation, migration, and invasion abilities, and promoted apoptosis in both PC-9 and A549 cells (Fig. 8A-F). Xenotransplantation studies indicated that FANCI silencing limited the capacity of PC-9 cells to form tumors in *vivo* (Fig. 8G). Altogether, changes in R-loop distribution mediated by an R-loop regulator such as FANCI could lead to LUAD development.

## Discussion

Although increasing evidence indicates that aberrant R-loops are closely linked to the progression of different cancer types, including LUAD [11, 34, 35], the causality remains unknown. This study demonstrated that R-loop scores from malignant cells could predict survival outcomes and treatment responses, and it also provided insights into the mechanisms of low-scoring R-loops mediating immune escape and drug resistance. Moreover, our analysis revealed that changes in R-loop distribution mediated by FANCI deficiency blocks the activity of the Ras signaling pathway, thereby suppressing tumor-cell proliferation and dissemination. Importantly, our study highlights the causal role of FANCI-mediated changes in R-loop distribution that leads to LUAD development.

R-loop-mediated replication stress and genomic instability may actively drive tumor development [36, 37]. In this study, we constructed an R-loop scoring model based on the identified R-loop regulators that lie within four modules related to EGFR mutations, tissue origins, and TNM stage. The R-loop score of malignant cells was significantly distinct from that of nonmalignant cells, indicating that the R-loop score is associated with the degree of cell malignancy. In addition, the R-loop score strikingly correlated with KRAS and TP53 mutations, tumor metastasis, tumor stage, cancer-related pathways, and prognosis. Therefore, R-loops are responsible for tumor progression. Furthermore, the R-loop score was found to be associated with patient outcomes and responsiveness to anticancer treatments.

As a dynamic system orchestrated by cell–cell communications, the TME is necessary for cancer progression and metastasis [38]. We built the cellular communication network of malignant cells and immune cells under R-loop scoring patterns within the TME according to the gene expression profile of individual LUAD cells. Among the immune cell types in the TME, Treg cells, which are essential for immune homeostasis, maintain immune self-tolerance and inhibit anticancer immunity [39], while CD8 + T are cytotoxic T lymphocytes killing tumor cells and may become exhausted during tumor progression [40]. Our results showed that low-scoring R-loops promoted CD8 + T cell exhaustion and CD4 + Treg cell formation. The enhanced levels of chemokines and coinhibitory molecules between M2-like macrophages and T cells in the low-score subgroup also suggested that tumor-associated macrophage accumulation inhibited T cell responses, leading to compromised tumor immune surveillance, anticancer efficacy, and even poor response to immune checkpoint blockade therapy. Our results also imply that an immunosuppressive microenvironment mediated by R-loops contributes to tumor immune escape and that tumor immune escape mechanisms restrict immunotherapy responses. One of the major challenges in tumor immunotherapy is that its effects are limited to a subset of patients [41]. In our study, low-scoring R-loops mediated multiple immune escape mechanisms, and patients who were nonresponsive to cancer treatment had significantly low R-loop scores. Thus, R-loop score can offer guidance to develop a personalized treatment plan for immune therapies, providing a complement to markers that are currently used to identify patients eligible for immunotherapy.

Moreover, molecularly targeted treatment against malignant cells reportedly can promote tumor evolution by targeting the drug-resistant clones [42]. Malignant cells known as drug-tolerant persister cells can enter a drug-tolerant state in response to lethal stress such as TKI therapy, thereby escaping apoptosis and surviving [43]. Our results showed that malignant cells with low R-loop scores displayed elevated levels of oxidative phosphorylation and tricarboxylic acid cycle, activation of epithelial–mesenchymal transition, and immune escape signatures such as the downregulation of TAA and MHC-I molecules and the upregulation of tumor-associated immunosuppressive factors, suggestive of a persister cell phenotype. Hence, we hypothesized that drug resistance mediated by metabolic reprogramming under R-loop score patterns is associated with malignant cell transformation and that low-scoring R-loops boost the development of drug resistance in malignant cells.

Our results showed that R-loop score was a potential prognosis predictor of LUAD, and it also could be used to predict the prognosis of pan-cancer patients. Moreover, the R-loop score model constructed in this study performed well on multiple independent datasets for predicting therapeutic prognosis and therapeutic response, including seven targeted therapy, six chemotherapy, and five immunotherapy cohorts. Therefore, our study highlights the potential promotion value of R-loop scoring in contributing to the establishment of precision medicine for cancer.

R-loops are associated with the progression of many cancer types [34, 35], including LUAD. Knowledge on the causal relationship between R-loops and disease etiology provides theoretical support for the development of R-loop-based targeted cancer therapeutic strategies [44–46]. FANCI family has been reported to play an important role in R-loop regulation [47]. Liang et al. found that FANCI, one of the members of the FANCI family, forms a FANCI-FANCD2 complex by binding to FANCD2, consequently regulating the R-loop balance [48]. FANCI is also actively involved in LUAD progression [49, 50]. Our results showed that FANCI knockout triggered changes in R-loop distribution and that genes with R-loop changes were strikingly enriched in tumor progression–associated pathways, including Ras, HIF, and VEGF signaling pathways. R-loops in the promoter and/or terminator regions can regulate gene expression through multiple mechanisms [12]. Furthermore, silencing FANCI attenuated the activity of oncogenic Ras signaling that acts as a tumor progression marker, thereby strongly inhibiting its downstream MAPK/ERK and PI3K/AKT/NF-κB signaling cascades in LUAD cells; it also suppressed the ability of tumor-cell proliferation and dissemination. Thus, R-loop distribution mediated by FANCI could participate in LUAD progression by affecting the Ras signaling pathway. Moreover, various ligands, such as netropsin and paramomycin, can recognize RNA/DNA hybrids by binding to the nucleic acid groove, thereby targeting these hybrids [51, 52]. RNase H2, an RNA–DNA degrading enzyme, is a well-recognized anticancer drug target [53]. Therefore, R-loops and R-loop regulators are both potential targets for LUAD therapy.

## Conclusion

In conclusion, we comprehensively analyzed R-loop distribution patterns in TME and uncovered that the distribution of low-scoring R-loops displayed an immunosuppressive microenvironment mediated by T cell exhaustion, thereby promoting tumor progression, and provided insights into the mechanisms of immune escape and metabolic reprogramming–mediated drug tolerance under low-scoring R-loop distribution. The results of this study improve our understanding on metabolic heterogeneity and TME reshaping mediated by R-loop distribution. This study further highlighted the causal role of abnormal R-loops in cancer development. Such knowledge is critical for designing individualized treatment strategies and may guide the development of more effective treatment options for LUAD on the basis of R-loop or R-loop regulators.

### Supplementary Information

**Additional file 1.** Supplementary Code.

**Additional file 2:** **Figure S1.** Systematic literature search in the databases PubMed. Numbers show the results acquired for each step.

**Additional file 3:** **Figure S2.** Overview of study design.

**Additional file 4:** **Figure S3. **Determining cell types for integrated cohorts (GSE123904 and GSE131907). (A) The proportion of cells in each patient. (B) The number of principal components for clustering. (C) Uniform manifold approximation and projection (UMAP) plot of 92,842 cells colored according to cell subgroup. (D) Average and percentage of the expression of marker genes in 57 subgroups. (E) Relative expression intensity in each chromosome identified by inferCNV. Amplifications and deletions on the indicated chromosomes shown in red and blue, respectively. (F) Epithelial scores for each cell type such as malignant cells, epithelial cells, melanocytes, immune cells, fibroblasts, and endothelial cells according to the expression of epithelial marker genes (Wilcoxon rank test). (G) Association between module eigengenes and clinical characteristics examined using weighted gene coexpression network analysis (WGCNA; Pearson’s correlation test). (H) Gene Ontology (GO) biological process (BP) terms enriched in 92 R-loop regulators. (I) UMAP plot of malignant cells colored according to sample origins. (J) R-loop score in each cell type (Wilcoxon rank test). (K) Proportions of malignant cells in G1, G2M, and S stages from high- and low-score groups, and the difference in the expression of cyclin genes between these two groups (Wilcoxon rank test). (*p < .05, **p < .01, ***p < .001).

**Additional file 5:** **Figure S4. **Differences in the immune microenvironment of patients from TCGA-LUAD in the R-loop score subgroups. (A) Patients with LUAD were divided into high- and low-score subgroups according to the median value of R-loop scores. Comparison of immune score, stromal score, ESTIMATE score, and tumor purity between the high- and low-score subgroups. (B) The difference in the enrichment scores of immune cells between the two subgroups. (C-F) The expression profiles of immune response–related factors (C), costimulatory molecules (D), antigen presentation molecules (E), and coinhibitory molecules (F) in the high- and low-score subgroups. (Wilcoxon rank test; *p < .05, **p < .01, ***p < .001).

**Additional file 6:** **Figure S5. **Subpopulations of T cells and macrophage cells from GSE123904 and GSE131907. (A) Uniform manifold approximation and projection (UMAP) plot of 31,201 T cells colored according to cell subclusters. (B) Average and percentage of the expression of marker genes in the T cell subclusters. (C) UMAP plot of T cells colored according to T cell type. (D) T-distributed stochastic neighbor embedding (t-SNE) plot of 14,737 macrophage cells colored according to cell subclusters. (E) Average and percentage of the expression of marker genes in the macrophage subclusters. (F) Subpopulations of the identified macrophage cells.

**Additional file 7:** **Figure S6. **Identification of malignant cells from patients who underwent tyrosine kinase inhibitor (TKI) therapy (PRJNA591860). (A) Relative expression intensity in each chromosome identified by inferCNV (all cell types). Amplifications and deletions on the indicated chromosomes shown in red and blue, respectively. (B) Copy number variation (CNV) scores for each epithelial cell subgroup. (C) Relative expression intensity in each chromosome (epithelial cells). (D) The difference in the CNV scores among patients classified into the initiating targeted therapy (TN), complete or partial response state (RD), and progressive disease (PD) subgroups. (E) CNV levels calculated by the quadratic sum of CNV regions for malignant and nonmalignant subgroups. (F) Uniform manifold approximation and projection (UMAP) plot of the PD, RD, and TN subgroups colored according to cell type. (G) R-loop scores of age, sex, and smoking status subgroups according to the mean value of all malignant cells. (Wilcoxon rank test; *p < .05, **p < .01, ***p < .001).

**Additional file 8:** **Figure S7. **Clinical characteristics of multiple factors in the independent cohorts. (A) Nonparametric estimates of the dependence of all-time risk of mortality on R-loop score using smoothHR, and the difference in the overall survival rate between patients with high and low R-loop scores. (B) Hazard ratios (HRs; 95% confidence intervals) based on Cox proportional hazard models (log-rank test).

**Additional file 9:** **Figure S8. **FANCI was highly expressed in all cancer types, and its high expression was associated with poor outcomes. (A) The difference in FANCI expression between tumor and normal control tissues in 31 cancer types (Wilcoxon rank test). (B) The difference in the overall survival probability between patients with high and low FANCI expression (log-rank test). (*p < .05, **p < .01, ***p < .001).

**Additional file 10:** **Figure S9.** FANCI silencing decreased the protein levels of RasGRF1, p-Raf1, p-MEK, p-ERK, PI3K, p-PI3K, p-AKT, p-IKK, and NFκB in PC-9 cells. (Student’s t-test; *p < 0.05; **p < 0.01; ***p < 0.001).

**Additional file 11:** **Table S1.** Details of datasets used in this study.

**Additional file 12:** **Table S2.** The list of R-loop regulators.

**Additional file 13:** **Table S3.** Differential R-loop peaks.

## Data Availability

The data of the relevant dataset in this study can be obtained by contacting the corresponding author. The basic raw NGS has been uploaded to the GEO database (GSE248308).

## Abbreviations

LUAD: Lung adenocarcinoma
TME: Tumor microenvironment
RNA-seq: RNA-sequencing
GEO: Gene Expression Omnibus
TKI: Tyrosine kinase inhibitor
NCBI: National Center for Biotechnology Information
TCGA: The Cancer Genome Atlas
THCA: Thyroid carcinoma
PRAD: Prostate adenocarcinoma
PAAD: Pancreatic adenocarcinoma
BLCA: Bladder urothelial carcinoma
BRCA: Breast cancer
CHOL: Cholangiocarcinoma
DLBC: Lymphoid neoplasm diffuse large B-cell lymphoma
GBM: Glioblastoma multiforme
HNSC: Head and neck squamous carcinoma
LIHC: Liver hepatocellular carcinoma
OV: Ovarian serous cystadenocarcinoma
SKCM: Skin cutaneous melanoma
FPKM: Fragments per kilobase million
WGCNA: Weighted gene coexpression network analysis
UMAP: Uniform manifold approximation and projection
t-SNE: T-Distributed stochastic neighbor embedding
GSVA: Gene set variation analysis
KEGG: Kyoto Encyclopedia of Genes and Genomes
NSCLC: Non-small-cell lung cancer
RPMI-1640: Roswell Park Memorial Institute 1640
Q-PCR: Quantitative real-time PCR
DRIP-seq: DNA/RNA immunoprecipitation and sequencing
PCA: Principal component analysis
CNVs: Copy number variations
TAA: Tumor-associated antigens
MHC: Major histocompatibility complex
GSEA: Gene set enrichment analysis
TN: Initiating targeted therapy
RD: Complete or partial response state
PD: Progressive disease state
HR: Hazard ratio
CI: Confidence interval
