# Supplementary material for: Aberrant R-loop–mediated immune evasion, cellular communication, and metabolic reprogramming affect cancer progression: a single-cell analysis
Source: Mol Cancer. 2024 Jan 10;23:11. doi: 10.1186/s12943-023-01924-6 (PMC10777569; doi:10.1186/s12943-023-01924-6)
Supplement: Supplementary file 1 — Additional file 1. Supplementary Code. [file 12943_2023_1924_MOESM1_ESM.pdf]

```
#fig1----
#data----
setwd("D:\\GSE131907")
library(data.table)
library(Seurat)
UMI = fread("GSE131907_Lung_Cancer_raw_UMI_matrix.txt",data.table = F)
rownames(UMI) = UMI[,1]
UMI = UMI[,-1]
scRNA = CreateSeuratObject(UMI, min.cells = 3, project =
"GSE131907",min.features =300)

ann = read.table("GSE131907_Lung_Cancer_cell_annotation.txt", header=T,
sep="\t", check.names=F, row.names=1)
ann = ann[colnames(scRNA),]
scRNA@meta.data$Sample = ann$Sample

meta = scRNA@meta.data
id = meta[meta$Sample=="LUNG_T09" | meta$Sample=="LUNG_T08" |
meta$Sample=="LUNG_T25" | meta$Sample=="LUNG_T06" |
            meta$Sample=="LUNG_T34" | meta$Sample=="LUNG_T31" |
meta$Sample=="LUNG_T19" | meta$Sample=="LUNG_T20" |
            meta$Sample=="LUNG_T18" | meta$Sample=="LUNG_T28" |
meta$Sample=="NS_06" | meta$Sample=="NS_16" |
            meta$Sample=="NS_02" | meta$Sample=="NS_19" |
meta$Sample=="NS_17" | meta$Sample=="NS_04" |
            meta$Sample=="NS_13" | meta$Sample=="NS_03" |
meta$Sample=="NS_12" | meta$Sample=="NS_07" |
            meta$Sample=="LUNG_T30",]

scRNA1 = scRNA[,rownames(id)]
save(scRNA1,file='scRNA1.Rdata')


setwd("D:\\GSE123904")

samples=list.files("./")
samples
dir <- file.path('./',samples)
names(dir) <- samples
names(dir)=str_split(names(dir),'_',simplify = T)[,3]

scRNAlist <- list()
for(i in 1:length(dir)){
  A = fread(dir[i])
  A = as.data.frame(A)
  rownames(A) = A[,1]
  A = as.data.frame(t(A[,-1]))
  scRNAlist[[i]] <- CreateSeuratObject(A, min.cells = 3, project =
names(dir)[i],min.features =300)
}

scRNA2 <- merge(scRNAlist[[1]], y=c(scRNAlist[[2]],
scRNAlist[[3]],scRNAlist[[4]],scRNAlist[[5]],

scRNAlist[[6]],scRNAlist[[7]],scRNAlist[[8]],scRNAlist[[9]]))

scRNA = merge(scRNA1,y=scRNA2)

### SCT
scRNA <- SCTransform(scRNA)
```

```
### PCA
scRNA <- RunPCA(scRNA, npcs=50, verbose=FALSE)

scRNA <- RunHarmony(scRNA, group.by.vars="orig.ident", max.iter.harmony =
20,assay.use = "SCT")

pc.num=1:30
scRNA <- RunTSNE(scRNA, reduction="harmony", dims=pc.num) %>%
RunUMAP(reduction="harmony", dims=pc.num)

scRNA <- FindNeighbors(scRNA, dims = pc.num)

scRNA <- FindClusters(scRNA, resolution = 1.5)

table(scRNA@meta.data$seurat_clusters)
library(SingleR)

cellpred <- SingleR(test = testdata, ref = refdata, labels =
refdata$label.main,
                    method = "cluster", clusters = clusters,
                    assay.type.test = "logcounts", assay.type.ref =
"logcounts")

celltype = data.frame(ClusterID=rownames(cellpred),
celltype=cellpred$labels, stringsAsFactors = FALSE)
scRNA@meta.data$singleR=celltype[match(clusters,celltype$ClusterID),'cell
type']

save(scRNA,file='scRNA.Rdata')


#A----
library(ComplexHeatmap)
library(circlize)
cli=read.table("Cli.txt",sep="\t",header=T,check.names=F,row.names = 1)

value = rnorm(19)
cli = cli[,-1]
colnames(cli)
ha = HeatmapAnnotation(df = cli,
                       col = list(
                         `Data Source` = c("GSE131907" = "#E69394" ,
"GSE123904" = "#BEBADA"),
                         `Sample Origins` =  c("Primary" = "#B3E2CD" ,
"Distant Metastasis" = "#E4D4B7", "Chemotherapy" = "#ECCFC0"),
                         Smoking =  c("Never smoker" = "#2DB600","Current
smoker" = "#EDB48E", "Former smoker" = "#E6E600"),
                         EGFR = c("WT" = "#7FC97F", "Mut" = "#FDC086",
"na" = "#A9B7B7"),
                         KRAS = c("WT" = "#7FC97F", "Mut" = "#FDC086",
"na" = "#A9B7B7"),
                         TP53 = c("WT" = "#7FC97F", "Mut" = "#FDC086",
"na" = "#A9B7B7"),
                         Stage = c("Stage I" = "#EFF3FF", "Stage II" =
"#B8D4E6", "Stage III" = "#64A9D3", "Stage IV" = "#2A7AB7")
                       ))
draw(ha)
```

```
meat=read.table("Paint_Malignant cells.txt", header=T, sep="\t",
check.names=F)
meat$x <- factor(meat$id,levels=c("P1","P2","P3","P4","P5",
                              "P6","P7","P8","P9","P10",
                              "P11","P12","P13","P14","P15",
                              "P16","P17","M1","M2","M3",
                              "M4","M5","M6","M7","M8",
                              "M9","M10","C1","C2","C3"))

meat$number = log2(meat$number+1)

ggplot(meat, aes(x=x, y=number, group = 1)) +
  geom_line(size=2,color="#CBD5E8")+
  geom_point(size=3)+
  theme(axis.text.x = element_text(angle = 90))+
  labs(x="",y="log2(Malignant Cell Number)")+
  theme(panel.grid.major =element_blank(),
        panel.grid.minor = element_blank(),
        panel.background = element_blank(),
        panel.border = element_blank())


meta = scRNA@meta.data
meta = meta[,c(1,10)]

for (i in 1:nrow(meat)) {
  x = meat[i,1]
  y = meat[i,2]
  meta$orig.ident[which(meta$orig.ident == y)] <- x
}

meta$x <- factor(meta$orig.ident,levels=c("P1","P2","P3","P4","P5",
                                    "P6","P7","P8","P9","P10",
                                    "P11","P12","P13","P14","P15",
                                    "P16","P17","M1","M2","M3",
                                    "M4","M5","M6","M7","M8",
                                    "M9","M10","C1","C2","C3"))

ggplot(data = meta, aes(x = x, fill =singleR1))+
  geom_bar(stat = 'count',position = 'fill')+labs(y = "Cell
Proportion(%)" , x="")+
  scale_fill_manual(values = c( "#80B1D3","#BC80BD" , "#FB8072"
,"#8DD3C7", "#FFFFB3",
                               "#FDB462" ,"#D9D9D9","#FCCDE5",
"#BABADA","#B3DE69"))+
  theme(axis.text.x = element_text(angle = 90))+
  theme(panel.grid.major =element_blank(),
        panel.grid.minor = element_blank(),
        panel.background = element_blank(),
        panel.border = element_blank())



#B----
DimPlot(scRNA, group.by="singleR1",  label.size=5, reduction='umap')
DimPlot(scRNA, group.by="orig.ident",  label.size=5, reduction='umap')


#C----
```

```
library(limma)
Malignant=read.table("Malignant cells.txt", header=T, sep="\t",
check.names=F, row.names=1)
pbmc1 = scRNA[,Malignant[,1]]
Count = as.data.frame(pbmc1@assays[["RNA"]]@counts)
meta = pbmc1@meta.data

pbmc1 <- CreateSeuratObject(counts = Count)
pbmc1@meta.data$Type = B$orig.ident

### SCT
pbmc1 <- SCTransform(pbmc1)

#PCA
pbmc1 <- RunPCA(pbmc1, features = VariableFeatures(object = pbmc1))

pbmc1 <- RunTSNE(pbmc1, dims=pc.num) %>% RunUMAP(dims=1:20)

pbmc1 <- FindNeighbors(pbmc1, dims = 1:20)
pbmc1 <- FindClusters(pbmc1, resolution = 1)

DimPlot(pbmc1, reduction = "umap")

save(pbmc1,file='Malignant.Rdata')
#D----
library(dplyr)
library(Seurat)
library(tidyverse)
library(patchwork)
library(SingleR)
library(WGCNA)
library(reshape2)
library(stringr)
library(SingleR)

load("Malignant.Rdata")
ann = read.table("ann.txt", header=T, sep="\t", check.names=F,row.names =
1)

datadf <- as.matrix(pbmc1@assays$RNA@data )
idd1 <- pbmc1@meta.data
Inter.id1<-cbind(rownames(idd1),idd1$seurat_clusters)
rownames(Inter.id1)<-rownames(idd1)
colnames(Inter.id1)<-c("CellID","Celltype")
Inter.id1<-as.data.frame(Inter.id1)
head(Inter.id1)

set.seed(9)
A = merge(scRNA_harmony@meta.data,Inter.id1,by=0)
Inter.id1 = A[,c(1,5)]
colnames(Inter.id1)<-c("CellID","Celltype")
rownames(Inter.id1) = Inter.id1[,1]

Inter1<-datadf[,Inter.id1$CellID]
Inter2<-as.matrix(Inter1)
Inter.id1$Celltype=as.factor(Inter.id1$Celltype)
Inter2[1:4,1:4]
x=Inter.id1$Celltype
pseudocell.size = 30
new_ids_list1 = list()
```

```
length(levels(Inter.id1$Celltype))

table(Inter.id1$Celltype)
for (i in 1:length(levels(Inter.id1$Celltype))) {
  cluster_id = levels(Inter.id1$Celltype)[i]
  cluster_cells <- rownames(Inter.id1[Inter.id1$Celltype == cluster_id,])
  cluster_size <- length(cluster_cells)
  pseudo_ids <- floor(seq_along(cluster_cells)/pseudocell.size)
  pseudo_ids <- paste0(cluster_id, "_Cell", pseudo_ids)
  names(pseudo_ids) <- sample(cluster_cells,)
  new_ids_list1[[i]] <- pseudo_ids
}

new_ids <- unlist(new_ids_list1)
new_ids <- as.data.frame(new_ids)
head(new_ids)
new_ids_length <- table(new_ids)
new_ids_length

new_colnames <- rownames(new_ids)

colnames(datadf)
all.data<-datadf[,as.character(new_colnames)]
all.data <- t(all.data)

new.data2<-aggregate( all.data ,
                      list(name=new_ids[,1]),FUN=mean)

rownames(new.data2)<-new.data2$name
new.data2<-new.data2[,-1]
new_ids_length<-as.matrix(new_ids_length)


new_good_ids<-as.matrix(new_ids_length)
result<-t(new.data2)[,rownames(new_good_ids)]
dim(result)


rloop = read.table("rloop.txt",sep="\t",header=T,check.names=F,row.names
= 1)


Cluster1 <- result[intersect(rownames(rloop),rownames(result)),]
dim(Cluster1)

##

type = "unsigned"
corType = "pearson"
corFnc = ifelse(corType=="pearson", cor, bicor)
corFnc
maxPOutliers = ifelse(corType=="pearson",1,0.05)

robustY = ifelse(corType=="pearson",T,F)
dataExpr  <- as.matrix(t(Cluster1))


gsg = goodSamplesGenes(dataExpr, verbose = 3)
gsg$allOK
gsg$goodSamples
```

```
if (!gsg$allOK){
  # Optionally, print the gene and sample names that were removed:
  if (sum(!gsg$goodGenes)>0)
    printFlush(paste("Removing genes:",
                     paste(names(dataExpr)[!gsg$goodGenes], collapse =
",")));
  if (sum(!gsg$goodSamples)>0)
    printFlush(paste("Removing samples:",
                     paste(rownames(dataExpr)[!gsg$goodSamples], collapse
= ",")));
  # Remove the offending genes and samples from the data:
  dataExpr = dataExpr[gsg$goodSamples, gsg$goodGenes]
}

nGenes = ncol(dataExpr)
nSamples = nrow(dataExpr)


powers = c(c(1:10), seq(from = 12, to=30, by=2))
sft = pickSoftThreshold(dataExpr, powerVector=powers,
                        networkType="signed", verbose=5)


par(mfrow = c(1,2))
cex1 = 0.9
plot(sft$fitIndices[,1], -sign(sft$fitIndices[,3])*sft$fitIndices[,2],
     xlab="Soft Threshold (power)",
     ylab="Scale Free Topology Model Fit,signed R^2",type="n",
     main = paste("Scale independence"))
text(sft$fitIndices[,1], -sign(sft$fitIndices[,3])*sft$fitIndices[,2],
     labels=powers,cex=cex1,col="red")
abline(h=0.85,col="red")

plot(sft$fitIndices[,1], sft$fitIndices[,5],
     xlab="Soft Threshold (power)",ylab="Mean Connectivity", type="n",
     main = paste("Mean connectivity"))
text(sft$fitIndices[,1], sft$fitIndices[,5], labels=powers,
     cex=cex1, col="red")


power = sft$powerEstimate
softPower  = power
softPower


cor <- WGCNA::cor

net = blockwiseModules(dataExpr, power = power, maxBlockSize =
nGenes,#nGenes
         TOMType = "unsigned", minModuleSize = 10,
         reassignThreshold = 0, mergeCutHeight = 0.25,
         numericLabels = TRUE, pamRespectsDendro = FALSE,
         saveTOMs=TRUE, corType = corType,
         maxPOutliers=maxPOutliers, loadTOMs=TRUE,
         saveTOMFileBase = paste0("dataExpr", ".tom"),
         verbose = 3)

table(net$colors)
```

```
##
# Convert labels to colors for plotting
moduleLabels = net$colors
moduleColors = labels2colors(moduleLabels)
moduleColors
plotDendroAndColors(net$dendrograms[[1]],
moduleColors[net$blockGenes[[1]]],
                    "Module colors",
                    dendroLabels = FALSE, hang = 0.03,
                    addGuide = TRUE, guideHang = 0.05)


MEs = net$MEs

MEs_col = MEs
colnames(MEs_col) = paste0("ME", labels2colors(
  as.numeric(str_replace_all(colnames(MEs),"ME",""))))
MEs_col = orderMEs(MEs_col)

head(MEs_col)

plotEigengeneNetworks(MEs, "Eigengene adjacency heatmap",
                      marDendro = c(3,3,2,4),
                      marHeatmap = c(3,4,2,2),
                      plotDendrograms = T,
                      xLabelsAngle = 90)

moduleColors <- labels2colors(net$colors)
MEList = moduleEigengenes(dataExpr, colors = moduleColors)
MEs = MEList$eigengenes
MEDiss = 1-cor(MEs);
METree = hclust(as.dist(MEDiss), method = "average");

plotEigengeneNetworks(MEs,
                      "Eigengene adjacency heatmap",
                      marHeatmap = c(3,4,2,2),
                      plotDendrograms = FALSE,
                      xLabelsAngle = 90)
table(moduleColors )

which.module="blue";
ME=MEs[, paste("ME",which.module, sep="")]
par(mfrow=c(2,1), mar=c(0,4.1,4,2.05))
plotMat(t(scale(dataExpr[,moduleColors==which.module ]) ),
        nrgcols=30,rlabels=F,rcols=which.module,
        main=which.module, cex.main=2)
par(mar=c(2,2.3,0.5,0.8))
barplot(ME, col=which.module, main="", cex.main=2,
        ylab="eigengene expression",xlab="array sample")

load(net$TOMFiles, verbose=T)

TOM <- as.matrix(TOM)
TOM[1:4,1:4]
dissTOM = 1-TOM
# Transform dissTOM with a power to make moderately strong
# connections more visible in the heatmap
plotTOM = dissTOM^7
# Set diagonal to NA for a nicer plot
```

```
diag(plotTOM) = NA
# Call the plot function
table(moduleColors)
TOMplot(plotTOM, net$dendrograms[[1]], moduleColors[net$blockGenes[[1]]],
        main = "Network heatmap plot, all genes",
        col=gplots::colorpanel(250,'red',"orange",'lemonchiffon'))

Labels = as.data.frame(moduleLabels)
Colors = as.data.frame(moduleColors)
Labels = cbind(Labels,Colors)
Colors = cbind(id=rownames(Labels),Labels)
write.table(Colors,file="moduleLabels.txt",sep="\t",quote=F,row.names=F)

##
cli = read.table("cli.txt", header=T, sep="\t", check.names=F,
row.names=1)

moduleTraitCor_noFP <- cor(MEs, cli, use = "p");
moduleTraitPvalue_noFP = corPvalueStudent(moduleTraitCor_noFP, nSamples);

pdf(file="Module-trait.pdf", width=7, height=7)
textMatrix_noFP <- paste(signif(moduleTraitCor_noFP, 2), "\n(",
signif(moduleTraitPvalue_noFP, 1), ")", sep = "");
par(mar = c(10, 8.5, 3, 3));
labeledHeatmap(Matrix = moduleTraitCor_noFP,
               xLabels = names(cli),
               yLabels = names(MEs),
               ySymbols = names(MEs),
               colorLabels = FALSE,
               colors = blueWhiteRed(50),
               textMatrix = textMatrix_noFP,
               setStdMargins = FALSE,
               cex.text = 0.65,
               zlim = c(-1,1),
               main = paste("Module-trait relationships"))
dev.off()
##

#F----
library(ggplot2)
library(ggpubr)
library(limma)
library(reshape2)
library(tidyverse)
library(ggplot2)
library(plyr)
load("Malignant.Rdata")
exp = as.data.frame(pbmc1@assays[["RNA"]]@counts)
moduleLabels=read.table("moduleLabels.txt", header=T, sep="\t",
check.names=F, row.names=1)
moduleLabels = moduleLabels[moduleLabels$moduleLabels == "9" |
moduleLabels$moduleLabels == "5" |
                              moduleLabels$moduleLabels == "10" |
moduleLabels$moduleLabels == "8",]

score <- cal_CRDscore(expr = exp, n.bins = 50, circadians =
rownames(moduleLabels), study.type = "scRNAseq")
score = as.data.frame(score)
score = cbind(id=rownames(score),score)
write.table(score,file="score.txt",sep="\t",quote=F,row.names=F)
```

```
score=read.table("score.txt", header=T, sep="\t", check.names=F,
row.names=1)
Type=ifelse(score[,"score"]<=median(score$score), "Low", "High")
score$group=Type

umap = as.data.frame(pbmc1@reductions[["umap"]]@cell.embeddings)
data = merge(umap,score,by=0)

ggplot(data = data,aes(x=UMAP_1,y=UMAP_2,color = group))+theme_classic()
+
  geom_point()+
  scale_color_manual(values = c("#fe8d5b","#63c6a6"))

pbmc1@meta.data$group=score$group
meta = pbmc1@meta.data

a <- data.frame(table(meta$seurat_clusters,meta$group))
a<- ddply(a,.(Var2),transform,percent=Freq/sum(Freq)*100)
a$label = paste0(sprintf("%.1f", a$percent), "%")

a %>%
  drop_na() %>%
  ggplot(aes(fill=Var2, y= percent, x = Var1)) +
  geom_bar(position="fill", stat = "identity") +
  scale_y_continuous(labels = scales::percent) +
  ylab("Percent(%)")+xlab("")+labs(fill="Rloop")+
  theme_gray()+
  theme(legend.position = "none",
        panel.grid.major =element_blank(),
        panel.grid.minor = element_blank(),
        panel.background = element_blank(),
        panel.border = element_blank()
  )

x = as.data.frame(table(meta$seurat_clusters))
ggplot(x, aes(x=Var1, y=Freq, group = 1)) +
  geom_line(size=2,color="#CBD5E8")+
  geom_point(size=3)+
  theme(axis.text.x = element_text(angle = 90))+
  labs(x="",y="Malignant Cell Number")+
  theme(panel.grid.major =element_blank(),
        panel.grid.minor = element_blank(),
        panel.background = element_blank(),
        panel.border = element_blank())




#G----
score=read.table("score.txt", header=T, sep="\t", check.names=F,
row.names=1)
meta=read.table("pbmc1@meta.data.txt", header=T, sep="\t", check.names=F)
cli = read.table("Cli.txt", header=T, sep="\t", check.names=F,
row.names=1)

rt = merge(meta,cli,by.x=5,by.y = 1)
```

```
rt1 = merge(score,rt,by.x=0,by.y = 2)

data = rt1[,c(2,12)]
colnames(data) = c("score","Type")


group=levels(factor(data$Type))
data$Type=factor(data$Type, levels=group)
comp=combn(group,2)
my_comparisons=list()
for(i in 1:ncol(comp)){my_comparisons[[i]]<-comp[,i]}

ggplot(data,aes(x = Type , y=score, color=Type))+
  stat_boxplot(geom="errorbar",width=0.6)+
  geom_boxplot(alpha=0.7,outlier.shape =
NA,size=0.7,width=0.7,fatten=0.7)+
  theme_bw()+
  theme(panel.grid=element_blank())+
  stat_compare_means(comparisons = my_comparisons,method =
"wilcox.test")+
  theme(legend.position = "none")+
  ggtitle("") +
  theme(axis.text.x = element_text(angle = 90, hjust = 1))+
  ylab("RloopScore")+
  theme(axis.title.x=element_blank(),
        axis.text.y = element_text(size = 15,face = "bold",color =
"black"),
        axis.title.y = element_text(size = 15,face = "bold",color =
"black"),
        axis.text.x = element_text(size = 15,face = "bold",color =
"black"),
        legend.position = "none",
        plot.title = element_text(face = "bold",size=15,hjust = 0.5))


data = rt1[,c(2,15)]
colnames(data) = c("score","Type")


group=levels(factor(data$Type))
data$Type=factor(data$Type, levels=group)
comp=combn(group,2)
my_comparisons=list()
for(i in 1:ncol(comp)){my_comparisons[[i]]<-comp[,i]}

ggplot(data,aes(x = Type , y=score, color=Type))+
  stat_boxplot(geom="errorbar",width=0.6)+
  geom_boxplot(alpha=0.7,outlier.shape =
NA,size=0.7,width=0.7,fatten=0.7)+
  theme_bw()+
  theme(panel.grid=element_blank())+
  stat_compare_means(comparisons = my_comparisons,method =
"wilcox.test")+
  theme(legend.position = "none")+
  ggtitle("") +
  theme(axis.text.x = element_text(angle = 90, hjust = 1))+
  ylab("RloopScore")+
  theme(axis.title.x=element_blank(),
        axis.text.y = element_text(size = 15,face = "bold",color =
"black"),
```

```
        axis.title.y = element_text(size = 15,face = "bold",color =
"black"),
        axis.text.x = element_text(size = 15,face = "bold",color =
"black"),
        legend.position = "none",
        plot.title = element_text(face = "bold",size=15,hjust = 0.5))

data = rt1[,c(2,16)]
colnames(data) = c("score","Type")


group=levels(factor(data$Type))
data$Type=factor(data$Type, levels=group)
comp=combn(group,2)
my_comparisons=list()
for(i in 1:ncol(comp)){my_comparisons[[i]]<-comp[,i]}

ggplot(data,aes(x = Type , y=score, color=Type))+
  stat_boxplot(geom="errorbar",width=0.6)+
  geom_boxplot(alpha=0.7,outlier.shape =
NA,size=0.7,width=0.7,fatten=0.7)+
  theme_bw()+
  theme(panel.grid=element_blank())+
  stat_compare_means(comparisons = my_comparisons,method =
"wilcox.test")+
  theme(legend.position = "none")+
  ggtitle("") +
  theme(axis.text.x = element_text(angle = 90, hjust = 1))+
  ylab("RloopScore")+
  theme(axis.title.x=element_blank(),
        axis.text.y = element_text(size = 15,face = "bold",color =
"black"),
        axis.title.y = element_text(size = 15,face = "bold",color =
"black"),
        axis.text.x = element_text(size = 15,face = "bold",color =
"black"),
        legend.position = "none",
        plot.title = element_text(face = "bold",size=15,hjust = 0.5))


data = rt1[,c(2,17)]
colnames(data) = c("score","Type")


group=levels(factor(data$Type))
data$Type=factor(data$Type, levels=group)
comp=combn(group,2)
my_comparisons=list()
for(i in 1:ncol(comp)){my_comparisons[[i]]<-comp[,i]}

ggplot(data,aes(x = Type , y=score, color=Type))+
  stat_boxplot(geom="errorbar",width=0.6)+
  geom_boxplot(alpha=0.7,outlier.shape =
NA,size=0.7,width=0.7,fatten=0.7)+
  theme_bw()+
  theme(panel.grid=element_blank())+
  stat_compare_means(comparisons = my_comparisons,method =
"wilcox.test")+
  theme(legend.position = "none")+
  ggtitle("") +
```

```
  theme(axis.text.x = element_text(angle = 90, hjust = 1))+
  ylab("RloopScore")+
  theme(axis.title.x=element_blank(),
        axis.text.y = element_text(size = 15,face = "bold",color =
"black"),
        axis.title.y = element_text(size = 15,face = "bold",color =
"black"),
        axis.text.x = element_text(size = 15,face = "bold",color =
"black"),
        legend.position = "none",
        plot.title = element_text(face = "bold",size=15,hjust = 0.5))


#H----
library(GSVA)
library(limma)
library(GSEABase)
gmtFile="kegg_pathway.gmt"

exp = as.data.frame(pbmc1@assays[["RNA"]]@data)
dimnames=list(rownames(exp),colnames(exp))
mat=matrix(as.numeric(as.matrix(exp)),nrow=nrow(exp),dimnames=dimnames)

geneSet=getGmt(gmtFile,
               geneIdType=SymbolIdentifier())

ssgseaScore=gsva(mat, geneSet, method='gsva', kcdf='Gaussian',
abs.ranking=TRUE ,min.sz = 10)

normalize=function(x){
  return((x-min(x))/(max(x)-min(x)))}

ssgseaOut=normalize(ssgseaScore)
ssgseaOut=rbind(id=colnames(ssgseaOut),ssgseaOut)

meta = read.table("pbmc1@meta.data.txt", header=T, sep="\t",
check.names=F,row.names = 1)
meta = arrange(meta,desc(group))
GSVA_hall = ssgseaOut[,rownames(meta)]
GSVA_hall = GSVA_hall[-1,]

dimnames=list(rownames(GSVA_hall),colnames(GSVA_hall))
GSVA_hall=matrix(as.numeric(as.matrix(GSVA_hall)),nrow=nrow(GSVA_hall),di
mnames=dimnames)


library(limma)

table(meta$group)
group <- factor(c(rep("High", 8398), rep("Low", 8397)), levels =
c('Tumor', 'Normal'))
design <- model.matrix(~0+group)
colnames(design) = levels(factor(group))
rownames(design) = colnames(GSVA_hall)
design

compare <- makeContrasts(High - Low, levels=design)
fit <- lmFit(GSVA_hall, design)
fit2 <- contrasts.fit(fit, compare)
fit3 <- eBayes(fit2)
```

```
dat_plot <- topTable(fit3, coef=1, number=200)


library(stringr)

dat_plot$threshold = factor(ifelse(dat_plot$t  >-2, ifelse(dat_plot$t >=
2 ,'Up','NoSignifi'),'Down'),levels=c('Up','Down','NoSignifi'))

dat_plot <- dat_plot %>% arrange(t)

dat_plot$id <- factor(dat_plot$id,levels = dat_plot$id)

library(ggplot2)
library(ggthemes)
library(ggprism)
p <- ggplot(data = dat_plot,aes(x = id,y = t,fill = threshold)) +
  geom_col()+
  coord_flip() +
  scale_fill_manual(values = c('Up'=
'#36638a','NoSignifi'='#cccccc','Down'='#7bcd7b')) +
  geom_hline(yintercept = c(-2,2),color = 'white',size =
0.5,lty='dashed') +
  xlab('') +
  ylab('t value of GSVA score') +
  guides(fill=F)+
  theme_prism(border = T) +
  theme(
    axis.text.y = element_blank(),
    axis.ticks.y = element_blank()
  )

low1 <- dat_plot %>% filter(t < -2) %>% nrow()

low0 <- dat_plot %>% filter( t < 0) %>% nrow()

high0 <- dat_plot %>% filter(t < 2) %>% nrow()

high1 <- nrow(dat_plot)


p + geom_text(data = dat_plot[1:low1,],aes(x = id,y = 0.1,label = id),
                   hjust = 0,color = 'black') +
  geom_text(data = dat_plot[(high0 +1):high1,],aes(x = id,y = -0.1,label
= id),
            hjust = 1,color = 'black')



#fig2----
#A----
load("Malignant.Rdata")
score=read.table("score.txt",sep="\t",header=T,check.names=F,row.names =
1)
score$Type = ifelse(score$score > median(score$score), "High", "Low")
score = score[colnames(pbmc1),]
pbmc1$Type = score$Type

gene = read.table("TAA.txt",sep="\t",header=T,check.names=F,row.names =
1)
```

```
P1 = DotPlot(pbmc1,features = rownames(gene),group.by = "Type" ) +
coord_flip()
rt = P1[["data"]]

ggplot(rt, aes(id,features.plot))+theme_bw()+coord_flip()+
  xlab("")+ylab(" ")+theme(axis.text.x = element_text(angle = 90, hjust =
1))+

geom_point(aes(size=pct.exp,color=avg.exp.scaled),alpha=0.6)+scale_colour
_gradient(low = "navy",high = "#ff0000")

gmtFile="kegg_pathway.gmt"
geneSet=getGmt(gmtFile,
               geneIdType=SymbolIdentifier())

exp=as.data.frame(pbmc1@assays[["RNA"]]@counts)
dimnames=list(rownames(exp),colnames(exp))
mat=matrix(as.numeric(as.matrix(exp)),nrow=nrow(exp),dimnames=dimnames)

ssgseaScore=gsva(mat, geneSet, method='ssgsea', kcdf='Gaussian',
abs.ranking=TRUE ,min.sz = 1)

normalize=function(x){
  return((x-min(x))/(max(x)-min(x)))}

ssgseaOut=normalize(ssgseaScore)

rt = merge(score,t(ssgseaOut),by=0)
rt$score = as.numeric(rt$score)

group=levels(factor(rt$Type))
rt$Type=factor(rt$Type, levels=group)
comp=combn(group,2)
my_comparisons=list()
for(i in 1:ncol(comp)){my_comparisons[[i]]<-comp[,i]}

ggplot(data=rt,aes(x = Type, y=score, color=Type))+
  stat_boxplot(geom="errorbar",width=0.5,size=1.3)+
  geom_boxplot(alpha=1,outlier.shape = NA,size=1.3,width=0.5,fatten=1)+
  theme_bw()+
  theme(panel.grid=element_blank())+

#stat_compare_means(aes(group=cancer),method="anova",symnum.args=list(cut
points = c(0, 0.001, 0.01, 0.05, 1), symbols = c("***", "**", "*",
"ns")),label = "p.signif")+
  stat_compare_means(aes(group=Type),
                     method="wilcox.test",
                     symnum.args=list(cutpoints = c(0, 0.001, 0.01, 0.05,
1), symbols = c("***", "**", "*", " ")),
                     label = "p.signif")+
  theme(legend.position = "right")+
  ggtitle("") +
  xlab("")+
  ylab("ICD score")+
  scale_color_manual(values=c("#00AFBB", "#E7B800"))+
  theme_bw()+
  theme(plot.title = element_text(hjust = 0.5),
        axis.text.x = element_text(angle = 40, hjust = 1))+
  theme(panel.grid.major =element_blank(),
        panel.grid.minor = element_blank(),
```

```
        panel.background = element_blank(),
        panel.border = element_blank())+
  theme(
    axis.title.x=element_blank(),
    axis.text.y = element_text(size = 15,face = "bold",color = "black"),
    axis.title.y = element_text(size = 15,face = "bold",color = "black"),
    legend.position = "top",

    plot.title = element_text(face = "bold",size=15,hjust = 0.5))
```

```
#B----
load("scRNA.Rdata")
moduleLabels=read.table("moduleLabels.txt", header=T, sep="\t",
check.names=F, row.names=1)
moduleLabels = moduleLabels[moduleLabels$moduleLabels == "9" |
moduleLabels$moduleLabels == "5" |
                              moduleLabels$moduleLabels == "10" |
moduleLabels$moduleLabels == "8",]
exp = as.data.frame(scRNA@assays[["RNA"]]@counts)
score <- cal_CRDscore(expr = exp, n.bins = 50, circadians =
rownames(moduleLabels), study.type = "scRNAseq")
score = as.data.frame(score)

A = scRNA@meta.data
B = as.data.frame(table(A$orig.ident))
score$id = A$orig.ident

id = c()
exp = c()
for (i in B[,1]) {
  rt = score[score$id == i,]
  sum = log2(sum(rt$score))
  id = c(id,i)
  exp = c(exp,sum)
}

data = data.frame(id,exp)
data$Type = ifelse(data$exp > median(data$exp),"High","Low")

l = data[data$Type == "Low",]
h = data[data$Type == "High",]
Low = B[which( B$orig.ident %in% l[,1] ),]
Low$Type = "Low"
High = B[which( B$orig.ident %in% h[,1] ),]
High$Type = "High"
A1 = rbind(High,Low)
A2 = A1

A2$singleR1[which(A2$singleR1 == 'B_cell')] <- 'immune cell'
A2$singleR1[which(A2$singleR1 == 'DC')] <- 'immune cell'
A2$singleR1[which(A2$singleR1 == 'Endothelial_cells')] <- 'Other'
A2$singleR1[which(A2$singleR1 == 'Epithelial_cells')] <- 'Other'
```

```
A2$singleR1[which(A2$singleR1 == 'Fibroblasts')] <- 'Other'
A2$singleR1[which(A2$singleR1 == 'Macrophage')] <- 'immune cell'
A2$singleR1[which(A2$singleR1 == 'Mast')] <- 'immune cell'
A2$singleR1[which(A2$singleR1 == 'NK_cell')] <- 'immune cell'
A2$singleR1[which(A2$singleR1 == 'Oligodendrocytes')] <- 'Other'
A2$singleR1[which(A2$singleR1 == 'T_cells')] <- 'immune cell'


Low = A2[A2$Type == "Low",]
High = A2[A2$Type == "High",]
diamonds_df <- Low %>% group_by(Type, singleR1) %>% tally() %>%
  mutate(prop = round((n/sum(n)) * 100 , digits = 2)) %>% ungroup()

ggplot(diamonds_df, aes(x = Type, y = prop, fill = singleR1)) +
  geom_col(color = "black") +
  geom_text(aes(label = paste0(prop, "%")), position =
position_stack(vjust = 0.5), size = 4) +
  scale_fill_brewer(palette = "Set3") +
  coord_polar("y", start = 0)  +
  facet_wrap(~Type) +
  theme_void() +
  theme(strip.text = element_text(size = 16),
        legend.position = "top")

diamonds_df <- High %>% group_by(Type, singleR1) %>% tally() %>%
  mutate(prop = round((n/sum(n)) * 100 , digits = 2)) %>% ungroup()

ggplot(diamonds_df, aes(x = Type, y = prop, fill = singleR1)) +
  geom_col(color = "black") +
  geom_text(aes(label = paste0(prop, "%")), position =
position_stack(vjust = 0.5), size = 4) +
  scale_fill_brewer(palette = "Set3") +
  coord_polar("y", start = 0)  +
  facet_wrap(~Type) +
  theme_void() +
  theme(strip.text = element_text(size = 16),
        legend.position = "top")


#C----
library(plyr)
load("Tcell.Rdata")
meat=read.table("meat.txt", header=T, sep="\t", check.names=F,
row.names=1)
meta.data = scRNA@meta.data
meat1 = meat[rownames(meta.data),]
meta.data$Type = meat1$Type

a <- data.frame(table(meta.data$Type,meta.data$singleR1))
a<- ddply(a,.(Var1),transform,percent=Freq/sum(Freq)*100)
a$label = paste0(sprintf("%.1f", a$percent), "%")


a %>%
  drop_na() %>%
  ggplot(aes(fill=Var2, y= percent, x = Var1)) +
  geom_bar(position="fill", stat = "identity") +
  scale_fill_manual(values = c("#8DD3C7" ,"#FFFFB3" , "#FB8072"
,"#BEBADA","#80B1D3" ,"#FDB462" ,"#B3DE69",
                              "#FCCDE5", "#BC80BD","#D9D9D9"))+
```

```
  scale_y_continuous(labels = scales::percent) +
  ylab("Percent(%)")+xlab("")+labs(fill="T cell")+
  theme_gray()+
  theme(
    panel.grid.major =element_blank(),
    panel.grid.minor = element_blank(),
    panel.background = element_blank(),
    panel.border = element_blank()
  )



load("scRNA.Rdata")

celltype = read.table("celltype.txt", header=T, sep="\t", check.names=F)
clusters <- scRNA@meta.data$seurat_clusters
scRNA@meta.data$singleR1=celltype[match(clusters,celltype$ClusterID),'cel
ltype']

A = scRNA@meta.data
B = as.data.frame(table(A$orig.ident))
B1 = as.data.frame(table(A$singleR1))

m=3
for (i in B[,1]) {
  x = A[A$orig.ident == i,]
  k=1
  for (j in B1[,1]) {
    sum = sum(x$singleR1 == j)/nrow(x)
    B1[k,m] = sum
    k = k+1
  }
  m = m+1
}
A4 = B1
A4 = A4[,-2]
rownames(A4) = A4[,1]
A4 = A4[,-1]
colnames(A4) = B[,1]

data = t(A4)
Type = read.table("Rloopscore.txt", header=T, sep="\t",
check.names=F,row.names = 1)
rt = merge(Type,data,by.x=0,by.y=0)

rt3 = rt[,c(3,13)]
colnames(rt3)=c("Type","Expression")
rt3$Expression = rt3$Expression * 100

group=levels(factor(rt3$Type))
rt3$Type=factor(rt3$Type, levels=group)
comp=combn(group,2)
my_comparisons=list()
for(i in 1:ncol(comp)){my_comparisons[[i]]<-comp[,i]}



p=ggplot(rt3, aes(x=Type, y=Expression,fill=Type)) +
  geom_violin(trim=FALSE,color="white") +
  geom_boxplot(width=0.2,position=position_dodge(0.9))+
```

```
  geom_point(aes(x=Type,
y=Expression),pch=19,position=position_dodge(0.9),size=0.5)+
  scale_fill_manual(values = c("#eaeae8","#7bacb0"))+
  theme_bw()+
  stat_compare_means(comparisons = my_comparisons,method="wilcox.test")+
  theme(
    legend.position = "none",
    panel.border = element_blank(),axis.line = element_line(colour =
"black",size=1),
    panel.grid.major = element_blank(),
    panel.grid.minor = element_blank())+
  ggtitle("")+
  ylab("T cell")+xlab("")
p
pdf(file="T cell.pdf",width=2.5,height=3.5)
plot(p)
dev.off()




#D----
library(GSVA)
library(GSEABase)
load("scRNA.Rdata")

celltype = read.table("celltype.txt", header=T, sep="\t", check.names=F)
clusters <- scRNA@meta.data$seurat_clusters
scRNA@meta.data$singleR1=celltype[match(clusters,celltype$ClusterID),'cel
ltype']

meta.data = scRNA@meta.data

meta.data = meta.data[meta.data$singleR1 == "NK_cell",]
B = as.data.frame(table(meta.data$orig.ident))
scRNA = scRNA[,rownames(meta.data)]

geneSets <- getGmt('T cell.gmt')
exp = as.data.frame(scRNA@assays[["SCT"]]@data)
dimnames=list(rownames(exp),colnames(exp))
mat=matrix(as.numeric(as.matrix(exp)),nrow=nrow(exp),dimnames=dimnames)


GSVA_hall <- gsva(expr= mat,
                  gset.idx.list=geneSets,
                  mx.diff=T,
                  method = "ssgsea",
                  kcdf="Gaussian",
                  parallel.sz=1)

hall = t(ssgseaOut)
hall = as.data.frame(hall)
hall = merge(meta.data[1],hall,by=0)


Type = read.table("Rloopscore.txt", header=T, sep="\t",
check.names=F,row.names = 1)
rt = merge(Type,t(hall),by=0)
```

```
rt3 = rt[,c(3,8)]
group=levels(factor(rt3$Type))
rt3$Type=factor(rt3$Type, levels=group)
comp=combn(group,2)
my_comparisons=list()
for(i in 1:ncol(comp)){my_comparisons[[i]]<-comp[,i]}
colnames(rt3)

ggplot(rt3, aes(x=Type, y=`NK & T-cell exhaustion`,fill=Type)) +
  geom_violin(trim=FALSE,color="white") +
  geom_boxplot(width=0.2,position=position_dodge(0.9))+
  geom_point(aes(x=Type, y=`NK & T-cell
exhaustion`),pch=19,position=position_dodge(0.9),size=0.5)+
  scale_fill_manual(values = c("#eaeae8","#7bacb0"))+
  theme_bw()+
  stat_compare_means(comparisons = my_comparisons,method="t.test")+
  theme(
    legend.position = "none",
    panel.border = element_blank(),axis.line = element_line(colour =
"black",size=1),
    panel.grid.major = element_blank(),
    panel.grid.minor = element_blank())+
  ggtitle("")+
  ylab("NK exhaustion Score")+xlab("")
```

```
#E----
setwd("GSE146100")
library(Seurat)
library(msigdbr)
library(GSVA)
library(tidyverse)
library(clusterProfiler)
library(patchwork)
library(limma)
library(devtools)

rt = read.table("GSE146100_NormData.txt", header=T, sep="\t",
check.names=F, row.names=1)
scRNA = CreateSeuratObject(rt, min.cells = 3, project = "W1",min.features
=300)

### SCT
scRNA <- SCTransform(scRNA)

### PCA
scRNA <- RunPCA(scRNA, npcs=50, verbose=FALSE)

pc.num=1:30
scRNA <- RunTSNE(scRNA, dims=pc.num) %>% RunUMAP( dims=pc.num)

scRNA <- FindNeighbors(scRNA, dims = pc.num)
```

```
scRNA <- FindClusters(scRNA, resolution = 0.5)

refdata <-  HumanPrimaryCellAtlasData()

testdata <- GetAssayData(scRNA, slot="data")

clusters <- scRNA@meta.data$seurat_clusters

cellpred <- SingleR(test = testdata, ref = refdata, labels = 
refdata$label.main,
                    method = "cluster", clusters = clusters,
                    assay.type.test = "logcounts", assay.type.ref = 
"logcounts")

celltype = data.frame(ClusterID=rownames(cellpred), 
celltype=cellpred$labels, stringsAsFactors = FALSE)
scRNA@meta.data$singleR1=celltype[match(clusters,celltype$ClusterID),'cel
ltype']
DimPlot(scRNA, group.by="singleR1", label=F, label.size=5, 
reduction='tsne')
DimPlot(scRNA, group.by="orig.ident", label=F, label.size=5, 
reduction='tsne')


#FGH----
A = scRNA@meta.data
table(A$singleR)
A = A[A$singleR == "Fibroblasts" | A$singleR == "Epithelial_cells" | 
A$singleR == "Endothelial_cells",]
B = scRNA[,rownames(A)]
B = as.data.frame(B@assays[["RNA"]]@counts)
write.table(B, file="raw_counts_matrix.txt", sep="\t", quote=F, col.names 
= NA)

ann = A
ann = ann[10]
ann = cbind(id=rownames(ann),ann)
write.table(ann, file="annotations_file.txt", sep="\t", quote=F, 
row.names=F,col.names = F)

library(infercnv)

infercnv_obj = 
CreateInfercnvObject(raw_counts_matrix="raw_counts_matrix.txt",

annotations_file="annotations_file.txt",
                                    delim="\t",

gene_order_file="gene_order_file.txt",

ref_group_names=c("Fibroblasts","Endothelial_cells"))


infercnv_obj = infercnv::run(infercnv_obj,
                             cutoff=0.1,  # use 1 for smart-seq, 0.1 for 
10x-genomics
                             out_dir="CNV",  # dir is auto-created for 
storing outputs
                             cluster_by_groups=T,   # cluster
                             denoise=T,
```

```
                              HMM=F
)


infercnv_obj = readRDS("./run.final.infercnv_obj")
expr <- infercnv_obj@expr.data
test_loc <- infercnv_obj@observation_grouped_cell_indices
test_loc <- test_loc$Epithelial_cells

anno.df=data.frame(
  CB=c(colnames(expr)[test_loc]),
  class=c(rep("Epithelial_cells",length(test_loc)))
)
head(anno.df)

gn <- rownames(expr)
geneFile <- read.table("gene_order_file.txt",header = F,sep =
"\t",stringsAsFactors = F)
rownames(geneFile)=geneFile$V1
sub_geneFile <-  geneFile[intersect(gn,geneFile$V1),]
expr=expr[intersect(gn,geneFile$V1),]
head(sub_geneFile,4)
expr[1:4,1:4]

set.seed(20221207)
kmeans.result <- kmeans(t(expr), 3)
kmeans_df <- data.frame(kmeans_class=kmeans.result$cluster)
kmeans_df$CB=rownames(kmeans_df)
kmeans_df=kmeans_df%>%inner_join(anno.df,by="CB")
kmeans_df_s=arrange(kmeans_df,kmeans_class)
rownames(kmeans_df_s)=kmeans_df_s$CB
kmeans_df_s$CB=NULL
kmeans_df_s$kmeans_class=as.factor(kmeans_df_s$kmeans_class)
head(kmeans_df_s)


expr2=expr-1
expr2=expr2 ^ 2
CNV_score=as.data.frame(colMeans(expr2))
colnames(CNV_score)="CNV_score"
CNV_score$CB=rownames(CNV_score)
kmeans_df_s$CB=rownames(kmeans_df_s)
CNV_score=CNV_score%>%inner_join(kmeans_df_s,by="CB")

color_v=RColorBrewer::brewer.pal(8, "Dark2")[1:15]
CNV_score%>%ggplot(aes(kmeans_class,CNV_score))+geom_violin(aes(fill=kmea
ns_class),color="NA")+
  scale_fill_manual(values = color_v)+
  theme_bw()

table(CNV_score$kmeans_class)
kmeans_df_s = kmeans_df_s[,-3]

write.table(kmeans_df_s, file = "146100Malignant.txt", quote = FALSE, sep
= '\t', row.names = T, col.names = NA)



rt = read.table("146100Malignant.txt", header=T, sep="\t", check.names=F,
row.names=1)
```

```
rt$kmeans_class = ifelse(rt$kmeans_class != "2", "Malignant", "Epithelial
cells")

meta.data = scRNA@meta.data
meta.data = meta.data[A$singleR1 == "Epithelial_cells",]
scRNA1 = scRNA[,rownames(meta.data)]
meta = scRNA1@meta.data
rt = rt[rownames(meta),]
meta$Type = rt$class
scRNA1@meta.data$Type = rt$kmeans_class

rt1 = rt[rt$kmeans_class == "Malignant",]
scRNA1 = scRNA[,rownames(rt1)]
exp = as.matrix(scRNA1@assays$RNA@data )

scRNA1 = CreateSeuratObject(exp, min.cells = 3,min.features =300)

### SCT
scRNA1 <- SCTransform(scRNA1)

### PCA
scRNA1 <- RunPCA(scRNA1, npcs=50, verbose=FALSE)

pc.num=1:30
scRNA1 <- RunTSNE(scRNA1, dims=pc.num) %>% RunUMAP( dims=pc.num)

scRNA1 <- FindNeighbors(scRNA1, dims = pc.num)
scRNA1 <- FindClusters(scRNA1, resolution = 0.2)


moduleLabels=read.table("moduleLabels.txt", header=T, sep="\t",
check.names=F, row.names=1)
moduleLabels = moduleLabels[moduleLabels$moduleLabels == "9" |
moduleLabels$moduleLabels == "5" |
                              moduleLabels$moduleLabels == "10" |
moduleLabels$moduleLabels == "8",]

exp = as.data.frame(scRNA1@assays[["RNA"]]@counts)
score <- cal_CRDscore(expr = exp, n.bins = 50, circadians =
rownames(moduleLabels), study.type = "scRNAseq")
score = as.data.frame(score)
score$Type = ifelse(score$score > median(score$score),"High","Low")

score = score[colnames(scRNA1),]
scRNA1@meta.data$Type = CRDscore$Type

DimPlot(scRNA1, group.by="Treat", label=T, label.size=5,
reduction='umap')
DimPlot(scRNA1, group.by="Type", label=T, label.size=5, reduction='tsne')

save(scRNA1,file='Maligent.Rdata')








meat = scRNA@meta.data
rt = merge(score,meat,by=0)
```

```
rt1 = rt[,c(2,13)]
colnames(rt1)[2] = "Type"
rt1$Type <-
factor(rt1$Type,levels=c("Maligent","Endothelial_cells","Fibroblasts","Ep
ithelial_cells","immune cell"))


group=levels(factor(rt1$Type))
rt1$Type=factor(rt1$Type, levels=group)
comp=combn(group,2)
my_comparisons=list()
for(i in 1:ncol(comp)){my_comparisons[[i]]<-comp[,i]}

ggplot(data=rt1,aes(x = Type, y=score, color=Type))+
  stat_boxplot(geom="errorbar",width=0.5,size=1.3)+
  geom_boxplot(alpha=1,outlier.shape = NA,size=1.3,width=0.5,fatten=1)+
  theme_bw()+
  theme(panel.grid=element_blank())+
  stat_compare_means(comparisons = my_comparisons,method="t.test")+
  theme(legend.position = "right")+
  ggtitle("") +
  xlab("")+
  ylab("Rloopscore")+
  rotate_x_text(60)+
  theme(
    axis.title.x=element_blank(),
    axis.text.y = element_text(size = 15,face = "bold",color = "black"),
    axis.title.y = element_text(size = 15,face = "bold",color = "black"),
    axis.text.x = element_text(size = 15,face = "bold",color = "black"),
    legend.position = "none",
    plot.title = element_text(face = "bold",size=15,hjust = 0.5))




meta.data = scRNA1@meta.data
Rloop = read.table("score.txt", header=T, sep="\t", check.names=F,
row.names=1)
rt = merge(Rloop,meta.data,by=0)

rt1 = rt[,c(2,16)]
colnames(rt1)[2] = "Type"

group=levels(factor(rt1$Type))
rt1$Type=factor(rt1$Type, levels=group)
comp=combn(group,2)
my_comparisons=list()
for(i in 1:ncol(comp)){my_comparisons[[i]]<-comp[,i]}

ggplot(data=rt1,aes(x = Type, y=CRDscore, color=Type))+
  stat_boxplot(geom="errorbar",width=0.5,size=1.3)+
  geom_boxplot(alpha=1,outlier.shape = NA,size=1.3,width=0.5,fatten=1)+
  theme_bw()+
  theme(panel.grid=element_blank())+
  stat_compare_means(comparisons = my_comparisons,method="t.test")+
  theme(legend.position = "right")+
  ggtitle("") +
  xlab("")+
```

```
  ylab("Rloopscore")+
  rotate_x_text(60)+
  theme(
    axis.title.x=element_blank(),
    axis.text.y = element_text(size = 15,face = "bold",color = "black"),
    axis.title.y = element_text(size = 15,face = "bold",color = "black"),
    axis.text.x = element_text(size = 15,face = "bold",color = "black"),
    legend.position = "none",
    plot.title = element_text(face = "bold",size=15,hjust = 0.5))


#fig3----
#A-F----
load("scRNA.Rdata")
meta = scRNA@meta.data
T_cell = meta[meta$singleR1 == "T_cells",]
T_cell = scRNA[,rownames(T_cell)]
DimPlot(T_cell,label.size=5, reduction='umap')

exp = as.data.frame(T_cell@assays[["RNA"]]@counts)

pbmc1 <- CreateSeuratObject(counts = exp, project = "T cell")

pbmc1 <- NormalizeData(pbmc1, normalization.method = "LogNormalize",
scale.factor = 10000)

pbmc1 <- FindVariableFeatures(pbmc1, selection.method = "vst", nfeatures
= 2000)

#Scaling the data
all.genes <- rownames(pbmc1)
pbmc1 <- ScaleData(pbmc1, features = all.genes)

#PCA
pbmc1 <- RunPCA(pbmc1, features = VariableFeatures(object = pbmc1))

pbmc1 <- FindNeighbors(pbmc1, dims = 1:20)
pbmc1 <- FindClusters(pbmc1, resolution = 1)
pbmc1 <- RunTSNE(pbmc1, dims = 1:20)
pbmc1 <- RunUMAP(pbmc1, dims = 1:20)


DimPlot(pbmc1, reduction = "umap",label = T)
DimPlot(pbmc1, reduction = "tsne",label = T)

select_genes <- c("CTLA4","PDCD1","TIQIT","CXCL13")
select_genes <- c("GZMB","GNLY","NKG7")
select_genes <- c("STAT6","GATA3")
select_genes <- c("IL12RB2","IFNG","STAT4")
select_genes <- c("NR4A2","CREM","IRF4")
select_genes <- c("CCR7","LEF1","SELL","TCF7")
select_genes <- c("IKZF2","FOXP3","IL2RA")
select_genes <- c("NKG7","IFNG","GZMK","GZMB","PRF1","GNLY","GZMA","IL2")
select_genes <- c("LAG3","LAYN","CTLA4","PDCD1")
DotPlot(pbmc1, features = select_genes )+ coord_flip()


celltype = read.table("T_cell.txt", header=T, sep="\t", check.names=F)
clusters <- pbmc1@meta.data$seurat_clusters
```

```
pbmc1@meta.data$singleR1=celltype[match(clusters,celltype$ClusterID),'cel
ltype']

DimPlot(pbmc1, group.by="singleR1",  label.size=5, reduction='umap')
DimPlot(pbmc1, reduction = "umap",label = T)

save(pbmc1,file='Tcell.Rdata')



meat=read.table("pbmc1@meta.data.txt", header=T, sep="\t",
check.names=F,row.names = 1)

library(Rcpp)
Rcpp::sourceCpp(code='
#include <Rcpp.h>
using namespace Rcpp;
// [[Rcpp::export]]
IntegerMatrix asMatrix(NumericVector rp,
                       NumericVector cp,
                       NumericVector z,
                       int nrows,
                       int ncols){
  int k = z.size() ;
  IntegerMatrix  mat(nrows, ncols);
  for (int i = 0; i < k; i++){
      mat(rp[i],cp[i]) = z[i];
  }
  return mat;
}' )

as_matrix <- function(mat){
  row_pos <- mat@i
  col_pos <- findInterval(seq(mat@x)-1,mat@p[-1])
  tmp <- asMatrix(rp = row_pos, cp = col_pos, z = mat@x,
                  nrows =  mat@Dim[1], ncols = mat@Dim[2])
  row.names(tmp) <- mat@Dimnames[[1]]
  colnames(tmp) <- mat@Dimnames[[2]]
  return(tmp)
}

exp = as_matrix(scRNA@assays[["RNA"]]@counts)

High = meat[meat$group == "High",]
Low = meat[meat$group == "Low",]

High = exp[,rownames(High)]
Low = exp[,rownames(Low)]

load("Tcell.Rdata")
Tmeat = scRNA@meta.data
Tcell = exp[,rownames(Tmeat)]

load("Mac.Rdata")
Macmeat = scRNA@meta.data
Mac = exp[,rownames(Macmeat)]

high = meat[meat$group == "High",]
high = high[8]
low = meat[meat$group == "Low",]
```

```
low = low[8]

Tmeat = Tmeat[7]
colnames(Tmeat)="group"
Macmeat1 = Macmeat[7]
colnames(Macmeat1)="group"
Macmeat2 = Macmeat[8]
colnames(Macmeat2)="group"

meat1 = rbind(high,Tmeat,Macmeat1)
meat1 = cbind(rownames(meat1),meat1)
same = intersect(rownames(meat1),colnames(exp))
meat1 = meat1[same,]
write.table(meat1, file="meat_High.txt", quote=F, sep="\t",row.names = F)

exp1 = exp[,rownames(meat1)]
exp1 = cbind(rownames(exp1),exp1)
write.table(exp1, file="count_High.txt", quote=F, sep="\t",row.names = F)

meat1 = rbind(low,Tmeat,Macmeat1)
meat1 = cbind(rownames(meat1),meat1)
same = intersect(rownames(meat1),colnames(exp))
meat1 = meat1[same,]
write.table(meat1, file="meat_Low.txt", quote=F, sep="\t",row.names = F)

exp1 = exp[,rownames(meat1)]
exp1 = cbind(rownames(exp1),exp1)
write.table(exp1, file="count_Low.txt", quote=F, sep="\t",row.names = F)


#cellphonedb method statistical_analysis meat_Low.txt count_Low.txt --
iterations=1000 --threads=8 --counts-data=gene_name --output-path=Low

chemokines <- grep("^CXC|CCL|CCR|CX3|XCL|XCR",
mymeans$interacting_pair,value = T)
costimulatory <-
grep("CD86|CD80|CD48|LILRB2|LILRB4|TNF|CD2|ICAM|SLAM|LT[AB]|NECTIN2|CD40|
CD70|CD27|CD28|CD58|TSLP|PVR|CD44|CD55|ICOS|CD[1-9]",
                      mymeans$interacting_pair,value = T)
coinhibitory <- grep("SIRP|CD47|TIGIT|CTLA4|PDCD1|CD274|LAG3|HAVCR|VSIR",
                     mymeans$interacting_pair,value = T)

#chemokines
library(ggplot2)
library(reshape2)
library(dplyr)
mypvals=read.table("High//pvalues.txt", header=T, sep="\t",
check.names=F, row.names=1)
mymeans=read.table("High/means.txt", header=T, sep="\t", check.names=F,
row.names=1)


mymeans %>% dplyr::filter(interacting_pair %in% chemokines)%>%
  dplyr::select("interacting_pair",starts_with("High"),ends_with("High"))
%>%
  reshape2::melt() -> meansdf

colnames(meansdf)<- c("interacting_pair","CC","means")

mypvals %>% dplyr::filter(interacting_pair %in% chemokines)%>%
```

```
dplyr::select("interacting_pair",starts_with("High"),ends_with("High"))%>
%
  reshape2::melt()-> pvalsdf

colnames(pvalsdf)<- c("interacting_pair","CC","pvals")
pvalsdf$joinlab<- paste0(pvalsdf$interacting_pair,"_",pvalsdf$CC)
meansdf$joinlab<- paste0(meansdf$interacting_pair,"_",meansdf$CC)
pldf <- merge(pvalsdf,meansdf,by = "joinlab")


pldf%>% filter(pvals < 0.05) ->A
A$Type = "Rloop_High"

ann = mymeans[,1:10]
A = merge(ann,A,by.x = 1,by.y = 2)
A = A[,-c(2:6,9:10)]
A = A[,-c(7:8)]


mypvals=read.table("Low/pvalues.txt", header=T, sep="\t", check.names=F,
row.names=1)
mymeans=read.table("Low/means.txt", header=T, sep="\t", check.names=F,
row.names=1)
mymeans %>% dplyr::filter(interacting_pair %in% chemokines)%>%
  dplyr::select("interacting_pair",starts_with("Low"),ends_with("Low"))
%>%
  reshape2::melt() -> meansdf

colnames(meansdf)<- c("interacting_pair","CC","means")

mypvals %>% dplyr::filter(interacting_pair %in% chemokines)%>%

dplyr::select("interacting_pair",starts_with("Low"),ends_with("Low"))%>%
  reshape2::melt()-> pvalsdf

colnames(pvalsdf)<- c("interacting_pair","CC","pvals")
pvalsdf$joinlab<- paste0(pvalsdf$interacting_pair,"_",pvalsdf$CC)
meansdf$joinlab<- paste0(meansdf$interacting_pair,"_",meansdf$CC)
pldf <- merge(pvalsdf,meansdf,by = "joinlab")


pldf%>% filter(pvals < 0.05) ->B
B$Type = "Rloop_Low"

ann = mymeans[,1:10]
B = merge(ann,B,by.x = 1,by.y = 2)
B = B[,-c(2:6,9:10)]
B = B[,-c(7:8)]

C = rbind(A,B)
write.table(C,file="chemokines.txt",sep="\t",quote=F,row.names=F)

E=read.table("chemokines.txt", header=T, sep="\t", check.names=F)
ggplot(E, aes(CC.x,interacting_pair)) +  facet_grid(. ~ Type)+
  xlab("")+ylab(" ")+theme(axis.text.x = element_text(angle = 90, hjust =
1))+
  geom_point(aes(size=-log10(pvals+0.0001),color=means),alpha=0.6)+
```

```
  scale_colour_gradientn(colours =
c('blue','cyan','white','orange','red',midpoint =
median(E$means)))+theme_bw()



#costimulatory
mypvals=read.table("High/pvalues.txt", header=T, sep="\t", check.names=F,
row.names=1)
mymeans=read.table("High/means.txt", header=T, sep="\t", check.names=F,
row.names=1)

mymeans %>% dplyr::filter(interacting_pair %in% costimulatory)%>%
  dplyr::select("interacting_pair",starts_with("High"),ends_with("High"))
%>%
  reshape2::melt() -> meansdf

colnames(meansdf)<- c("interacting_pair","CC","means")

mypvals %>% dplyr::filter(interacting_pair %in% costimulatory)%>%

dplyr::select("interacting_pair",starts_with("High"),ends_with("High"))%>
%
  reshape2::melt()-> pvalsdf

colnames(pvalsdf)<- c("interacting_pair","CC","pvals")
pvalsdf$joinlab<- paste0(pvalsdf$interacting_pair,"_",pvalsdf$CC)
meansdf$joinlab<- paste0(meansdf$interacting_pair,"_",meansdf$CC)
pldf <- merge(pvalsdf,meansdf,by = "joinlab")


pldf%>% filter(pvals < 0.05) ->A
A$Type = "Rloop_High"

ann = mymeans[,1:10]
A = merge(ann,A,by.x = 1,by.y = 2)
A = A[,-c(2:6,9:10)]
A = A[,-c(7:8)]


mypvals=read.table("Low/pvalues.txt", header=T, sep="\t", check.names=F,
row.names=1)
mymeans=read.table("Low/means.txt", header=T, sep="\t", check.names=F,
row.names=1)
mymeans %>% dplyr::filter(interacting_pair %in% costimulatory)%>%
  dplyr::select("interacting_pair",starts_with("Low"),ends_with("Low"))
%>%
  reshape2::melt() -> meansdf

colnames(meansdf)<- c("interacting_pair","CC","means")

mypvals %>% dplyr::filter(interacting_pair %in% costimulatory)%>%

dplyr::select("interacting_pair",starts_with("Low"),ends_with("Low"))%>%
  reshape2::melt()-> pvalsdf

colnames(pvalsdf)<- c("interacting_pair","CC","pvals")
pvalsdf$joinlab<- paste0(pvalsdf$interacting_pair,"_",pvalsdf$CC)
meansdf$joinlab<- paste0(meansdf$interacting_pair,"_",meansdf$CC)
pldf <- merge(pvalsdf,meansdf,by = "joinlab")
```

```
pldf%>% filter(pvals < 0.05) ->B
B$Type = "Rloop_Low"

ann = mymeans[,1:10]
B = merge(ann,B,by.x = 1,by.y = 2)
B = B[,-c(2:6,9:10)]
B = B[,-c(7:8)]

C = rbind(A,B)
write.table(C,file="costimulatory.txt",sep="\t",quote=F,row.names=F)

E=read.table("costimulatory.txt", header=T, sep="\t", check.names=F)

ggplot(E, aes(CC.x,interacting_pair)) +  facet_grid(. ~ Type)+
  xlab("")+ylab(" ")+theme(axis.text.x = element_text(angle = 90, hjust =
1))+
  geom_point(aes(size=-log10(pvals+0.0001),color=means),alpha=0.6)+
  scale_colour_gradientn(colours =
c('blue','cyan','white','orange','red',midpoint = 0.868))+theme_bw()


#coinhibitory
mypvals=read.table("High/pvalues.txt", header=T, sep="\t", check.names=F,
row.names=1)
mymeans=read.table("High/means.txt", header=T, sep="\t", check.names=F,
row.names=1)


mymeans %>% dplyr::filter(interacting_pair %in% coinhibitory)%>%
  dplyr::select("interacting_pair",starts_with("High"),ends_with("High"))
%>%
  reshape2::melt() -> meansdf

colnames(meansdf)<- c("interacting_pair","CC","means")

mypvals %>% dplyr::filter(interacting_pair %in% coinhibitory)%>%

dplyr::select("interacting_pair",starts_with("High"),ends_with("High"))%>
%
  reshape2::melt()-> pvalsdf

colnames(pvalsdf)<- c("interacting_pair","CC","pvals")
pvalsdf$joinlab<- paste0(pvalsdf$interacting_pair,"_",pvalsdf$CC)
meansdf$joinlab<- paste0(meansdf$interacting_pair,"_",meansdf$CC)
pldf <- merge(pvalsdf,meansdf,by = "joinlab")


pldf%>% filter(pvals < 0.05) ->A
A$Type = "Rloop_High"

ann = mymeans[,1:10]
A = merge(ann,A,by.x = 1,by.y = 2)
A = A[,-c(2:6,9:10)]
A = A[,-c(7:8)]


mypvals=read.table("Low/pvalues.txt", header=T, sep="\t", check.names=F,
row.names=1)
```

```
mymeans=read.table("Low/means.txt", header=T, sep="\t", check.names=F,
row.names=1)
mymeans %>% dplyr::filter(interacting_pair %in% coinhibitory)%>%
  dplyr::select("interacting_pair",starts_with("Low"),ends_with("Low"))
%>%
  reshape2::melt() -> meansdf

colnames(meansdf)<- c("interacting_pair","CC","means")

mypvals %>% dplyr::filter(interacting_pair %in% coinhibitory)%>%

dplyr::select("interacting_pair",starts_with("Low"),ends_with("Low"))%>%
  reshape2::melt()-> pvalsdf

colnames(pvalsdf)<- c("interacting_pair","CC","pvals")
pvalsdf$joinlab<- paste0(pvalsdf$interacting_pair,"_",pvalsdf$CC)
meansdf$joinlab<- paste0(meansdf$interacting_pair,"_",meansdf$CC)
pldf <- merge(pvalsdf,meansdf,by = "joinlab")


pldf%>% filter(pvals < 0.05) ->B
B$Type = "Rloop_Low"

ann = mymeans[,1:10]
B = merge(ann,B,by.x = 1,by.y = 2)
B = B[,-c(2:6,9:10)]
B = B[,-c(7:8)]

C = rbind(A,B)
write.table(C,file="coinhibitory.txt",sep="\t",quote=F,row.names=F)

E=read.table("coinhibitory.txt", header=T, sep="\t", check.names=F)
ggplot(E, aes(CC.x,interacting_pair)) +  facet_grid(. ~ Type)+
  xlab("")+ylab(" ")+theme(axis.text.x = element_text(angle = 90, hjust =
1))+
  geom_point(aes(size=-log10(pvals+0.0001),color=means),alpha=0.6)+
  scale_colour_gradientn(colours =
c('blue','cyan','white','orange','red',midpoint =
median(E$means)))+theme_bw()





#I----

LUAD = read.table("LUAD.TPM.txt", header=T, sep="\t",
check.names=F,row.names = 1)
LUAD = log2(LUAD+1)
LUAD = cbind(id = rownames(LUAD),LUAD)
write.table(LUAD, file="LUAD.xlsx", quote=F, sep="\t",row.names = F )

load("Malignant.Rdata")
meta = read.table("pbmc1@meta.data.txt", header=T, sep="\t",
check.names=F,row.names = 1)
exp = as.data.frame(pbmc1@assays[["RNA"]]@data)

meta1 = meta[meta$group == "High",]
High = exp[,rownames(meta1)]
```

```
High = cbind(id = rownames(High),High)
write.table(High, file="High.xlsx", quote=F, sep="\t",row.names = F )


meta2 = meta[meta$group == "Low",]
Low = exp[,rownames(meta2)]
Low = cbind(id = rownames(Low),Low)
write.table(Low, file="Low.xlsx", quote=F, sep="\t",row.names = F )

#python CytoSig_run.py -i Low.xlsx -o output

score_h = fread("High.Zscore")
score_l = fread("Low.Zscore")
score = cbind(score_h,score_l[,-1])
score = as.data.frame(score)
rownames(score) = score[,1]

score = as.data.frame(t(score))
score = score[-1,]
score = t(score)

for (i in 1:ncol(score)) {
  score[,i] = as.numeric(score[,i])
}
score$group = A$group




exp = score
pval=c()
name=c()
tval=c()
logFC=c()
m6a_sym=names(exp)[1:(ncol(exp)-1)]

k=1
for(gene in m6a_sym){
  print(k)
  k=k+1

  p=t.test(exp[,gene]~exp$group)$p.value
  t=t.test(exp[,gene]~exp$group)[["statistic"]][["t"]]
  logFC1=log2(t.test(exp[,gene]~exp$group)[["estimate"]][["mean in group
High"]])-log2(t.test(exp[,gene]~exp$group)[["estimate"]][["mean in group
Low"]])

  pval=c(pval,p)
  name=c(name,gene)
  tval=c(tval,t)
  logFC=c(logFC,logFC1)
}
out = data.frame(name,pval,tval,logFC)
write.table(out, file="Malignant cell_t
value.txt",sep="\t",quote=F,row.names = F)

LUAD = fread("LUAD.Zscore",data.table = F)
rownames(LUAD) = LUAD[,1]
LUAD = LUAD[,-1]
```

```
svg = c()
gene =c()
j=1
for (i in rownames(LUAD)) {
  g1 = i
  gene = c(gene,g1)
  g2 = sum(LUAD[i,])/ncol(LUAD)
  svg = c(svg,g2)
  print(j)
  j=j+1
}
data = data.frame(gene,svg)
write.table(data, file="LUAD_Cytokine activity.txt", sep="\t", quote=F,
row.names = F)


load("scRNA.Rdata")

A = scRNA@meta.data
T_cell = A[A$singleR == "T_cells",]
Macrophage = A[A$singleR == "Macrophage",]
me = rbind(T_cell,Macrophage)

markers_T <- FindMarkers(object = scRNA, ident.1 = "T_cells", min.pct =
0.25,group.by = "singleR",test.use = "t")
markers_T = cbind(id = rownames(markers_T), markers_T)
write.table(markers_T, file="T_Maker.txt", sep="\t", quote=F,
row.names=F)

markers_M <- FindMarkers(object = scRNA, ident.1 = "Macrophage", min.pct
= 0.25,group.by = "singleR",test.use = "t")
markers_M = cbind(id = rownames(markers_M), markers_M)
write.table(markers_M, file="Macrophage_Maker.txt", sep="\t", quote=F,
row.names=F)


LUAD = read.table("LUAD.TPM.txt", header=T, sep="\t",
check.names=F,row.names = 1)
LUAD = log2(LUAD+1)
TT = read.table("T_Maker.txt", header=T, sep="\t",
check.names=F,row.names = 1)
MM = read.table("Macrophage_Maker.txt", header=T, sep="\t",
check.names=F,row.names = 1)
t = read.table("Malignant cell_t value.txt", header=T, sep="\t",
check.names=F)
t = t[t$tval >= 0,]

same = intersect(rownames(LUAD),t[,1])
LUAD_Cyto = LUAD[same,]
LUAD_T = LUAD[rownames(TT),]
LUAD_M = LUAD[rownames(MM),]

gene_name1<-c()
gene_name2<-c()
cor_r<-c()
pvalue<-c()
k=1
for (i in rownames(LUAD_Cyto)) {
  print(k)
  k=k+1
```

```
  for (j in 1:nrow(LUAD_T)) {

    A = LUAD_Cyto[i,]
    B = LUAD_T[j,]
    g1=i
    g2=rownames(LUAD_T)[j]
    c_r=cor(as.numeric(B),as.numeric(A),method="pearson")
    p=cor.test(as.numeric(B),as.numeric(A),method ="pearson")[[3]]
    gene_name1=c(gene_name1,g1)
    gene_name2=c(gene_name2,g2)
    cor_r=c(cor_r,c_r)
    pvalue=c(pvalue,p)
  }
}

data_cor<-data.frame(gene_name1,gene_name2,cor_r,pvalue)
write.table(data_cor, file="cor_LUAD-T.txt", quote=F, sep="\t",row.names
= F )



gene_name1<-c()
gene_name2<-c()
cor_r<-c()
pvalue<-c()
k=1
for (i in rownames(LUAD_Cyto)) {
  print(k)
  k=k+1
  for (j in 1:nrow(LUAD_M)) {

    A = LUAD_Cyto[i,]
    B = LUAD_M[j,]
    g1=i
    g2=rownames(LUAD_M)[j]
    c_r=cor(as.numeric(B),as.numeric(A),method="pearson")
    p=cor.test(as.numeric(B),as.numeric(A),method ="pearson")[[3]]
    gene_name1=c(gene_name1,g1)
    gene_name2=c(gene_name2,g2)
    cor_r=c(cor_r,c_r)
    pvalue=c(pvalue,p)
  }
}

data_cor<-data.frame(gene_name1,gene_name2,cor_r,pvalue)
write.table(data_cor, file="cor_LUAD-M.txt", quote=F, sep="\t",row.names
= F )

library(tidyverse)

library(hrbrthemes)
library(circlize)
library(kableExtra)
library(viridis)
library(igraph)
library(ggraph)
library(colormap)
connect=read.table("all1.txt", header=T, sep="\t", check.names=F)

c( as.character(connect$from), as.character(connect$to)) %>%
```

```
  as_tibble() %>%
  group_by(value) %>%
  dplyr::summarize(n=n()) -> coauth
colnames(coauth) <- c( "name" , "n" )

connect <- connect %>%
  filter(from %in% coauth$name) %>%
  filter(to %in% coauth$name)

# Add label angle
number_of_bar=nrow(coauth)
coauth$id = seq(1, nrow(coauth))
angle= 360 * (coauth$id-0.5) /number_of_bar     # I substract 0.5 because
the letter must have the angle of the center of the bars. Not extreme
right(1) or extreme left (0)
coauth$hjust <- ifelse(angle > 90 & angle<270, 1, 0)
coauth$angle <- ifelse(angle > 90 & angle<270, angle+180, angle)

# Create a graph object with igraph
mygraph <- graph_from_data_frame( connect, vertices = coauth, directed =
FALSE )

# prepare a vector of n color in the viridis scale
mycolor <- colormap(colormap=colormaps$viridis, nshades=max(coauth$grp))
mycolor <- sample(mycolor, length(mycolor))

# Make the graph
ggraph(mygraph, layout="circle") +
  geom_edge_link(edge_colour="black", edge_alpha=0.2, edge_width=0.3,
fold=FALSE) +
  geom_node_point(aes(size=n, color=as.factor(grp), fill=grp), alpha=0.9)
+
  scale_size_continuous(range=c(0.5,8)) +
  scale_color_manual(values=mycolor) +
  geom_node_text(aes(label=paste("    ",name,"    "), angle=angle,
hjust=hjust), size=2.3, color="black") +
  theme_void() +
  theme(
    plot.margin=unit(c(0,0,0,0), "null"),
    panel.spacing=unit(c(0,0,0,0), "null")
  ) +
  expand_limits(x = c(-1.2, 1.2), y = c(-1.2, 1.2))



ggraph(mygraph, layout="circle") +
  geom_edge_link(aes(edge_colour=as.factor(Type)), edge_alpha=0.5,
edge_width=0.6, fold=FALSE) +
  geom_node_point(aes(size=n, color=as.factor(grp), fill=grp), alpha=0.9)
+
  scale_size_continuous(range=c(0.5,8)) +
  scale_color_manual(values=mycolor) +
  geom_node_text(aes(label=paste("    ",name,"    "), angle=angle,
hjust=hjust), size=2.3, color="black") +
  theme_void() +
  theme(
    plot.margin=unit(c(0,0,0,0), "null"),
    panel.spacing=unit(c(0,0,0,0), "null")
  ) +
  expand_limits(x = c(-1.2, 1.2), y = c(-1.2 , 1.2))
```

```
rt=read.table("Malignant cell_t value.txt", header=T, sep="\t",
check.names=F)
rt = rt[rt$tval >= 0,]

rt$tval = log2(rt$tval+1)
rt$col = "A"

ggplot(data=rt, aes(x=reorder(name, tval), y=tval,fill=col)) +
  scale_fill_manual(values = "#379b98")+
  geom_bar(stat="identity")+
  labs(y="Normlized t value",x="")+
  theme(legend.position = "none")+
  theme(panel.grid.major =element_blank(),
        panel.grid.minor = element_blank(),
        panel.background = element_blank(),
        panel.border = element_blank())+
  theme(
    axis.text.y = element_text(size = 15,face = "bold",color = "black"),
    axis.title.y = element_text(size = 15,face = "bold",color = "black"),
    axis.text.x = element_text(angle = 90, hjust = 1,size = 15,face =
"bold",color = "black"))

rt1=read.table("LUAD_Cytokine activity.txt", header=T, sep="\t",
check.names=F)
rownames(rt1) = rt1[,1]
rt1$col = "A"


ggplot(data=rt1, aes(x=gene1, y=svg,fill=col)) +
  scale_fill_manual(values = "#379b98")+
  geom_bar(stat="identity")+
  labs(y="Cytokine activity",x="Cytokines in LUAD patiens")+
  theme(legend.position = "none")+
  theme(panel.grid.major =element_blank(),
        panel.grid.minor = element_blank(),
        panel.background = element_blank(),
        panel.border = element_blank())+
  theme(
    axis.text.y = element_text(size = 15,face = "bold",color = "black"),
    axis.title.x = element_text(size = 15,face = "bold",color = "black"),
    axis.title.y = element_text(size = 15,face = "bold",color = "black"),
    axis.text.x = element_text(angle = 90, hjust = 1,size = 15,face =
"bold",color = "black"))


load("scRNA.Rdata")
meta = scRNA@meta.data
TT = meta[meta$singleR == "T_cells",]
scRNA_T = scRNA[,rownames(TT)]
gene=read.table("T_Maker.txt", header=T, sep="\t", check.names=F)
scRNA_T = scRNA_T[gene[,1],]

exp = as.data.frame(scRNA_T@assays[["RNA"]]@counts)
A = rownames(exp)
ensembls <- mapIds(org.Hs.eg.db, keys = A, keytype = "SYMBOL",
column="ENSEMBL")
rownames(exp) = ensembls
exp1 <- count2tpm(countMat = exp,idType = "Ensembl", source = "default")
```

```
exp2 = t(exp1)
write.table(exp2, file="expT.txt", sep="\t", quote=F)

data=read.table("expT1.txt", header=T, sep="\t", check.names=F)
data$col = "A"
ggplot(data=data, aes(x=reorder(gene, svg), y=svg,fill=col)) +
  scale_fill_manual(values = "#561a65")+
  geom_bar(stat="identity")+
  labs(y="log2(TPM)",x="")+
  theme(legend.position = "none")+
  theme(panel.grid.major =element_blank(),
        panel.grid.minor = element_blank(),
        panel.background = element_blank(),
        panel.border = element_blank())+
  theme(
    axis.text.y = element_text(size = 15,face = "bold",color = "black"),
    axis.title.y = element_text(size = 15,face = "bold",color = "black"),
    axis.text.x = element_text(angle = 90, hjust = 1,
                               size = 15,face = "bold",color = "black"))+
  coord_flip()



load("scRNA.Rdata")
meta = scRNA@meta.data
TT = meta[meta$singleR == "Macrophage",]
scRNA_T = scRNA[,rownames(TT)]
gene=read.table("Macrophage_Maker.txt", header=T, sep="\t",
check.names=F)
scRNA_T = scRNA_T[gene[,1],]

exp = as.data.frame(scRNA_T@assays[["RNA"]]@counts)
library(org.Hs.eg.db)
A = rownames(exp)
ensembls <- mapIds(org.Hs.eg.db, keys = A, keytype = "SYMBOL",
column="ENSEMBL")
rownames(exp) = ensembls
exp1 <- count2tpm(countMat = exp,idType = "Ensembl", source = "default")
exp2 = t(exp1)
write.table(exp2, file="expM.txt", sep="\t", quote=F)

data=read.table("expM1.txt", header=T, sep="\t", check.names=F)
data$col = "A"
ggplot(data=data, aes(x=reorder(gene, svg), y=svg,fill=col)) +
  scale_fill_manual(values = "#561a65")+
  geom_bar(stat="identity")+
  labs(y="log2(TPM)",x="")+
  theme(legend.position = "none")+
  theme(panel.grid.major =element_blank(),
        panel.grid.minor = element_blank(),
        panel.background = element_blank(),
        panel.border = element_blank())+
  theme(
    axis.text.y = element_text(size = 15,face = "bold",color = "black"),
    axis.title.y = element_text(size = 15,face = "bold",color = "black"),
    axis.text.x = element_text(angle = 90, hjust = 1,
                               size = 15,face = "bold",color = "black"))+
  coord_flip()

#J----
```

```
celltype=read.table("celltype.txt", header=T, sep="\t", check.names=F)
clusters <- scRNA@meta.data$seurat_clusters
scRNA@meta.data$singleR1 =
celltype[match(clusters,celltype$ClusterID),'celltype']

Malignant=read.table("Malignant cell_t value.txt", header=T, sep="\t",
check.names=F)
Cyto=read.table("pbmc1@meta.data.txt", header=T, sep="\t", check.names=F)

Malignant = Malignant[Malignant$tval >= 0,]
same = intersect(Malignant[,1],rownames(scRNA))

meta = scRNA@meta.data
meta = meta[meta$singleR == "T_cells" | meta$singleR == "Macrophage",]
Cyto = Cyto[,c(1,9)]
meta = cbind(id= rownames(meta),meta)
meta1 = meta[,c(1,10)]
colnames(meta1)[2] = "group"
put = rbind(Cyto,meta1)
write.table(put, file="putB.txt", quote=F, sep="\t",row.names = F )

putB=read.table("putB.txt", header=T, sep="\t", check.names=F)
pbmc = scRNA[,putB[,1]]
pbmc@meta.data$Type = putB$group

P1 = DotPlot(pbmc,features = same,group.by = "Type" ) + coord_flip()
rt = P1[["data"]]
rt$features.plot <-
factor(rt$features.plot,levels=c("WNT3A","TGFB3","LIF","EGF","VEGFA","GDF
11","CXCL12","IL6","HGF","FGF2"))

ggplot(rt,aes(x=id , y=features.plot ,color=avg.exp.scaled,size=10)) +
  geom_point() + theme_bw() +
  scale_colour_gradientn(colours =
c('#482878','#26828e','#6dcd59','#b4de2c','#fde725')) +
  labs(x="",y="",title="",
       colour="Cytokine co-regulation") +
  theme(plot.title = element_text(hjust = 0.5))+
  theme(panel.grid.major =element_blank(),
        panel.grid.minor = element_blank(),
        panel.background = element_blank(),
        panel.border = element_blank())



#fig4----
#A----
samples=list.files("./")
samples
dir <- file.path('./',samples)
names(dir) <- samples
names(dir)=str_split(names(dir),'.txt',simplify = T)[,1]
names(dir)

scRNAlist <- list()
for(i in 1:length(dir)){
  A = fread(dir[i],data.table = F)
  rownames(A) = A[,1]
  scRNAlist[[i]] <- CreateSeuratObject(A, min.cells = 3, project =
names(dir)[i],min.features =300)
```

```
}

for (i in 1:length(scRNAlist)) {
  scRNAlist[[i]] <- NormalizeData(scRNAlist[[i]])
  scRNAlist[[i]] <- FindVariableFeatures(scRNAlist[[i]], selection.method
= "vst",nfeatures = 3000)
}


scRNA_merge <- merge(scRNAlist[[1]], y=c(scRNAlist[[2]],
scRNAlist[[3]],scRNAlist[[4]],scRNAlist[[5]],

scRNAlist[[6]],scRNAlist[[7]],scRNAlist[[8]],scRNAlist[[9]],

scRNAlist[[10]],scRNAlist[[11]],scRNAlist[[12]],scRNAlist[[13]],

scRNAlist[[14]],scRNAlist[[15]],scRNAlist[[16]],scRNAlist[[17]],

scRNAlist[[18]],scRNAlist[[19]],scRNAlist[[20]],scRNAlist[[21]],

scRNAlist[[22]],scRNAlist[[23]]))


scRNA_merge[["percent.mt"]] <- PercentageFeatureSet(scRNA_merge, pattern
= "^MT-")

scRNA <- SCTransform(scRNA_merge)

### PCA
scRNA <- RunPCA(scRNA, npcs=50, verbose=FALSE)


scRNA <- RunHarmony(scRNA, group.by.vars="orig.ident", max.iter.harmony =
20,assay.use = "SCT")

pc.num=1:30
scRNA <- RunTSNE(scRNA, reduction="harmony", dims=pc.num) %>%
RunUMAP(reduction="harmony", dims=pc.num)

scRNA <- FindNeighbors(scRNA, dims = pc.num)
scRNA <- FindClusters(scRNA, resolution = 1.2)

save(scRNA,file='scRNA.Rdata')


library(SingleR)

refdata <-  HumanPrimaryCellAtlasData()

testdata <- GetAssayData(scRNA, slot="data")

clusters <- scRNA@meta.data$seurat_clusters

cellpred <- SingleR(test = testdata, ref = refdata, labels =
refdata$label.main,
                    method = "cluster", clusters = clusters,
                    assay.type.test = "logcounts", assay.type.ref =
"logcounts")
```

```
celltype = data.frame(ClusterID=rownames(cellpred),
celltype=cellpred$labels, stringsAsFactors = FALSE)

write.csv(celltype,"celltype_singleR.csv",row.names = FALSE)


scRNA@meta.data$singleR=celltype[match(clusters,celltype$ClusterID),'cell
type']

DimPlot(scRNA, reduction = "tsne",group.by = "singleR",label = T)

#B----
exp = as.data.frame(scRNA@assays[["RNA"]]@counts)
exp = cbind(gene = rownames(exp),exp)
write.table(exp, file="raw_counts_matrix.txt", sep="\t", quote=F,
row.names=F)

ann = scRNA@meta.data
ann = ann[10]
ann = cbind(id=rownames(ann),ann)
write.table(ann, file="annotations_file.txt", sep="\t", quote=F,
row.names=F,col.names = F)

infercnv_obj =
CreateInfercnvObject(raw_counts_matrix="raw_counts_matrix.txt",

annotations_file="annotations_file.txt",
                                    delim="\t",

gene_order_file="gene_order_file.txt",
                                    ref_group_names=NULL)

# perform infercnv operations to reveal cnv signal
infercnv_obj = infercnv::run(infercnv_obj,
                             cutoff=0.1,  # use 1 for smart-seq, 0.1 for
10x-genomics
                             out_dir="output",  # dir is auto-created for
storing outputs
                             cluster_by_groups=T,   # cluster
                             denoise=T,
                             HMM=T
)


infercnv_obj = readRDS("./output_dir/run.final.infercnv_obj")
expr <- infercnv_obj@expr.data
normal_loc <- infercnv_obj@reference_grouped_cell_indices
normal_loc <- normal_loc$Fibroblasts
test_loc <- infercnv_obj@observation_grouped_cell_indices
test_loc <- test_loc$Epithelial

anno.df=data.frame(
  CB=c(colnames(expr)[normal_loc],colnames(expr)[test_loc]),
  class=c(rep("normal",length(normal_loc)),rep("test",length(test_loc)))
)
head(anno.df)

gn <- rownames(expr)
geneFile <- read.table("gene_order_file.txt",header = F,sep =
"\t",stringsAsFactors = F)
```

```
rownames(geneFile)=geneFile$V1
sub_geneFile <-  geneFile[intersect(gn,geneFile$V1),]
expr=expr[intersect(gn,geneFile$V1),]
head(sub_geneFile,4)
expr[1:4,1:4]


set.seed(20221917)
kmeans.result <- kmeans(t(expr), 7)
kmeans_df <- data.frame(kmeans_class=kmeans.result$cluster)
kmeans_df$CB=rownames(kmeans_df)
kmeans_df=kmeans_df%>%inner_join(anno.df,by="CB")
kmeans_df_s=arrange(kmeans_df,kmeans_class)
rownames(kmeans_df_s)=kmeans_df_s$CB
kmeans_df_s$CB=NULL
kmeans_df_s$kmeans_class=as.factor(kmeans_df_s$kmeans_class)
head(kmeans_df_s)

write.table(kmeans_df_s, file = "kmeans_df_s.txt", quote = FALSE, sep =
'\t', row.names = T, col.names = T)


expr2=expr-1
expr2=expr2 ^ 2
CNV_score=as.data.frame(colMeans(expr2))
colnames(CNV_score)="CNV_score"
CNV_score$CB=rownames(CNV_score)
kmeans_df_s$CB=rownames(kmeans_df_s)
CNV_score=CNV_score%>%inner_join(kmeans_df_s,by="CB")

write.table(CNV_score, file = "CNV_score.txt", quote = FALSE, sep = '\t',
row.names = F, col.names = T)



annotations <- read.table("annotations_file.txt",header = F,sep =
"\t",stringsAsFactors = F)
cluster <- read.table("kmeans_df_s.txt",header = T,sep =
"\t",stringsAsFactors = F)

annotations1 = annotations[annotations$V2 == "Epithelial",]
rownames(annotations1) = annotations1[,1]
rownames(cluster) = cluster[,1]

same = intersect(rownames(cluster),rownames(annotations1))
cluster1 = cluster[same,]
table(cluster1$kmeans_class)
cluster2 = cluster1[cluster1$kmeans_class == "2" | cluster1$kmeans_class
== "3" | cluster1$kmeans_class == "4" | cluster1$kmeans_class == "5" |
cluster1$kmeans_class == "7" ,]

write.table(cluster2, file = "Maligant.txt", quote = FALSE, sep = '\t',
row.names = F)


meat = annotations1[rownames(cluster2),]
meatB = annotations1[-which( annotations1$V1 %in% rownames(cluster2) ),]
meat$type = "Maligant"
meatB$type = "Epithelial"
meat = rbind(meat,meatB)
```

```
write.table(meat, file = "Epithelial_zhushi.txt", quote = FALSE, sep = 
'\t', row.names = F)


gene=read.table("es.txt", header=T, sep="\t", check.names=F, row.names=1)
A = as.data.frame(pbmc@assays[["RNA"]]@data)
exp = as.data.frame(scRNA@assays[["RNA"]]@counts)

rt = exp[rownames(gene),]



sample<-c()
Score<-c()
for (i in 1:ncol(rt)) {
  g1 = colnames(rt)[i]
  g2 = sum(rt[,i])/14
  sample = c(sample,g1)
  Score=c(Score,g2)
}
data<-data.frame(sample,Score)

data1 = data
data1$Score = (data1$Score-min(data1$Score))/(max(data1$Score)-
min(data1$Score))

rt = merge(data1,meat,by.x=1,by.y=1)
rt = rt[,c(2,12)]
x=colnames(rt)[1]
y=colnames(rt)[2]
colnames(rt)=c("Expression","cancer")

group=levels(factor(rt$cancer))
rt$cancer=factor(rt$cancer, levels=group)
comp=combn(group,2)
my_comparisons=list()
for(i in 1:ncol(comp)){my_comparisons[[i]]<-comp[,i]}


library(ggpubr)
library(limma)
library(reshape2)
library(tidyverse)
library(ggplot2)
ggplot(data=rt,aes(x =reorder(cancer,Expression,  FUN = median),
y=Expression, color=cancer))+
  stat_boxplot(geom="errorbar",width=0.5,size=1.3)+
  geom_boxplot(alpha=1,outlier.shape = NA,size=1.3,width=0.5,fatten=1)+
  theme_bw()+
  theme(panel.grid=element_blank())+
  stat_compare_means(comparisons = 
my_comparisons,symnum.args=list(cutpoints = c(0, 0.001, 0.01, 0.05, 1),
symbols = c("***", "**", "*", "ns")),label = "p.signif")+
  theme(legend.position = "right")+
  ggtitle("") +
  xlab("")+
  ylab("Epithelial Score")+
  scale_x_continuous(limits = c(0, 0.2))+
  rotate_x_text(60)+
  theme(
```

```
    axis.title.x=element_blank(),
    axis.text.y = element_text(size = 15,face = "bold",color = "black"),
    axis.title.y = element_text(size = 15,face = "bold",color = "black"),
    axis.text.x = element_text(size = 15,face = "bold",color = "black"),
    legend.position = "none",
    plot.title = element_text(face = "bold",size=15,hjust = 0.5))



a <- data.frame(table(cli1$`Treatement Timepoint`,cli1$singleR1))
a<- ddply(a,.(Var1),transform,percent=Freq/sum(Freq)*100)
a$label = paste0(sprintf("%.1f", a$percent), "%")

a %>%
  drop_na() %>%
  ggplot(aes(fill=Var2, y= percent, x = Var1)) +
  geom_bar(position="fill", stat = "identity") +
  scale_y_continuous(labels = scales::percent) +
  ylab("Percent(%)")+xlab("")+labs(fill="Rloop")+
  theme_gray()+
  scale_fill_manual(values= c("#FFFFB3" ,"#FDB462" ,"#80B1D3" ,
"#FB8072", "#B3DE69","#BC80BD","#8DD3C7"))+
  coord_flip()+
  theme(
    panel.grid.major =element_blank(),
    panel.grid.minor = element_blank(),
    panel.background = element_blank(),
    panel.border = element_blank()
  )


exp = as.data.frame(scRNA@assays[["RNA"]]@data)
moduleLabels=read.table("moduleLabels.txt", header=T, sep="\t",
check.names=F, row.names=1)
moduleLabels = moduleLabels[moduleLabels$moduleLabels == "9" |
moduleLabels$moduleLabels == "5" |
                              moduleLabels$moduleLabels == "10" |
moduleLabels$moduleLabels == "8",]

CRDscore <- cal_CRDscore(expr = exp, n.bins = 50, circadians =
rownames(moduleLabels), study.type = "scRNAseq")
E = as.data.frame(CRDscore)
E = cbind(id=rownames(E),E)

E$CRDscore = (E$CRDscore-min(E$CRDscore))/(max(E$CRDscore)-
min(E$CRDscore))
E1 = merge(cli1,E,by=1)

rt = E1[,c(18,6)]
x=colnames(rt)[1]
y=colnames(rt)[2]
colnames(rt)=c("Expression","cancer")

group=levels(factor(rt$cancer))
rt$cancer=factor(rt$cancer, levels=group)
comp=combn(group,2)
my_comparisons=list()
for(i in 1:ncol(comp)){my_comparisons[[i]]<-comp[,i]}
```

```
ggplot(data=rt,aes(y =reorder(cancer,Expression,  FUN = median),
x=Expression, color=cancer))+
  stat_boxplot(geom="errorbar",width=0.5,size=1.3)+
  geom_boxplot(alpha=1,outlier.shape = NA,size=1.3,width=0.5,fatten=1)+
  theme_bw()+
  theme(panel.grid=element_blank())+
  stat_compare_means(comparisons =
my_comparisons,symnum.args=list(cutpoints = c(0, 0.001, 0.01, 0.05, 1),
symbols = c("***", "**", "*", "ns")),label = "p.signif")+
  theme(legend.position = "right")+
  ggtitle("") +
  xlab("RloopScore")+
  ylab("Cell Type")+
  rotate_x_text(60)+
  theme(
    axis.title.x=element_blank(),
    axis.text.y = element_text(size = 15,face = "bold",color = "black"),
    axis.title.y = element_text(size = 15,face = "bold",color = "black"),
    axis.text.x = element_text(size = 15,face = "bold",color = "black"),
    legend.position = "none",
    plot.title = element_text(face = "bold",size=15,hjust = 0.5))


#C----
DimPlot(Maligant_scRNA, reduction = "tsne",group.by = "Treatment",cols =
c("#90a7d6","#f59268","#65c1a4"))
DimPlot(Immune_scRNA, reduction = "tsne",group.by = "Celltype",cols =
c("#90a7d6","#f59268","#65c1a4"))


#D----
meat = scRNA@meat.data
meat1 = meat[meat$singleR1 == "Maligant",]
scRNA = scRNA[,rownames(meat1)]
exp = as.data.frame(scRNA@assays$RNA@data)
scRNA1 = CreateSeuratObject(exp ,min.cells = 3,project="Maligant",
min.features = 300)

scRNA1 <- NormalizeData(scRNA1, normalization.method = "LogNormalize",
scale.factor = 10000)
scRNA1 <- FindVariableFeatures(scRNA1, selection.method = "vst",
nfeatures = 3000)
scale.genes <-  rownames(scRNA1)
scRNA1 <- ScaleData(scRNA1, features = scale.genes)
scRNA1 <- RunPCA(scRNA1, features = VariableFeatures(scRNA1))
pc.num=1:20
scRNA1 <- FindNeighbors(scRNA1, dims = pc.num)
scRNA1 <- FindClusters(scRNA1, resolution = 0.8)


scRNA1 = RunTSNE(scRNA1, dims = pc.num)
scRNA1 <- RunUMAP(scRNA1, dims = pc.num)

B = scRNA1@meta.data
Maligant = meat[meat$singleR1=="Maligant",]
Maligant = Maligant[rownames(B),]
Maligant$Type = ifelse(Maligant$CRDscore <= median(Maligant$CRDscore),
"Low", "High")
colnames(Maligant)
```

```
B$Treatement = Maligant$`Treatement Timepoint`
B$Type = Maligant$Type

scRNA1@meta.data$Treatement = B$Treatement
scRNA1@meta.data$Type = B$Type

scRNA1@meta.data$Treatement[which(scRNA1@meta.data$Treatement =='TN')] <-
'TN/RD'
scRNA1@meta.data$Treatement[which(scRNA1@meta.data$Treatement =='RD')] <-
'TN/RD'



DimPlot(scRNA1, reduction = "umap",cols =
c("grey","red"),group.by="Type")
DimPlot(scRNA1, reduction = "umap",cols =
c("red","grey"),group.by="Treatement")


saveRDS(scRNA1, file="Maligant_0.8.rds")





#E----
load("scRNA.Rdata")
meat = scRNA@meta.data
cli = read.table("cli.txt", header=T, sep="\t", check.names=F,
row.names=1)
cli = cli[rownames(meat),]

meat$Isolate = cli$Isolate
exp = as.data.frame(scRNA@assays[["RNA"]]@data)

score = read.table("score.txt", header=T, sep="\t", check.names=F,
row.names=1)
score = cbind(id=rownames(score),score)

B = as.data.frame(table(cli$Isolate))

id = c()
CRDscore = c()
for (i in B[,1]) {
  C = cli[cli$Isolate == i,]
  D = score[rownames(C),]
  g1 = i
  g2 = sum(D$CRDscore)/nrow(D)
  id = c(id,g1)
  CRDscore = c(CRDscore,g2)
}
data = data.frame(id,CRDscore)

or = read.table("or.txt", header=T, sep="\t", check.names=F, row.names=1)
rt = merge(or,data,by=0)

var="CRDscore"
rt1=rt[,c("futime","fustat",var)]
rt1$futime = rt1$futime/30
```

```
res.cut=surv_cutpoint(rt1, time="futime", event="fustat",
variables=c("CRDscore"))
cutoff=as.numeric(res.cut$cutpoint[1])
print(cutoff)
Type=ifelse(rt1[,"CRDscore"]<=cutoff, "Low", "High")
rt1$group=Type


diff=survdiff(Surv(futime, fustat) ~group,data = rt1)
pValue=1-pchisq(diff$chisq,df=1)
if(pValue<0.001){
  pValue="p<0.001"
}else{
  pValue=paste0("p=",sprintf("%.03f",pValue))
}
fit <- survfit(Surv(futime, fustat) ~ group, data = rt1)

ggsurvplot(
  fit,
  pval = TRUE,
  pval.method = TRUE,
  pval.size=5,#
  legend.title='Rloopscore', #
  legend.labs=c('High','Low'), #
  linetype = "strata",
  xlab="Time(Month)",
  ylab="Overall Response",
  break.time.by = 5,
  surv.median.line = "hv",
  ggtheme = theme_bw(),
  palette = c("#E31A1C","#1F78B4")
)


#F----
load("scRNA.Rdata")
setwd("D:\\LY\\5.3\\work\\Lung\\5.26\\fig2\\tu\\E")
cli = read.table("cli.txt", header=T, sep="\t", check.names=F,
row.names=1)
cli = cli[cli$singleR1 == "Maligant",]

moduleLabels=read.table("moduleLabels.txt", header=T, sep="\t",
check.names=F, row.names=1)
moduleLabels = moduleLabels[moduleLabels$moduleLabels == "9" |
moduleLabels$moduleLabels == "5" |
                              moduleLabels$moduleLabels == "10" |
moduleLabels$moduleLabels == "8",]


exp = as.data.frame(scRNA@assays[["RNA"]]@counts)

CRDscore <- cal_CRDscore(expr = exp, n.bins = 50, circadians =
rownames(moduleLabels), study.type = "scRNAseq")
CRDscore = as.data.frame(CRDscore)
CRDscore = cbind(id=rownames(CRDscore),CRDscore)

tab = as.data.frame(table(cli$Isolate))

id = c()
score = c()
```

```
for (i in tab[,1]) {
  rt = cli[cli$Isolate == i,]
  rtt = as.data.frame(CRDscore[rownames(rt),])
  g1 = i
  g2 = sum(rtt$CRDscore)/nrow(rtt)
  id = c(id,g1)
  score = c(score,g2)
}
data = data.frame(id,score)

rt1 = merge(data,cli,by.x=1,by.y=0)
rt = rt1[,c(8,2)]
colnames(rt)=c("cancer","Expression")

group=levels(factor(rt$cancer))
rt$cancer=factor(rt$cancer, levels=group)
comp=combn(group,2)
my_comparisons=list()
for(i in 1:ncol(comp)){my_comparisons[[i]]<-comp[,i]}
my_comparisons = my_comparisons[1]


ggplot(data=rt,aes(x =cancer, y=Expression, color=cancer))+
  stat_boxplot(geom="errorbar",width=0.5,size=1.3)+
  geom_boxplot(alpha=1,outlier.shape = NA,size=1.3,width=0.5,fatten=1)+
  theme_bw()+
  theme(panel.grid=element_blank())+
  stat_compare_means(comparisons =
my_comparisons,method="wilcox.test",symnum.args=list(cutpoints = c(0,
0.001, 0.01, 0.05, 1), symbols = c("***", "**", "*", "ns")),label =
"p.signif")+
  theme(legend.position = "right")+
  ggtitle("") +
  xlab("")+
  ylab("Rloopscore")+
  rotate_x_text(60)+
  theme(
    axis.title.x=element_blank(),
    axis.text.y = element_text(size = 15,face = "bold",color = "black"),
    axis.title.y = element_text(size = 15,face = "bold",color = "black"),
    axis.text.x = element_text(size = 15,face = "bold",color = "black"),
    legend.position = "none",
    plot.title = element_text(face = "bold",size=15,hjust = 0.5))




rt = rt1[,c(7,2)]
rt = na.omit(rt)
colnames(rt)=c("cancer","Expression")

group=levels(factor(rt$cancer))
rt$cancer=factor(rt$cancer, levels=group)
comp=combn(group,2)
my_comparisons=list()
for(i in 1:ncol(comp)){my_comparisons[[i]]<-comp[,i]}

ggplot(data=rt,aes(x =cancer, y=Expression, color=cancer))+
  stat_boxplot(geom="errorbar",width=0.5,size=1.3)+
```

```
  geom_boxplot(alpha=1,outlier.shape = NA,size=1.3,width=0.5,fatten=1)+
  theme_bw()+
  theme(panel.grid=element_blank())+
  stat_compare_means(comparisons =
my_comparisons,method="t.test",symnum.args=list(cutpoints = c(0, 0.001,
0.01, 0.05, 1), symbols = c("***", "**", "*", "ns")),label = "p.signif")+
  theme(legend.position = "right")+
  ggtitle("") +
  xlab("")+
  ylab("Rloopscore")+
  rotate_x_text(60)+
  theme(
    axis.title.x=element_blank(),
    axis.text.y = element_text(size = 15,face = "bold",color = "black"),
    axis.title.y = element_text(size = 15,face = "bold",color = "black"),
    axis.text.x = element_text(size = 15,face = "bold",color = "black"),
    legend.position = "none",

    plot.title = element_text(face = "bold",size=15,hjust = 0.5))




rt = rt1[,c(6,2)]
colnames(rt)=c("cancer","Expression")
table(rt$cancer)
rt$cancer[which(rt$cancer =='IA')] <- 'I'
rt$cancer[which(rt$cancer =='IIIB')] <- 'III'
rt$cancer[which(rt$cancer =='IIIA')] <- 'III'

group=levels(factor(rt$cancer))
rt$cancer=factor(rt$cancer, levels=group)
comp=combn(group,2)
my_comparisons=list()
for(i in 1:ncol(comp)){my_comparisons[[i]]<-comp[,i]}
my_comparisons=my_comparisons[2]


p1=ggplot(data=rt,aes(x =cancer, y=Expression, color=cancer))+
  stat_boxplot(geom="errorbar",width=0.5,size=1.3)+
  geom_boxplot(alpha=1,outlier.shape = NA,size=1.3,width=0.5,fatten=1)+
  theme_bw()+
  theme(panel.grid=element_blank())+
  stat_compare_means(comparisons =
my_comparisons,method="wilcox.test",symnum.args=list(cutpoints = c(0,
0.001, 0.01, 0.05, 1), symbols = c("***", "**", "*", "ns")),label =
"p.signif")+
  theme(legend.position = "right")+
  ggtitle("") +
  xlab("")+
  ylab("Rloopscore")+
  rotate_x_text(60)+
  theme(
    axis.title.x=element_blank(),
    axis.text.y = element_text(size = 15,face = "bold",color = "black"),
    axis.title.y = element_text(size = 15,face = "bold",color = "black"),
    axis.text.x = element_text(size = 15,face = "bold",color = "black"),
    legend.position = "none",

    plot.title = element_text(face = "bold",size=15,hjust = 0.5))
```

```
rt = rt1[,c(5,2)]
colnames(rt)=c("cancer","Expression")

group=levels(factor(rt$cancer))
rt$cancer=factor(rt$cancer, levels=group)
comp=combn(group,2)
my_comparisons=list()
for(i in 1:ncol(comp)){my_comparisons[[i]]<-comp[,i]}

ggplot(data=rt,aes(x =cancer, y=Expression, color=cancer))+
  stat_boxplot(geom="errorbar",width=0.5,size=1.3)+
  geom_boxplot(alpha=1,outlier.shape = NA,size=1.3,width=0.5,fatten=1)+
  theme_bw()+
  theme(panel.grid=element_blank())+
  stat_compare_means(comparisons =
my_comparisons,method="wilcox.test",symnum.args=list(cutpoints = c(0,
0.001, 0.01, 0.05, 1), symbols = c("***", "**", "*", "ns")),label =
"p.signif")+
  theme(legend.position = "right")+
  ggtitle("") +
  xlab("")+
  ylab("Rloopscore")+
  rotate_x_text(60)+
  theme(
    axis.title.x=element_blank(),
    axis.text.y = element_text(size = 15,face = "bold",color = "black"),
    axis.title.y = element_text(size = 15,face = "bold",color = "black"),
    axis.text.x = element_text(size = 15,face = "bold",color = "black"),
    legend.position = "none",

    plot.title = element_text(face = "bold",size=15,hjust = 0.5))




rt = rt1[,c(3,2)]
colnames(rt)=c("cancer","Expression")
rt[,"cancer"]=ifelse(rt[,"cancer"] <= 60, "<=60", ">60")

group=levels(factor(rt$cancer))
rt$cancer=factor(rt$cancer, levels=group)
comp=combn(group,2)
my_comparisons=list()
for(i in 1:ncol(comp)){my_comparisons[[i]]<-comp[,i]}

ggplot(data=rt,aes(x =cancer, y=Expression, color=cancer))+
  stat_boxplot(geom="errorbar",width=0.5,size=1.3)+
  geom_boxplot(alpha=1,outlier.shape = NA,size=1.3,width=0.5,fatten=1)+
  theme_bw()+
  theme(panel.grid=element_blank())+
  stat_compare_means(comparisons =
my_comparisons,method="wilcox.test",symnum.args=list(cutpoints = c(0,
0.001, 0.01, 0.05, 1), symbols = c("***", "**", "*", "ns")),label =
"p.signif")+
  theme(legend.position = "right")+
  ggtitle("") +
  xlab("")+
```

```
  ylab("Rloopscore")+
  rotate_x_text(60)+
  theme(
    axis.title.x=element_blank(),
    axis.text.y = element_text(size = 15,face = "bold",color = "black"),
    axis.title.y = element_text(size = 15,face = "bold",color = "black"),
    axis.text.x = element_text(size = 15,face = "bold",color = "black"),
    legend.position = "none",

    plot.title = element_text(face = "bold",size=15,hjust = 0.5))




rt = rt1[,c(4,2)]
colnames(rt)=c("cancer","Expression")

group=levels(factor(rt$cancer))
rt$cancer=factor(rt$cancer, levels=group)
comp=combn(group,2)
my_comparisons=list()
for(i in 1:ncol(comp)){my_comparisons[[i]]<-comp[,i]}

ggplot(data=rt,aes(x =cancer, y=Expression, color=cancer))+
  stat_boxplot(geom="errorbar",width=0.5,size=1.3)+
  geom_boxplot(alpha=1,outlier.shape = NA,size=1.3,width=0.5,fatten=1)+
  theme_bw()+
  theme(panel.grid=element_blank())+
  stat_compare_means(comparisons =
my_comparisons,method="wilcox.test",symnum.args=list(cutpoints = c(0,
0.001, 0.01, 0.05, 1), symbols = c("***", "**", "*", "ns")),label =
"p.signif")+
  theme(legend.position = "right")+
  ggtitle("") +
  xlab("")+
  ylab("Rloopscore")+
  rotate_x_text(60)+
  theme(
    axis.title.x=element_blank(),
    axis.text.y = element_text(size = 15,face = "bold",color = "black"),
    axis.title.y = element_text(size = 15,face = "bold",color = "black"),
    axis.text.x = element_text(size = 15,face = "bold",color = "black"),
    legend.position = "none",

    plot.title = element_text(face = "bold",size=15,hjust = 0.5))




#G----
load("scRNA.Rdata")
cli=read.table("cli.txt",sep="\t",header=T,check.names=F,row.names = 1)
cli = cli[17]
cli = cbind(id=rownames(cli),cli)

score=read.table("score.txt",sep="\t",header=T,check.names=F,row.names =
1)
```

```
score$type=ifelse(score$CRDscore <= median(score$CRDscore), "Low",
"High")

rt1 = merge(cli,score,by=0)

PD = rt1[rt1$Response == "PD",]
PD_Low = PD[PD$type == "Low",]
PD_High = PD[PD$type == "High",]

RD = rt1[rt1$Response == "RD",]
RD_Low = RD[RD$type == "Low",]
RD_High = RD[RD$type == "High",]


#PD
scRNA1 = scRNA[,PD[,1]]
counts = as.data.frame(scRNA1@assays[["RNA"]]@counts)

dge <- DGEList(
  counts = counts,
  norm.factors = rep(1,length(counts[1,])),
  group = PD$type
)

group_edgeR <- factor(PD$type)
design <- model.matrix(~group_edgeR)
rownames(design) <- colnames(dge)
o <- order(rowSums(dge$counts), decreasing=TRUE)
dge <- dge[o,]

dge <- calcNormFactors(dge)
dge <- estimateDisp(dge,design = design, robust=TRUE)
fit <- glmFit(dge,design)
res <- glmLRT(fit)
data = res[["table"]]
data = cbind(id=rownames(data),data)
data$FDR = p.adjust(data$PValue,method = "fdr")
write.table(data,file="diff_PD.txt",sep="\t",quote=F,row.names=F)


#RD
scRNA1 = scRNA[,RD[,1]]
counts = as.data.frame(scRNA1@assays[["RNA"]]@counts)

dge <- DGEList(
  counts = counts,
  norm.factors = rep(1,length(counts[1,])),
  group = RD$type
)

group_edgeR <- factor(RD$type)
design <- model.matrix(~group_edgeR)
rownames(design) <- colnames(dge)
o <- order(rowSums(dge$counts), decreasing=TRUE)
dge <- dge[o,]

dge <- calcNormFactors(dge)
dge <- estimateDisp(dge,design = design, robust=TRUE)
fit <- glmFit(dge,design)
res <- glmLRT(fit)
```

```
data = res[["table"]]
data = cbind(id=rownames(data),data)
data$FDR = p.adjust(data$PValue,method = "fdr")
write.table(data,file="diff_RD.txt",sep="\t",quote=F,row.names=F)


gene<-read.table("diff_PD.txt",sep="\t",header=T,check.names=F,row.names
= 1)

gene1<-str_trim(rownames(gene),"both")

gene2=bitr(gene1,fromType="SYMBOL",toType="ENTREZID",OrgDb="org.Hs.eg.db"
)

gene2 <- dplyr::distinct(gene2,SYMBOL,.keep_all=TRUE)
gene_df <- merge(gene2,gene,by.x="SYMBOL",by.y=0)

geneList<-gene_df $logFC
names(geneList)=gene_df $ENTREZID
geneList=sort(geneList,decreasing = T)


x <- readLines("KEGG_ENTREZID.gmt")
res <- strsplit(x, "\t")
names(res) <- vapply(res, function(y) y[1], character(1))
res <- lapply(res, "[", -c(1:2))
head(lapply(res, head))

fgseaRes <- fgsea(res, geneList, minSize=15, maxSize=500, nperm=1000)
fgseaRes1 <- fgsea(res, geneList, maxSize=500)
rt = fgseaRes1[,1:7]
write.table(rt,file="PD_fgseaRes.txt",sep="\t",quote=F,row.names=F)

pheatmap(rt,
         cluster_cols = F,
         cluster_rows = F,
         color = colorRampPalette(c("skyblue", "white", "red"))(50),
         show_colnames = F,
         border_color ="NA",
         fontsize = 14,
         fontsize_row=14,
         fontsize_col=6)

#RD
gene<-read.table("diff_RD.txt",sep="\t",header=T,check.names=F,row.names
= 1)
library(clusterProfiler)
library(org.Hs.eg.db)
library(stringr)
library(fgsea)
gene1<-str_trim(rownames(gene),"both")

gene2=bitr(gene1,fromType="SYMBOL",toType="ENTREZID",OrgDb="org.Hs.eg.db"
)

gene2 <- dplyr::distinct(gene2,SYMBOL,.keep_all=TRUE)
gene_df <- merge(gene2,gene,by.x="SYMBOL",by.y=0)

geneList<-gene_df $logFC
names(geneList)=gene_df $ENTREZID
```

```
geneList=sort(geneList,decreasing = T)

x <- readLines("KEGG_ENTREZID.gmt")
res <- strsplit(x, "\t")
names(res) <- vapply(res, function(y) y[1], character(1))
res <- lapply(res, "[", -c(1:2))
head(lapply(res, head))

fgseaRes <- fgsea(res, geneList, minSize=15, maxSize=500, nperm=1000)
fgseaRes1 <- fgsea(res, geneList, maxSize=500)
rt = fgseaRes1[,1:7]
write.table(rt,file="RD_fgseaRes.txt",sep="\t",quote=F,row.names=F)


pheatmap(rt,
         cluster_cols = F,
         cluster_rows = F,
         color = colorRampPalette(c("skyblue", "white", "red"))(50),
         show_colnames = F,
         border_color ="NA",
         fontsize = 14,
         fontsize_row=14,
         fontsize_col=6)



#H----
load("scRNA.Rdata")
meat = read.table("meat.txt", header=T, sep="\t", check.names=F,
row.names=1)
meat = meat[meat$singleR1 == "Maligant",]

scRNA = scRNA[,rownames(meat)]
dat = as.data.frame(scRNA@assays[["RNA"]]@counts)


library('GSEABase')
library(GSVA)
geneSets <- getGmt('metabolize.gmt')
GSVA_hall <- gsva(expr=as.matrix(dat),
                  gset.idx.list=geneSets,
                  mx.diff=T,
                  kcdf="Gaussian",
                  parallel.sz=14)



meat$Type = ifelse(meat$CRDscore > median(meat$CRDscore),"High","Low")
A2 = merge(t(GSVA_hall),meat[3],by=0)

low = A2[A2$Type == "Low",]
high =  A2[A2$Type == "High",]

A2 = A2[,-1]
rt=melt(A2,id.vars=c("Type"))
colnames(rt)=c("cancer","Gene","Expression")

group=levels(factor(rt$cancer))
rt$cancer=factor(rt$cancer, levels=group)
comp=combn(group,2)
```

```
my_comparisons=list()
for(i in 1:ncol(comp)){my_comparisons[[i]]<-comp[,i]}

ggplot(data=rt,aes(x = Gene, y=Expression, color=cancer))+
  scale_color_manual(values=c("#00AFBB", "#E7B800"))+
  geom_boxplot(aes(color=cancer),
               position=position_dodge(0.8),
               alpha=1,outlier.shape = NA,size=1.3,width=0.5,fatten=1,
               outlier.color = "white")+
  stat_compare_means(aes(group=cancer),
                     method="wilcox.test",
                     symnum.args=list(cutpoints = c(0, 0.001, 0.01, 0.05,
1), symbols = c("***", "**", "*", " ")),
                     label = "p.signif")+
  xlab('') +
  ylab('GSVA Score') +
  theme_bw()+
  theme(plot.title = element_text(hjust = 0.5),
        axis.text.x = element_text(angle = 90, hjust = 1))+
  theme(panel.grid.major =element_blank(),
        panel.grid.minor = element_blank(),
        panel.background = element_blank(),#
        panel.border = element_blank())


#I----
load("scRNA.Rdata")
cli <- read.table("cli.txt",header = T,sep = "\t",stringsAsFactors =
F,row.names = 1)
rloopscore <- fread(file="score.txt", header = T, sep='\t', data.table =
F)
rownames(rloopscore) = rloopscore[,1]

cli2 = merge(cli,rloopscore,by=0)
cli3 = cli2[cli2$singleR1 == "Maligant",]
rownames(cli3) = cli3[,1]

rloop = cli3[20]

exp = as.data.frame(scRNA@assays[["SCT"]]@counts)
exp = exp[,rownames(rloop)]


gene <- fread(file="gene1.txt", header = T, sep='\t', data.table = F)
A = gene[1]


for (i in A[,1]) {
  gene1=i
  gene2="Rloopscore"


  x=as.numeric(rloop[,1])
  y=as.numeric(exp[gene1,])


  df1=as.data.frame(cbind(x,y))
  corT=cor.test(x,y,method="pearson")
  cor=corT$estimate
  pValue=corT$p.value
```

```
  p1=ggplot(df1, aes(x, y)) +
    xlab(gene2)+ylab(gene1)+
    geom_point()+ geom_smooth(method="lm",formula = y ~ x) + theme_bw()+
    stat_cor(method = 'pearson', aes(x =x, y =y))


  data = cli3[15]
  data1 = t(exp[gene1,])
  rt = cbind(data,data1)
  colnames(rt)=c("cancer","Expression")
  rt$Expression = scale(rt$Expression)

  p=ggplot(data=rt,aes(y =Expression, x=cancer, color=cancer))+
    stat_boxplot(geom="errorbar",width=0.5,size=1.3)+
    geom_boxplot(alpha=1,outlier.shape = NA,size=1.3,width=0.5,fatten=1)+
    theme_bw()+
    theme(panel.grid=element_blank())+
    theme(legend.position = "right")+
    ggtitle("") +
    xlab("")+
    ylab("")+
    theme(
      axis.title.x=element_blank(),
      axis.text.y = element_text(size = 15,face = "bold",color =
"black"),
      axis.title.y = element_text(size = 15,face = "bold",color =
"black"),
      axis.text.x = element_text(size = 15,face = "bold",color =
"black"),
      legend.position = "none",

      plot.title = element_text(face = "bold",size=15,hjust = 0.5))




  rt = cbind(data,rloop)
  colnames(rt)=c("cancer","Expression")
  rt$Expression = scale(rt$Expression)



  p2=ggplot(data=rt,aes(y =cancer, x=Expression, color=cancer))+
    stat_boxplot(geom="errorbar",width=0.5,size=1.3)+
    geom_boxplot(alpha=1,outlier.shape = NA,size=1.3,width=0.5,fatten=1)+
    theme_bw()+
    theme(panel.grid=element_blank())+
    theme(legend.position = "right")+
    ggtitle("") +
    xlab("")+
    ylab("")+
    theme(
      axis.title.x=element_blank(),
      axis.text.y = element_text(size = 15,face = "bold",color =
"black"),
      axis.title.y = element_text(size = 15,face = "bold",color =
"black"),
      axis.text.x = element_text(size = 15,face = "bold",color =
"black"),
```

```
      legend.position = "none",

      plot.title = element_text(face = "bold",size=15,hjust = 0.5))

  pp = (p1+p)+plot_layout(widths = c(5,1))
  pp1 = p2+plot_spacer()+plot_layout(widths = c(5,1))
  pp2 = pp1/pp+plot_layout(heights = c(1, 5))
  pdf(file=paste0("./Energy/",i,"_box.pdf"),width=5,height=5)
  print(pp2)
  dev.off()


}


gene <- fread(file="gene.txt", header = F, sep='\t', data.table = F)
rownames(gene) = gene[,1]
gene = t(gene)


load("scRNA.Rdata")

cli <- read.table("cli.txt",header = T,sep = "\t",stringsAsFactors =
F,row.names = 1)
cli = cli[cli$singleR1 == "Maligant",]
rloop = cli[17]

exp = as.data.frame(scRNA@assays[["SCT"]]@counts)
exp = exp[,rownames(rloop)]

data = exp[,rownames(cli)]
rloop = cli[17]

gene_name1<-c()
gene_name2<-c()
cor_r<-c()
pvalue<-c()

for (i in 1:ncol(gene)) {
  A = gene[i]
  A = na.omit(A)
  for (j in A[,1]) {
    g1= colnames(gene)[i]
    g2=j

    if (length(na.omit(t(data[j,]))) == 0 ){
      next
    }
    c_r=cor(as.numeric(rloop[,1]),as.numeric(data[j,]),method="pearson")
    gene_name1=c(gene_name1,g1)
    gene_name2=c(gene_name2,g2)
    cor_r=c(cor_r,c_r)

  }

}

data_cor<-data.frame(gene_name1,gene_name2,cor_r)

rt = data_cor[1:20,]
```

```
ggplot(rt, aes(x = reorder(gene_name1, cor_r, FUN = median),y=cor_r, fill
= "#a559b3")) +
  geom_col() +coord_flip()+labs(y = "Correlation",x = "")+
  theme(panel.grid.major =element_blank(),
        panel.grid.minor = element_blank(),
        panel.background = element_blank(),
        panel.border = element_blank())



#fig5----
#A----
#LUAD

library(rms)
data = read.table("score.txt", header=T, sep="\t",
check.names=F,row.names = 1)

dd <- datadist(data)
options(datadist='dd')

fit<- cph(Surv(futime,fustat) ~ rcs(CRDscore,3) ,data=data)

dd$limits$CRDscore[2] <- median(data$CRDscore)
fit=update(fit)
HR<-Predict(fit, CRDscore,fun=exp,ref.zero = TRUE)
P1<-ggplot(HR)
P1

P2<-
ggplot()+geom_line(data=HR,aes(CRDscore,yhat),linetype="solid",size=1,alp
ha = 0.7,colour="red")+
  geom_ribbon(data=HR, aes(CRDscore,ymin = lower, ymax = upper),alpha =
0.1,fill="red")
P3<-P2 +theme(panel.grid.major =element_blank(),panel.grid.minor =
element_blank(),

              panel.background = element_blank(),axis.line =
element_line(colour = "black"))+


  geom_hline(yintercept=1,
             linetype=2,size=1)+
  labs( x="CRDscore", y="Ln HR(Z,Zref)",ref.label="Ref.")+
  geom_vline(xintercept = median(data$CRDscore),linetype=1,size=1)
P3



library("survival")
library("survminer")

inputFile="CRDscore-cutoff.txt"
var="CRDscore"
rt1=read.table(inputFile,header=T,sep="\t",check.names=F)
rt1=rt1[,c("futime","fustat",var)]
group=ifelse(rt1[,3]>median(rt1[,3]),"High","Low")
diff=survdiff(Surv(futime, fustat) ~group,data = rt1)
pValue=1-pchisq(diff$chisq,df=1)
```

```
if(pValue<0.001){
  pValue="p<0.001"
}else{
  pValue=paste0("p=",sprintf("%.03f",pValue))
}
fit <- survfit(Surv(futime, fustat) ~ group, data = rt1)

ggsurvplot(
  fit, #
  pval = TRUE, #
  pval.method = TRUE,
  pval.size=5,#
  legend.title='Rloopscore', #
  legend.labs=c('High','Low'), #
  conf.int = TRUE, #

  linetype = "strata",
  xlab="Time(years)",
  break.time.by = 2,#
  surv.median.line = "hv",
  ggtheme = theme_bw(), #
  palette = c("#E31A1C","#1F78B4") #
)

#GSE13213

library(CRDscore)
GSE=read.table("GSE13213_series_matrix.txt",sep="\t",header=T,check.names
=F,row.names = 1)
ann=read.table("ann.txt",sep="\t",header=T,check.names=F,row.names = 1)
data = merge(ann,GSE,by=0)
data = na.omit(data)

rt=as.matrix(data)
rt = rt[,-1]
rownames(rt)=rt[,1]
exp=rt[,2:ncol(rt)]
dimnames=list(rownames(exp),colnames(exp))
mat=matrix(as.numeric(as.matrix(exp)),nrow=nrow(exp),dimnames=dimnames)
mat=avereps(mat)


moduleLabels=read.table("moduleLabels.txt",sep="\t",header=T,check.names=
F,row.names = 1)
moduleLabels = moduleLabels[moduleLabels$moduleLabels == "9" |
moduleLabels$moduleLabels == "5" |
                              moduleLabels$moduleLabels == "10" |
moduleLabels$moduleLabels == "8",]

os=read.table("os.txt",sep="\t",header=T,check.names=F,row.names = 1)


CRDscore <- cal_CRDscore(expr = mat, n.bins = 50, circadians =
rownames(moduleLabels), study.type = "bulk_RNAseq")
D = as.data.frame(CRDscore)
D = merge(D,os,by.x=0,by.y=0)
D$futime = D$futime/365


data = D
```

```
write.table(data,file="CRDscore.txt",sep="\t",quote=F,row.names=F)
library(survival)
library(survminer)
var="CRDscore"

rt1=data[,c("futime","fustat",var)]


group=ifelse(rt1[,3]>median(rt1[,3]),"High","Low")

diff=survdiff(Surv(futime, fustat) ~group,data = rt1)
pValue=1-pchisq(diff$chisq,df=1)
if(pValue<0.001){
  pValue="p<0.001"
}else{
  pValue=paste0("p=",sprintf("%.03f",pValue))
}
fit <- survfit(Surv(futime, fustat) ~ group, data = rt1)

ggsurvplot(
  fit, #
  pval = TRUE,
  pval.method = TRUE,
  pval.size=5,#
  legend.title='Rloopscore',
  legend.labs=c('High','Low'),
  linetype = "strata",
  xlab="Time(years)",
  break.time.by = 4,#
  surv.median.line = "hv",
  ggtheme = theme_bw(),
  palette = c("#E31A1C","#1F78B4")
)



library(rms)
data = read.table("CRDscore.txt", header=T, sep="\t",
check.names=F,row.names = 1)

dd <- datadist(data)
options(datadist='dd')

fit<- cph(Surv(futime,fustat) ~ rcs(CRDscore,3) ,data=data)

dd$limits$CRDscore[2] <- median(data$CRDscore)
fit=update(fit)
HR<-Predict(fit, CRDscore,fun=exp,ref.zero = TRUE)
P1<-ggplot(HR)
P1

P2<-
ggplot()+geom_line(data=HR,aes(CRDscore,yhat),linetype="solid",size=1,alp
ha = 0.7,colour="red")+
  geom_ribbon(data=HR, aes(CRDscore,ymin = lower, ymax = upper),alpha =
0.1,fill="red")
P3<-P2 +theme_bw()+
  geom_hline(yintercept=1,
             linetype=2,size=1)+
  labs( x="CRDscore", y="Ln HR(Z,Zref)",ref.label="Ref.")+
```

```
  geom_vline(xintercept = median(data$CRDscore),linetype=1,size=1)
P3





#GSE30219

GSE=read.table("GSE30219_series_matrix.txt",sep="\t",header=T,check.names
=F,row.names = 1)
ann=read.table("ann.txt",sep="\t",header=T,check.names=F,row.names = 1)
data = merge(ann,GSE,by=0)
data = na.omit(data)

rt=as.matrix(data)
rt = rt[,-1]
rownames(rt)=rt[,1]
exp=rt[,2:ncol(rt)]
dimnames=list(rownames(exp),colnames(exp))
mat=matrix(as.numeric(as.matrix(exp)),nrow=nrow(exp),dimnames=dimnames)
mat=avereps(mat)


moduleLabels=read.table("moduleLabels.txt",sep="\t",header=T,check.names=
F,row.names = 1)
moduleLabels = moduleLabels[moduleLabels$moduleLabels == "9" |
moduleLabels$moduleLabels == "5" |
                              moduleLabels$moduleLabels == "10" |
moduleLabels$moduleLabels == "8",]

os=read.table("os.txt",sep="\t",header=T,check.names=F,row.names = 1)


CRDscore <- cal_CRDscore(expr = mat, n.bins = 50, circadians =
rownames(moduleLabels), study.type = "bulk_RNAseq")
D = as.data.frame(CRDscore)
D = merge(D,os,by.x=0,by.y=0)
D$futime = D$futime/30


data = D
write.table(data,file="CRDscore.txt",sep="\t",quote=F,row.names=F)
library(survival)
library(survminer)
var="CRDscore"

rt1=data[,c("futime","fustat",var)]


group=ifelse(rt1[,3]>median(rt1[,3]),"High","Low")
diff=survdiff(Surv(futime, fustat) ~group,data = rt1)
pValue=1-pchisq(diff$chisq,df=1)
if(pValue<0.001){
  pValue="p<0.001"
}else{
  pValue=paste0("p=",sprintf("%.03f",pValue))
}
fit <- survfit(Surv(futime, fustat) ~ group, data = rt1)
```

```
P = ggsurvplot(
  fit, #
  pval = TRUE, #
  pval.method = TRUE,
  pval.size=5,#
  legend.title='Rloopscore', #
  legend.labs=c('High','Low'), #
  linetype = "strata",
  xlab="Time(Months)",
  break.time.by = 4,#
  surv.median.line = "hv",
  ggtheme = theme_bw(),
  palette = c("#E31A1C","#1F78B4")
)



library(rms)
data = read.table("CRDscore.txt", header=T, sep="\t",
check.names=F,row.names = 1)

dd <- datadist(data)
options(datadist='dd')

fit<- cph(Surv(futime,fustat) ~ rcs(CRDscore,3) ,data=data)

dd$limits$CRDscore[2] <- median(data$CRDscore)
fit=update(fit)
HR<-Predict(fit, CRDscore,fun=exp,ref.zero = TRUE)
P1<-ggplot(HR)
P1

P2<-
ggplot()+geom_line(data=HR,aes(CRDscore,yhat),linetype="solid",size=1,alp
ha = 0.7,colour="red")+
  geom_ribbon(data=HR, aes(CRDscore,ymin = lower, ymax = upper),alpha =
0.1,fill="red")
P3<-P2 +theme_bw()+

  geom_hline(yintercept=1,
             linetype=2,size=1)+
  labs( x="CRDscore", y="Ln HR(Z,Zref)",ref.label="Ref.")+
  geom_vline(xintercept = median(data$CRDscore),linetype=1,size=1)
P3




#GSE31210
GSE=read.table("exp.txt",sep="\t",header=T,check.names=F,row.names = 1)
exp=GSE
dimnames=list(rownames(exp),colnames(exp))
mat=matrix(as.numeric(as.matrix(exp)),nrow=nrow(exp),dimnames=dimnames)
mat=avereps(mat)
mat = log2(mat+1)


moduleLabels=read.table("moduleLabels.txt",sep="\t",header=T,check.names=
F,row.names = 1)
```

```
moduleLabels = moduleLabels[moduleLabels$moduleLabels == "9" |
moduleLabels$moduleLabels == "5" |
                              moduleLabels$moduleLabels == "10" |
moduleLabels$moduleLabels == "8",]

os=read.table("os.txt",sep="\t",header=T,check.names=F,row.names = 1)


CRDscore <- cal_CRDscore(expr = mat, n.bins = 50, circadians =
rownames(moduleLabels), study.type = "bulk_RNAseq")
D = as.data.frame(CRDscore)
D = merge(D,os,by.x=0,by.y=0)
D$futime = D$futime/365


data = D
write.table(data,file="CRDscore.txt",sep="\t",quote=F,row.names=F)
library(survival)
library(survminer)
var="CRDscore"

rt1=data[,c("futime","fustat",var)]


group=ifelse(rt1[,3]>median(rt1[,3]),"High","Low")

diff=survdiff(Surv(futime, fustat) ~group,data = rt1)
pValue=1-pchisq(diff$chisq,df=1)
if(pValue<0.001){
  pValue="p<0.001"
}else{
  pValue=paste0("p=",sprintf("%.03f",pValue))
}
fit <- survfit(Surv(futime, fustat) ~ group, data = rt1)

P = ggsurvplot(
  fit, #
  pval = TRUE, #
  pval.method = TRUE,
  pval.size=5,#
  legend.title='Rloopscore',
  legend.labs=c('High','Low'),
  linetype = "strata",
  xlab="Time(years)",
  break.time.by = 4,
  surv.median.line = "hv",
  ggtheme = theme_bw(),
  palette = c("#E31A1C","#1F78B4")
)



library(rms)
data = read.table("CRDscore.txt", header=T, sep="\t",
check.names=F,row.names = 1)

dd <- datadist(data)
options(datadist='dd')

fit<- cph(Surv(futime,fustat) ~ rcs(CRDscore,3) ,data=data)
```

```
dd$limits$CRDscore[2] <- median(data$CRDscore)
fit=update(fit)
HR<-Predict(fit, CRDscore,fun=exp,ref.zero = TRUE)
P1<-ggplot(HR)
P1

P2<-
ggplot()+geom_line(data=HR,aes(CRDscore,yhat),linetype="solid",size=1,alp
ha = 0.7,colour="red")+
  geom_ribbon(data=HR, aes(CRDscore,ymin = lower, ymax = upper),alpha =
0.1,fill="red")
P3<-P2 +theme_bw()+

  geom_hline(yintercept=1,
             linetype=2,size=1)+
  labs( x="CRDscore", y="Ln HR(Z,Zref)",ref.label="Ref.")+
  geom_vline(xintercept = median(data$CRDscore),linetype=1,size=1)
P3


#GSE41271

GSE=read.table("GSE41271_series_matrix.txt",sep="\t",header=T,check.names
=F,row.names = 1)
ann=read.table("ann.txt",sep="\t",header=T,check.names=F,row.names = 1)
data = merge(ann,GSE,by=0)
data = na.omit(data)

rt=as.matrix(data)
rt = rt[,-1]
rownames(rt)=rt[,1]
exp=rt[,2:ncol(rt)]
dimnames=list(rownames(exp),colnames(exp))
mat=matrix(as.numeric(as.matrix(exp)),nrow=nrow(exp),dimnames=dimnames)
mat=avereps(mat)


moduleLabels=read.table("moduleLabels.txt",sep="\t",header=T,check.names=
F,row.names = 1)
moduleLabels = moduleLabels[moduleLabels$moduleLabels == "9" |
moduleLabels$moduleLabels == "5" |
                              moduleLabels$moduleLabels == "10" |
moduleLabels$moduleLabels == "8",]

os=read.table("os.txt",sep="\t",header=T,check.names=F,row.names = 1)


CRDscore <- cal_CRDscore(expr = mat, n.bins = 50, circadians =
rownames(moduleLabels), study.type = "bulk_RNAseq")
D = as.data.frame(CRDscore)
D = merge(D,os,by.x=0,by.y=0)

begin = as.Date(D$start)
end = as.Date(D$end)
C = as.data.frame(difftime(end, begin, units = "days"))
C$`difftime(end, begin, units = "days")` = gsub(" days",replacement =
"",C$`difftime(end, begin, units = "days")`)
D$futime = C$`difftime(end, begin, units = "days")`
D$futime = as.numeric(D$futime)
```

```
D$futime = D$futime/365


data = D
write.table(data,file="CRDscore.txt",sep="\t",quote=F,row.names=F)
library(survival)
library(survminer)
var="CRDscore"

rt1=data[,c("futime","fustat",var)]
rt1$fustat = as.numeric(rt1$fustat)


group=ifelse(rt1[,3]>median(rt1[,3]),"High","Low")
diff=survdiff(Surv(futime, fustat) ~group,data = rt1)
pValue=1-pchisq(diff$chisq,df=1)
if(pValue<0.001){
  pValue="p<0.001"
}else{
  pValue=paste0("p=",sprintf("%.03f",pValue))
}
fit <- survfit(Surv(futime, fustat) ~ group, data = rt1)

ggsurvplot(
  fit,
  pval = TRUE,
  pval.method = TRUE,
  pval.size=5,
  legend.title='Rloopscore',
  legend.labs=c('High','Low'),
  linetype = "strata",
  xlab="Time(years)",
  break.time.by = 4,
  surv.median.line = "hv",
  ggtheme = theme_bw(),
  palette = c("#E31A1C","#1F78B4")
)





library(rms)
data = read.table("CRDscore.txt", header=T, sep="\t",
check.names=F,row.names = 1)
data$fustat = as.numeric(data$fustat)

dd <- datadist(data)
options(datadist='dd')
fit<- cph(Surv(futime,fustat) ~ rcs(CRDscore,3) ,data=data)

dd$limits$CRDscore[2] <- median(data$CRDscore)
fit=update(fit)
HR<-Predict(fit, CRDscore,fun=exp,ref.zero = TRUE)
P1<-ggplot(HR)
P1

P2<-
ggplot()+geom_line(data=HR,aes(CRDscore,yhat),linetype="solid",size=1,alp
ha = 0.7,colour="red")+
```

```
  geom_ribbon(data=HR, aes(CRDscore,ymin = lower, ymax = upper),alpha =
0.1,fill="red")
P3<-P2 +theme_bw()+

  geom_hline(yintercept=1,
             linetype=2,size=1)+
  labs( x="CRDscore", y="Ln HR(Z,Zref)",ref.label="Ref.")+
  geom_vline(xintercept = median(data$CRDscore),linetype=1,size=1)
P3




#GSE50081

GSE=read.table("GSE50081_series_matrix.txt",sep="\t",header=T,check.names
=F,row.names = 1)
ann=read.table("ann.txt",sep="\t",header=T,check.names=F,row.names = 1)
data = merge(ann,GSE,by=0)
data = na.omit(data)

rt=as.matrix(data)
rt = rt[,-1]
rownames(rt)=rt[,1]
exp=rt[,2:ncol(rt)]
dimnames=list(rownames(exp),colnames(exp))
mat=matrix(as.numeric(as.matrix(exp)),nrow=nrow(exp),dimnames=dimnames)
mat=avereps(mat)


moduleLabels=read.table("moduleLabels.txt",sep="\t",header=T,check.names=
F,row.names = 1)
moduleLabels = moduleLabels[moduleLabels$moduleLabels == "9" |
moduleLabels$moduleLabels == "5" |
                               moduleLabels$moduleLabels == "10" |
moduleLabels$moduleLabels == "8",]

os=read.table("os.txt",sep="\t",header=T,check.names=F,row.names = 1)


CRDscore <- cal_CRDscore(expr = mat, n.bins = 50, circadians =
rownames(moduleLabels), study.type = "bulk_RNAseq")
D = as.data.frame(CRDscore)
D = merge(D,os,by.x=0,by.y=0)


data = D
write.table(data,file="CRDscore.txt",sep="\t",quote=F,row.names=F)
library(survival)
library(survminer)
var="CRDscore"

rt1=data[,c("futime","fustat",var)]

res.cut=surv_cutpoint(rt1, time = "futime", event = "fustat", variables
=c("CRDscore"))
cutoff=as.numeric(res.cut$cutpoint[1])
group=ifelse(rt1[,3]<=cutoff, "Low", "High")
```

```
diff=survdiff(Surv(futime, fustat) ~group,data = rt1)
pValue=1-pchisq(diff$chisq,df=1)
if(pValue<0.001){
  pValue="p<0.001"
}else{
  pValue=paste0("p=",sprintf("%.03f",pValue))
}
fit <- survfit(Surv(futime, fustat) ~ group, data = rt1)

ggsurvplot(
  fit,
  pval = TRUE,
  pval.method = TRUE,
  pval.size=5,
  legend.title='Rloopscore',
  legend.labs=c('High','Low'),
  linetype = "strata",
  xlab="Time(years)",
  break.time.by = 4,
  surv.median.line = "hv",
  ggtheme = theme_bw(),
  palette = c("#E31A1C","#1F78B4")
)




library(rms)
data = read.table("CRDscore.txt", header=T, sep="\t",
check.names=F,row.names = 1)

dd <- datadist(data)
options(datadist='dd')
fit<- cph(Surv(futime,fustat) ~ rcs(CRDscore,3) ,data=data)

dd$limits$CRDscore[2] <- median(data$CRDscore)
fit=update(fit)
HR<-Predict(fit, CRDscore,fun=exp,ref.zero = TRUE)
P1<-ggplot(HR)
P1

P2<-
ggplot()+geom_line(data=HR,aes(CRDscore,yhat),linetype="solid",size=1,alp
ha = 0.7,colour="red")+
  geom_ribbon(data=HR, aes(CRDscore,ymin = lower, ymax = upper),alpha =
0.1,fill="red")
P3<-P2 +theme_bw()+

  geom_hline(yintercept=1,
             linetype=2,size=1)+
  labs( x="CRDscore", y="Ln HR(Z,Zref)",ref.label="Ref.")+
  geom_vline(xintercept = median(data$CRDscore),linetype=1,size=1)
P3



#GSE72094
GSE=read.table("GSE72094_series_matrix.txt",sep="\t",header=T,check.names
=F,row.names = 1)
```

```
ann=read.table("ann_1.txt",sep="\t",header=T,check.names=F,row.names = 1)
data = merge(ann,GSE,by=0)
data = na.omit(data)

rt=as.matrix(data)
rt = rt[,-1]
rownames(rt)=rt[,1]
exp=rt[,2:ncol(rt)]
dimnames=list(rownames(exp),colnames(exp))
mat=matrix(as.numeric(as.matrix(exp)),nrow=nrow(exp),dimnames=dimnames)
mat=avereps(mat)


moduleLabels=read.table("moduleLabels.txt",sep="\t",header=T,check.names=
F,row.names = 1)
moduleLabels = moduleLabels[moduleLabels$moduleLabels == "9" |
moduleLabels$moduleLabels == "5" |
                              moduleLabels$moduleLabels == "10" |
moduleLabels$moduleLabels == "8",]

os=read.table("os.txt",sep="\t",header=T,check.names=F,row.names = 1)


CRDscore <- cal_CRDscore(expr = mat, n.bins = 50, circadians =
rownames(moduleLabels), study.type = "bulk_RNAseq")
D = as.data.frame(CRDscore)
D = merge(D,os,by.x=0,by.y=0)
D$futime = D$futime/365


data = D
write.table(data,file="CRDscore.txt",sep="\t",quote=F,row.names=F)
library(survival)
library(survminer)
var="CRDscore"

rt1=data[,c("futime","fustat",var)]


group=ifelse(rt1[,3]>median(rt1[,3]),"High","Low")
diff=survdiff(Surv(futime, fustat) ~group,data = rt1)
pValue=1-pchisq(diff$chisq,df=1)
if(pValue<0.001){
  pValue="p<0.001"
}else{
  pValue=paste0("p=",sprintf("%.03f",pValue))
}
fit <- survfit(Surv(futime, fustat) ~ group, data = rt1)

ggsurvplot(
  fit,
  pval = TRUE,
  pval.method = TRUE,
  pval.size=5,
  legend.title='Rloopscore',
  legend.labs=c('High','Low'),
  linetype = "strata",
  xlab="Time(years)",
  break.time.by = 4,
  surv.median.line = "hv",
```

```
  ggtheme = theme_bw(),
  palette = c("#E31A1C","#1F78B4")
)


library(rms)
data = read.table("CRDscore.txt", header=T, sep="\t",
check.names=F,row.names = 1)

dd <- datadist(data)
options(datadist='dd')
fit<- cph(Surv(futime,fustat) ~ rcs(CRDscore,3) ,data=data)

dd$limits$CRDscore[2] <- median(data$CRDscore)
fit=update(fit)
HR<-Predict(fit, CRDscore,fun=exp,ref.zero = TRUE)
P1<-ggplot(HR)
P1

P2<-
ggplot()+geom_line(data=HR,aes(CRDscore,yhat),linetype="solid",size=1,alp
ha = 0.7,colour="red")+
  geom_ribbon(data=HR, aes(CRDscore,ymin = lower, ymax = upper),alpha =
0.1,fill="red")
P3<-P2 +theme_bw()+
  geom_hline(yintercept=1,
             linetype=2,size=1)+
  labs( x="CRDscore", y="Ln HR(Z,Zref)",ref.label="Ref.")+
  geom_vline(xintercept = median(data$CRDscore),linetype=1,size=1)
P3




#B----

os=read.table("Total.txt",sep="\t",header=T,check.names=F,row.names = 1)
inputFile="AGe.txt"

data=read.table(inputFile,header=T,sep="\t",check.names=F,row.names = 1)
data = merge(os,data,by=0)
table(data$age)

rt = data[data$age == "<=60",]
rt = rt[,-c(1,5)]
outTab = c()

cox <- coxph(Surv(futime, fustat) ~ rt[,1], data = rt)
coxSummary = summary(cox)
coxP=coxSummary$coefficients[,"Pr(>|z|)"]
outTab=rbind(outTab,
             cbind(id="<=60",
                   HR=coxSummary$conf.int[,"exp(coef)"],
                   HR.95L=coxSummary$conf.int[,"lower .95"],
                   HR.95H=coxSummary$conf.int[,"upper .95"],
                   pvalue=coxSummary$coefficients[,"Pr(>|z|)"])
)
A = outTab
rt = data[data$age == ">60",]
rt = rt[,-c(1,5)]
```

```
outTab = c()

cox <- coxph(Surv(futime, fustat) ~ rt[,1], data = rt)
coxSummary = summary(cox)
coxP=coxSummary$coefficients[,"Pr(>|z|)"]
outTab=rbind(outTab,
             cbind(id=">60",
                   HR=coxSummary$conf.int[,"exp(coef)"],
                   HR.95L=coxSummary$conf.int[,"lower .95"],
                   HR.95H=coxSummary$conf.int[,"upper .95"],
                   pvalue=coxSummary$coefficients[,"Pr(>|z|)"])
)
rt = rbind(A,outTab)
write.table(rt, file="HR_Age.txt", sep="\t", quote=F, row.names=F)


os=read.table("Total.txt",sep="\t",header=T,check.names=F,row.names = 1)
inputFile="Gender.txt"

data=read.table(inputFile,header=T,sep="\t",check.names=F,row.names = 1)
data = merge(os,data,by=0)
table(data$gender)

rt = data[data$gender == "F",]
rt = rt[,-c(1,5)]
outTab = c()

cox <- coxph(Surv(futime, fustat) ~ rt[,1], data = rt)
coxSummary = summary(cox)
coxP=coxSummary$coefficients[,"Pr(>|z|)"]
outTab=rbind(outTab,
             cbind(id="F",
                   HR=coxSummary$conf.int[,"exp(coef)"],
                   HR.95L=coxSummary$conf.int[,"lower .95"],
                   HR.95H=coxSummary$conf.int[,"upper .95"],
                   pvalue=coxSummary$coefficients[,"Pr(>|z|)"])
)
A = outTab



rt = data[data$gender == "M",]
rt = rt[,-c(1,5)]
outTab = c()

cox <- coxph(Surv(futime, fustat) ~ rt[,1], data = rt)
coxSummary = summary(cox)
coxP=coxSummary$coefficients[,"Pr(>|z|)"]
outTab=rbind(outTab,
             cbind(id="M",
                   HR=coxSummary$conf.int[,"exp(coef)"],
                   HR.95L=coxSummary$conf.int[,"lower .95"],
                   HR.95H=coxSummary$conf.int[,"upper .95"],
                   pvalue=coxSummary$coefficients[,"Pr(>|z|)"])
)
rt = rbind(A,outTab)
write.table(rt, file="HR_Gender.txt", sep="\t", quote=F, row.names=F)


os=read.table("Total.txt",sep="\t",header=T,check.names=F,row.names = 1)
```

```
inputFile="Smoker.txt"

data=read.table(inputFile,header=T,sep="\t",check.names=F,row.names = 1)
data = merge(os,data,by=0)
table(data$smoker)

rt = data[data$smoker == "Smoker",]
rt = rt[,-c(1,5)]
outTab = c()

cox <- coxph(Surv(futime, fustat) ~ rt[,1], data = rt)
coxSummary = summary(cox)
coxP=coxSummary$coefficients[,"Pr(>|z|)"]
outTab=rbind(outTab,
             cbind(id="Smoker",
                   HR=coxSummary$conf.int[,"exp(coef)"],
                   HR.95L=coxSummary$conf.int[,"lower .95"],
                   HR.95H=coxSummary$conf.int[,"upper .95"],
                   pvalue=coxSummary$coefficients[,"Pr(>|z|)"])
)
A = outTab

rt = data[data$smoker == "Never Smoker",]
rt = rt[,-c(1,5)]
outTab = c()

cox <- coxph(Surv(futime, fustat) ~ rt[,1], data = rt)
coxSummary = summary(cox)
coxP=coxSummary$coefficients[,"Pr(>|z|)"]
outTab=rbind(outTab,
             cbind(id="Never Smoker",
                   HR=coxSummary$conf.int[,"exp(coef)"],
                   HR.95L=coxSummary$conf.int[,"lower .95"],
                   HR.95H=coxSummary$conf.int[,"upper .95"],
                   pvalue=coxSummary$coefficients[,"Pr(>|z|)"])
)
rt = rbind(A,outTab)
write.table(rt, file="HR_Smoker.txt", sep="\t", quote=F, row.names=F)


#Stage
os=read.table("Total.txt",sep="\t",header=T,check.names=F,row.names = 1)
inputFile="Stage.txt"

data=read.table(inputFile,header=T,sep="\t",check.names=F,row.names = 1)
data = merge(os,data,by=0)

rt = data[data$Stage == "I-II",]
rt = rt[,-c(1,5)]
outTab = c()

cox <- coxph(Surv(futime, fustat) ~ rt[,1], data = rt)
coxSummary = summary(cox)
coxP=coxSummary$coefficients[,"Pr(>|z|)"]
outTab=rbind(outTab,
             cbind(id="I-II",
                   HR=coxSummary$conf.int[,"exp(coef)"],
                   HR.95L=coxSummary$conf.int[,"lower .95"],
                   HR.95H=coxSummary$conf.int[,"upper .95"],
                   pvalue=coxSummary$coefficients[,"Pr(>|z|)"])
```

```
)
A = outTab



rt = data[data$Stage == "III-IV",]
rt = rt[,-c(1,5)]
outTab = c()

cox <- coxph(Surv(futime, fustat) ~ rt[,1], data = rt)
coxSummary = summary(cox)
coxP=coxSummary$coefficients[,"Pr(>|z|)"]
outTab=rbind(outTab,
             cbind(id="III-IV",
                   HR=coxSummary$conf.int[,"exp(coef)"],
                   HR.95L=coxSummary$conf.int[,"lower .95"],
                   HR.95H=coxSummary$conf.int[,"upper .95"],
                   pvalue=coxSummary$coefficients[,"Pr(>|z|)"])
)
rt = rbind(A,outTab)
write.table(rt, file="HR_Stage.txt", sep="\t", quote=F, row.names=F)



#EGFR
os=read.table("Total.txt",sep="\t",header=T,check.names=F,row.names = 1)
inputFile="EGFR.txt"

data=read.table(inputFile,header=T,sep="\t",check.names=F,row.names = 1)
data = merge(os,data,by=0)

rt = data[data$egfr == "WT",]
rt = rt[,-c(1,5)]
outTab = c()

cox <- coxph(Surv(futime, fustat) ~ rt[,1], data = rt)
coxSummary = summary(cox)
coxP=coxSummary$coefficients[,"Pr(>|z|)"]
outTab=rbind(outTab,
             cbind(id="WT",
                   HR=coxSummary$conf.int[,"exp(coef)"],
                   HR.95L=coxSummary$conf.int[,"lower .95"],
                   HR.95H=coxSummary$conf.int[,"upper .95"],
                   pvalue=coxSummary$coefficients[,"Pr(>|z|)"])
)
A = outTab



rt = data[data$egfr == "Mut",]
rt = rt[,-c(1,5)]
outTab = c()

cox <- coxph(Surv(futime, fustat) ~ rt[,1], data = rt)
coxSummary = summary(cox)
coxP=coxSummary$coefficients[,"Pr(>|z|)"]
outTab=rbind(outTab,
             cbind(id="Mut",
                   HR=coxSummary$conf.int[,"exp(coef)"],
                   HR.95L=coxSummary$conf.int[,"lower .95"],
```

```
                    HR.95H=coxSummary$conf.int[,"upper .95"],
                    pvalue=coxSummary$coefficients[,"Pr(>|z|)"])
)
rt = rbind(A,outTab)
write.table(rt, file="HR_EGFR.txt", sep="\t", quote=F, row.names=F)



#KRAS

os=read.table("Total.txt",sep="\t",header=T,check.names=F,row.names = 1)
inputFile="KRAS.txt"

data=read.table(inputFile,header=T,sep="\t",check.names=F,row.names = 1)
data = merge(os,data,by=0)

rt = data[data$kras == "WT",]
rt = rt[,-c(1,5)]
outTab = c()

cox <- coxph(Surv(futime, fustat) ~ rt[,1], data = rt)
coxSummary = summary(cox)
coxP=coxSummary$coefficients[,"Pr(>|z|)"]
outTab=rbind(outTab,
             cbind(id="WT",
                   HR=coxSummary$conf.int[,"exp(coef)"],
                   HR.95L=coxSummary$conf.int[,"lower .95"],
                   HR.95H=coxSummary$conf.int[,"upper .95"],
                   pvalue=coxSummary$coefficients[,"Pr(>|z|)"])
)
A = outTab



rt = data[data$kras == "Mut",]
rt = rt[,-c(1,5)]
outTab = c()

cox <- coxph(Surv(futime, fustat) ~ rt[,1], data = rt)
coxSummary = summary(cox)
coxP=coxSummary$coefficients[,"Pr(>|z|)"]
outTab=rbind(outTab,
             cbind(id="Mut",
                   HR=coxSummary$conf.int[,"exp(coef)"],
                   HR.95L=coxSummary$conf.int[,"lower .95"],
                   HR.95H=coxSummary$conf.int[,"upper .95"],
                   pvalue=coxSummary$coefficients[,"Pr(>|z|)"])
)
rt = rbind(A,outTab)
write.table(rt, file="HR_KRAS.txt", sep="\t", quote=F, row.names=F)



#TP53

os=read.table("Total.txt",sep="\t",header=T,check.names=F,row.names = 1)
inputFile="TP53.txt"

data=read.table(inputFile,header=T,sep="\t",check.names=F,row.names = 1)
data = merge(os,data,by=0)
```

```
rt = data[data$TP53 == "WT",]
rt = rt[,-c(1,5)]
outTab = c()

cox <- coxph(Surv(futime, fustat) ~ rt[,1], data = rt)
coxSummary = summary(cox)
coxP=coxSummary$coefficients[,"Pr(>|z|)"]
outTab=rbind(outTab,
             cbind(id="WT",
                   HR=coxSummary$conf.int[,"exp(coef)"],
                   HR.95L=coxSummary$conf.int[,"lower .95"],
                   HR.95H=coxSummary$conf.int[,"upper .95"],
                   pvalue=coxSummary$coefficients[,"Pr(>|z|)"])
)
A = outTab



rt = data[data$TP53 == "Mut",]
rt = rt[,-c(1,5)]
outTab = c()

cox <- coxph(Surv(futime, fustat) ~ rt[,1], data = rt)
coxSummary = summary(cox)
coxP=coxSummary$coefficients[,"Pr(>|z|)"]
outTab=rbind(outTab,
             cbind(id="Mut",
                   HR=coxSummary$conf.int[,"exp(coef)"],
                   HR.95L=coxSummary$conf.int[,"lower .95"],
                   HR.95H=coxSummary$conf.int[,"upper .95"],
                   pvalue=coxSummary$coefficients[,"Pr(>|z|)"])
)
rt = rbind(A,outTab)
write.table(rt, file="HR_TP53.txt", sep="\t", quote=F, row.names=F)




inputFile="all.txt"


rt=read.table(inputFile,header=T,sep="\t",row.names=1,check.names=F)
gene=rownames(rt)
hr=sprintf("%.3f",rt$"HR")
hrLow=sprintf("%.3f",rt$"HR.95L")
hrHigh=sprintf("%.3f",rt$"HR.95H")
Hazard.ratio=paste0(hr,"(",hrLow,"-",hrHigh,")")
pVal=ifelse(rt$pvalue<0.001, "<0.001", sprintf("%.3f", rt$pvalue))



n=nrow(rt)
nRow=n+1
ylim=c(1,nRow)
layout(matrix(c(1,2),nc=2),width=c(3,2))


xlim = c(0,3)
```

```
par(mar=c(4,2,1.5,1.5))
plot(1,xlim=xlim,ylim=ylim,type="n",axes=F,xlab="",ylab="")
text.cex=0.8
text(0,n:1,gene,adj=0,cex=text.cex)
text(1.5-0.5*0.2,n:1,pVal,adj=1,cex=text.cex);text(1.5-
0.5*0.2,n+1,'pvalue',cex=text.cex,font=2,adj=1)
text(3,n:1,Hazard.ratio,adj=1,cex=text.cex);text(3,n+1,'Hazard
ratio',cex=text.cex,font=2,adj=1,)


par(mar=c(4,1,1.5,1),mgp=c(2,0.5,0))
xlim = c(0,max(as.numeric(hrLow),as.numeric(hrHigh)))
plot(1,xlim=xlim,ylim=ylim,type="n",axes=F,ylab="",xaxs="i",xlab="Hazard
ratio")
arrows(as.numeric(hrLow),n:1,as.numeric(hrHigh),n:1,angle=90,code=3,lengt
h=0.03,col="darkblue",lwd=2.5)
abline(v=1,col="black",lty=2,lwd=2)
boxcolor = ifelse(as.numeric(hr) > 1, 'red', 'blue')
points(as.numeric(hr), n:1, pch = 15, col = boxcolor, cex=1.3)
axis(1)



#C----
data = read.table("ACC.txt",sep="\t",header=T,check.names=F)

ggplot(data, aes(x = x, y = CRDscore , color = group)) +
  geom_point(aes(colour = factor(group))) +
  geom_hline(aes(yintercept = 0.01574548, group = cancer), colour =
'grey',size = 1,linetype = "dashed")+
  facet_wrap(. ~ cancer, ncol = 33) +
  geom_segment(data = out1, aes(x = ((min(data$x) + max(data$x)) / 2) -
2.5, y = median_y,
                               xend = ((min(data$x) + max(data$x)) / 2)
+ 2.5, yend = median_y),
               linetype = "solid", size = 1.5, color = "black") +
  theme(legend.position = 'none',
        axis.text.x=element_blank(),
        axis.ticks.x = element_blank())+
  labs( y = "Rloopscore" ,x="")



#D----
moduleLabels=read.table("moduleLabels.txt",sep="\t",header=T,check.names=
F,row.names = 1)

library(CRDscore)
samples=list.files("./")
out = file.path('./',samples)

for(i in out){

TCGA=read.table(paste0(i,"//symbol.txt"),sep="\t",header=T,check.names=F)


  rt=as.matrix(TCGA)
  rownames(rt)=rt[,1]
  exp=rt[,2:ncol(rt)]
  dimnames=list(rownames(exp),colnames(exp))
```

```
  mat=matrix(as.numeric(as.matrix(exp)),nrow=nrow(exp),dimnames=dimnames)
  mat=avereps(mat)
  mat=mat[rowMeans(mat)>0.5,]
  mat=log2(mat+1)
  colnames(mat) = gsub("\\.",replacement = "-",x=colnames(mat))


  group=sapply(strsplit(colnames(mat),"\\-"), "[", 4)
  group=sapply(strsplit(group,""), "[", 1)
  group=gsub("2", "1", group)

  Tumor = mat[,group == 0]
  Normal = mat[,group == 1]

  CRDscore_Tumor <- cal_CRDscore(expr = Tumor, n.bins = 50, circadians =
rownames(moduleLabels), study.type = "bulk_RNAseq")
  CRDscore_Normal <- cal_CRDscore(expr = Normal, n.bins = 50, circadians
= rownames(moduleLabels), study.type = "bulk_RNAseq")
  CRDscore_Tumor = as.data.frame(CRDscore_Tumor)
  CRDscore_Normal = as.data.frame(CRDscore_Normal)
  colnames(CRDscore_Tumor)[1]="Rloopscore"
  colnames(CRDscore_Normal)[1]="Rloopscore"

  conNum=length(group[group==1])
  treatNum=length(group[group==0])
  Type=c(rep(1,conNum), rep(2,treatNum))
  data = rbind(CRDscore_Normal,CRDscore_Tumor)
  data$Rloopscore <- (data$Rloopscore-
min(data$Rloopscore))/(max(data$Rloopscore)-min(data$Rloopscore))
  data = t(data)


  outTab=data.frame()
  for(i in row.names(data)){
    rt=data.frame(expression=data[i,], Type=Type)
    wilcoxTest=wilcox.test(expression ~ Type, data=rt)
    conGeneMeans=mean(data[i,1:conNum])
    treatGeneMeans=mean(data[i,(conNum+1):ncol(data)])
    logFC=log2(treatGeneMeans)-log2(conGeneMeans)
    pvalue=wilcoxTest$p.value
    conMed=median(data[i,1:conNum])
    treatMed=median(data[i,(conNum+1):ncol(data)])
    diffMed=treatMed-conMed

outTab=rbind(outTab,cbind(gene=i,conMean=conGeneMeans,treatMean=treatGene
Means,logFC=logFC,pValue=pvalue))

  }
  pValue=outTab[,"pValue"]
  fdr=p.adjust(as.numeric(as.vector(pValue)), method="fdr")
  outTab=cbind(outTab, fdr=fdr)
}

put = c()
score = matrix(data="na", nrow = 3, ncol = 0)
for(i in out){
  print(i)

TCGA=read.table(paste0(i,"//symbol.txt"),sep="\t",header=T,check.names=F)
```

```
  rt=as.matrix(TCGA)
  rownames(rt)=rt[,1]
  exp=rt[,2:ncol(rt)]
  dimnames=list(rownames(exp),colnames(exp))
  mat=matrix(as.numeric(as.matrix(exp)),nrow=nrow(exp),dimnames=dimnames)
  mat=avereps(mat)
  mat=mat[rowMeans(mat)>0.5,]
  mat=log2(mat+1)
  colnames(mat) = gsub("\\.",replacement = "-",x=colnames(mat))


  group=sapply(strsplit(colnames(mat),"\\-"), "[", 4)
  group=sapply(strsplit(group,""), "[", 1)
  group=gsub("2", "1", group)

  Tumor = mat[,group == 0]
  Normal = mat[,group == 1]

  A = as.data.frame(colnames(mat))
  B = substr(colnames(Normal),1,12)

  sample = c()
  for (j in B) {
    C <- as.data.frame(grep(j, A$`colnames(mat)`,value = T))
    sample = rbind(sample,C)
  }

  mat1 = mat[,sample[,1]]

  CRDscore <- cal_CRDscore(expr = mat1, n.bins = 50, circadians =
rownames(moduleLabels), study.type = "bulk_RNAseq")
  CRDscore = as.data.frame(CRDscore)

  group=sapply(strsplit(rownames(CRDscore),"\\-"), "[", 4)
  group=sapply(strsplit(group,""), "[", 1)
  group=gsub("2", "1", group)

  conNum=length(group[group==1])
  treatNum=length(group[group==0])
  Type=c(rep(1,conNum), rep(2,treatNum))

  data = t(CRDscore)
  Tum = as.data.frame(data[,group == 0])
  Nor = as.data.frame(data[,group == 1])
  colnames(Tum)=i
  colnames(Nor)=i
  data1 = rbind(Nor,Tum)
  data = t(data1)

  med_T = sum(Tum)
  med_N = sum(Nor)
  all = as.data.frame(c(i,med_N,med_T))

  outTab=data.frame()
  for(j in row.names(data)){
    rt=data.frame(expression=data[j,], Type=Type)
    wilcoxTest=wilcox.test(expression ~ Type, data=rt)
    conGeneMeans=mean(data[j,1:conNum])
    treatGeneMeans=mean(data[j,(conNum+1):ncol(data)])
    logFC=log2(treatGeneMeans)-log2(conGeneMeans)
```

```
    pvalue=wilcoxTest$p.value
    conMed=median(data[j,1:conNum])
    treatMed=median(data[j,(conNum+1):ncol(data)])
    diffMed=treatMed-conMed

outTab=rbind(outTab,cbind(gene=j,conMean=conGeneMeans,treatMean=treatGene
Means,logFC=logFC,pValue=pvalue))

  }
  pValue=outTab[,"pValue"]
  fdr=p.adjust(as.numeric(as.vector(pValue)), method="fdr")
  outTab=cbind(outTab, fdr=fdr)

  put = rbind(put,outTab)
  score = cbind(score,all)
}

write.table(put, file="p.txt", sep="\t", quote=F, row.names=F)
write.table(score, file="score.txt", sep="\t", quote=F, col.names=F)


rt=read.table("relust.txt", header=T, sep="\t", check.names=F)
rt[,"P"]=ifelse(rt$fdr<= 0.05, 5, 1)
rt = arrange(rt,desc(score))
rt = arrange(rt,Type)
rt$cancer <- factor(rt$cancer,levels=rt$cancer[1:22])
rt$cancer <-
factor(rt$cancer,levels=c("LUAD","LIHC","COAD","KIRC","HNSC","PRAD","UCEC
","KIRP","ESCA",

"SARC","BLCA","BRCA","LUSC","THYM"))

ggplot(rt,aes(x=cancer,y=Type,size=P,color=score)) +
  geom_point() + theme_bw() +
  scale_colour_gradientn(colours =
c("#063061","#4293c1","white","#d6604d","#65001e"))+
  labs(x="",y="",title="",
       colour="Rloopscore") +
  theme(plot.title = element_text(hjust = 0.5))+
  theme(panel.grid.major =element_blank(),
        panel.grid.minor = element_blank(),
        panel.background = element_blank(),
        panel.border = element_blank())





#E----
setwd("D:\\LY\\5.3\\work\\Lung\\TCGA\\cli")
library(data.table)
samples=list.files("./")
out = file.path('./',samples)

#i = ".//TCGA-BLCA.GDC_phenotype.tsv.gz"

data = c()
for (i in out) {
  rt = fread(i,sep="\t",header=T,check.names=F)
```

```
  A =
rt[,c("submitter_id.samples","age_at_initial_pathologic_diagnosis","gende
r.demographic")]
  data = rbind(A,data)
}
colnames(data) = c("id","Age","Sex")
data[,"Sex"]=ifelse(data[,"Sex"] != "   female", 1, 0)
setwd("D:\\LY\\5.3\\work\\Lung\\5.26\\fig5\\K")
write.table(data, file="data.txt", sep="\t", quote=F, row.names=F)


library(survival)
library(tableone)

library(forestplot)
library(stringr)
Cox=read.table("data.txt", header=T, sep="\t", check.names=F,row.names =
1)
Cox = na.omit(Cox)


setwd("D:\\LY\\5.3\\work\\Lung\\TCGA\\1.1")
samples=list.files("./")
out = file.path('./',samples)


A = c()
B = c()
for(j in out){
  print(j)
  CRDscore = read.table(paste0(j,"//CRDscore-
cutoff.txt"),sep="\t",header=T,check.names=F,row.names = 1)
  rownames(CRDscore) = CRDscore[,1]
  rt = CRDscore[,2:4]
  outTab=data.frame()

  cox <- coxph(Surv(futime, fustat) ~ rt[,3], data = rt)
  coxSummary = summary(cox)
  coxP=coxSummary$coefficients[,"Pr(>|z|)"]
  outTab=rbind(outTab,
               cbind(id=j,
                     HR=coxSummary$conf.int[,"exp(coef)"],
                     HR.95L=coxSummary$conf.int[,"lower .95"],
                     HR.95H=coxSummary$conf.int[,"upper .95"],
                     pvalue=coxSummary$coefficients[,"Pr(>|z|)"])
  )

  A = rbind(A,outTab)
  #write.table(outTab, file=paste0(i,"//CRDscore-cutoff.txt"), sep="\t",
quote=F, col.names=F)

  exp = merge(rt,Cox,by=0)
  exp = exp[,-1]

  allOutTab=data.frame()
  sigGenes=c("futime","fustat")


  multiCox=coxph(Surv(futime, fustat) ~ ., data = exp)#
  coxSummary=summary(multiCox)
```

```
  allOutTab=rbind(
    cbind(id=j,
          HR=coxSummary$conf.int["CRDscore","exp(coef)"],
          HR.95L=coxSummary$conf.int["CRDscore","lower .95"],
          HR.95H=coxSummary$conf.int["CRDscore","upper .95"],
          pvalue=coxSummary$coefficients["CRDscore","Pr(>|z|)"])
  )


  B = rbind(B,allOutTab)



}

write.table(A, file="Univariate_Cox.txt", quote=F, sep="\t",row.names =
F)
write.table(B, file="Multivariate_Cox.txt", quote=F, sep="\t",row.names =
F)


rt=read.table("relust.txt", header=T, sep="\t", check.names=F)
rt$P = ifelse(rt$pvalue < 0.001 ,"2","1")

ggplot(rt,aes(x=cancer,y=Type,size=group,color=HR1)) +
  geom_point(shape=15) + theme_bw() +
  scale_colour_gradient(low="#f5ef5f",high="#4a68b1") +
  labs(x="",y="",title="") +
  theme(plot.title = element_text(hjust = 0.5))+
  theme(panel.grid.major =element_blank(),
        panel.grid.minor = element_blank(),
        panel.background = element_blank(),
        panel.border = element_blank())





#fig6----
#A-F----
moduleLabels=read.table("rloop.txt", header=T, sep="\t", check.names=F,
row.names=1)
rt=read.table("GSE25055_exp.txt", header=T, sep="\t",
check.names=F,row.names = 1)
os=read.table("os.txt", header=T, sep="\t", check.names=F,row.names = 1)

exp = t(rt)
same = intersect(rownames(moduleLabels),colnames(exp))
exp = exp[,same]
exp = merge(os,exp,by=0)

allOutTab=data.frame()
sigGenes=c("futime","fustat")
exp = exp[,-1]
for(M in colnames(exp[,3:ncol(exp)])){
  rt1=cbind(exp[,1:2],gene=exp[,M])
  colnames(rt1)[ncol(rt1)]=M
```

```
  cox <- coxph(Surv(futime, fustat) ~ rt1[,ncol(rt1)], data = rt1)
  coxSummary = summary(cox)
  outTab=data.frame()
  if(coxSummary$coefficients[,"Pr(>|z|)"]<0.05){
    sigGenes=c(sigGenes,M)
    allOutTab=rbind(allOutTab,
                    cbind(id=M,
                          HR=coxSummary$conf.int[,"exp(coef)"],
                          HR.95L=coxSummary$conf.int[,"lower .95"],
                          HR.95H=coxSummary$conf.int[,"upper .95"],
                          pvalue=coxSummary$coefficients[,"Pr(>|z|)"])
    )
  }
}

write.table(allOutTab, file="allOutTab.txt", quote=F, sep="\t",row.names
= F )
allOutTab = allOutTab[allOutTab$HR > 1,]
CRDscore <- cal_CRDscore(expr = rt, n.bins = 50, circadians =
allOutTab[,1], study.type = "bulk_RNAseq")
A = as.data.frame(CRDscore)

rt1 = merge(os,A,by=0)

var="CRDscore"
rt1=rt1[,c(2,3,4)]


res.cut=surv_cutpoint(rt1, time="futime", event="fustat",
variables=c("CRDscore"))
cutoff=as.numeric(res.cut$cutpoint[1])
print(cutoff)
group=ifelse(rt1[,"CRDscore"]<=cutoff, "Low", "High")



diff=survdiff(Surv(futime, fustat) ~group,data = rt1)
pValue=1-pchisq(diff$chisq,df=1)
if(pValue<0.001){
  pValue="p<0.001"
}else{
  pValue=paste0("p=",sprintf("%.03f",pValue))
}
fit <- survfit(Surv(futime, fustat) ~ group, data = rt1)

ggsurvplot(
  fit,
  pval = TRUE,
  pval.method = TRUE,
  pval.size=5,
  legend.title='Rloopscore',
  legend.labs=c('High','Low'),
  conf.int = TRUE,
  linetype = "strata",
  xlab="Time(years)",
  break.time.by = 2,
  surv.median.line = "hv",
  ggtheme = theme_bw(),
  palette = c("#E31A1C","#1F78B4")
)
```

```
Re=read.table("response.txt", header=T, sep="\t", check.names=F,row.names
= 1)
rt1 = merge(Re,A,by=0)
rt1 = na.omit(rt1)

group=levels(factor(rt1$Type))
rt1$Type=factor(rt1$Type, levels=group)
comp=combn(group,2)
my_comparisons=list()
for(i in 1:ncol(comp)){my_comparisons[[i]]<-comp[,i]}

ggplot(data=rt1,aes(x = Type, y=CRDscore, color=Type))+
  stat_boxplot(geom="errorbar",width=0.5,size=1.3)+
  geom_boxplot(alpha=1,outlier.shape = NA,size=1.3,width=0.5,fatten=1)+
  theme_bw()+
  theme(panel.grid=element_blank())+
  stat_compare_means(comparisons = my_comparisons,method="wilcox.test")+
  theme(legend.position = "right")+
  ggtitle("") +
  xlab("")+
  ylab("Rloopscore")+
  scale_color_manual(values=c("#00AFBB", "#E7B800"))+
  theme(
    axis.title.x=element_blank(),
    axis.text.y = element_text(size = 15,face = "bold",color = "black"),
    axis.title.y = element_text(size = 15,face = "bold",color = "black"),
    axis.text.x = element_text(size = 15,face = "bold",color = "black"),
    legend.position = "none",

    plot.title = element_text(face = "bold",size=15,hjust = 0.5))




#G----
samples=list.files("./")
out = file.path('./',samples)

auc = c()
GSE = c()
for (i in out) {
  j = substr(i,4,19)

exp=read.table(paste0(i,"//",j,"_exp.txt"),sep="\t",header=T,check.names=
F,row.names = 1)
  Re =
read.table(paste0(i,"//response.txt"),sep="\t",header=T,check.names=F,row
.names = 1)
  Rloop =
read.table(paste0(i,"//cox.txt"),sep="\t",header=T,check.names=F,row.name
s = 1)
  Rloop = Rloop[Rloop$HR > 1,]

  score = cal_CRDscore(expr = exp, n.bins = 50, circadians =
rownames(Rloop), study.type = "bulk_RNAseq")
```

```
  score = as.data.frame(score)
  rt = merge(Re,score,by=0)
  rocobj1=roc(rt[,2], as.vector(rt[,3]))
  auc = c(auc,rocobj1[["auc"]])
  GSE = c(GSE,j)
}
A = data.frame(GSE,auc)
write.table(A, file="all.txt", quote=F, sep="\t",row.names = F )

rt=read.table("all.txt",sep="\t",header=T,check.names=F)
colnames(rt)[1]="id"

ggplot(rt, aes(x = reorder(id,auc),y=auc,fill=cancer))+
  geom_bar(stat = "identity")+
  labs(x="",y="AUC")+theme_classic() +
  theme(axis.text.x = element_text(angle = 60, hjust = 1))+
  scale_y_continuous(limits = c(0, 1))+
  geom_hline(aes(yintercept=0.5), colour="#990000",
linetype="dashed",size=2)




#fig7----
#C----
library(ggpubr)
dat=read.table("LUAD.TPM.txt",header=T,sep="\t",check.names=F)
rt=as.matrix(dat)
rownames(rt)=rt[,1]
exp=rt[,2:ncol(rt)]
dimnames=list(rownames(exp), colnames(exp))
data=matrix(as.numeric(as.matrix(exp)), nrow=nrow(exp),
dimnames=dimnames)
data=avereps(data)
data=data[rowMeans(data)>0,]

A = as.data.frame(data["FANCI",])
A = cbind(id=rownames(A),A)
A$id = substr(as.character(A$id),14,15)
A$Type = ifelse(A$id != "11","Tumor","Normal")


group=levels(factor(A$Type))
A$Type=factor(A$Type, levels=group)
comp=combn(group,2)
my_comparisons=list()
for(i in 1:ncol(comp)){my_comparisons[[i]]<-comp[,i]}
colnames(A)[2] = "CRDscore"

p1=ggplot(data=A,aes(x = Type, y=CRDscore, color=Type))+
  stat_boxplot(geom="errorbar",width=0.5,size=1.3)+
  geom_boxplot(alpha=1,outlier.shape = NA,size=1.3,width=0.5,fatten=1)+
  theme_bw()+
  theme(panel.grid=element_blank())+
  stat_compare_means(comparisons = my_comparisons,method="wilcox.test")+
  theme(legend.position = "right")+
  ggtitle("") +
  xlab("")+
  ylab("Expression")+
  scale_color_manual(values=c("#00AFBB", "#E7B800"))+
```

```
  theme(
    axis.title.x=element_blank(),
    axis.text.y = element_text(size = 15,face = "bold",color = "black"),
    axis.title.y = element_text(size = 15,face = "bold",color = "black"),
    axis.text.x = element_text(size = 15,face = "bold",color = "black"),
    legend.position = "none",
    plot.title = element_text(face = "bold",size=15,hjust = 0.5))

dat=read.table("LUAD.TPM.txt",header=T,sep="\t",row.names=1,check.names=F
)
os=read.table("os.txt",header=T,sep="\t",row.names=1,check.names=F)
colnames(dat) = substr(as.character(colnames(dat)),1,16)
A = as.data.frame(t(dat["FANCI",]))
rownames(A) = gsub(".",replacement = "-",rownames(A),fixed = T)

rt1 = merge(A,os,by=0)

var="FANCI"
rt1$futime = rt1$futime/365

res.cut=surv_cutpoint(rt1, time="futime", event="fustat",
variables=c("FANCI"))
cutoff=as.numeric(res.cut$cutpoint[1])
print(cutoff)
group=ifelse(rt1[,"FANCI"]<=cutoff, "Low", "High")


diff=survdiff(Surv(futime, fustat) ~group,data = rt1)
pValue=1-pchisq(diff$chisq,df=1)
if(pValue<0.001){
  pValue="p<0.001"
}else{
  pValue=paste0("p=",sprintf("%.03f",pValue))
}
fit <- survfit(Surv(futime, fustat) ~ group, data = rt1)

p=ggsurvplot(
  fit, #
  pval = TRUE, #
  pval.method = TRUE,
  pval.size=5,#
  legend.title='FANCI',
  legend.labs=c('High','Low'),
  conf.int = TRUE,
  risk.table = TRUE,
  linetype = "strata",
  xlab="Time(years)",
  break.time.by = 2,
  surv.median.line = "hv",
  ggtheme = theme_bw(),
  palette = c("#E31A1C","#1F78B4")
)
p

#----

#S1----
#A----
library(ggstatsplot)
library(RColorBrewer)
```

```
load("scRNA.Rdata")
cli=read.table("cli.txt", header=T, sep="\t", check.names=F)
A = scRNA@meta.data

j=0
for (i in cli[,2]) {
  j=j+1
  A$orig.ident[which(A$orig.ident == i)] <- cli[j,1]
}

B = as.data.frame(table(A$orig.ident))

data("mtcars")

col = c("#EFF3FF" ,"#E2EBF9" ,"#D6E4F3" ,"#C9DEED" ,"#BDD7E7" ,"#A8CCE2"
,"#94C2DE" ,"#7FB8DA","#6BAED6",
        "#5CA3CF", "#4E98C9", "#3F8DC3", "#3182BD", "#2675B4", "#1C69AC"
,"#125DA4", "#08519C",
        "#FFFFD4", "#FEEEB4", "#FEDD95", "#FEC36C", "#FEA73F", "#F58C23",
"#E57217", "#D15A0C", "#B54708","#993404",
        "#F2F0F7", "#9E9AC8", "#54278F")


A %>% mutate(orig.ident = factor(orig.ident, levels =
c("C3","C2","C1","M10","M9","M8","M7","M6","M5","M4","M3","M2","M1","P17"
,"P16",

"P15","P14","P13","P12","P11","P10","P9","P8","P7","P6","P5","P4","P3","P
2","P1") )) ->A

View(paletteer::palettes_d_names)

p5=ggpiestats(A, "orig.ident",
              results.subtitle = F,


              perc.k = 2,
              direction = 1,
              package = "ggsci",
              palette = 'alternating_igv',
              title = '')
```

```
#B----
load("scRNA.Rdata")

ElbowPlot(scRNA, ndims=50, reduction="pca")
```

```
#C----
load("scRNA.Rdata")

DimPlot(scRNA, reduction = "tsne",label = TRUE)
DimPlot(scRNA, reduction = "umap",label = F)



#D----
load("scRNA.Rdata")

select_genes <- c("IGHA2","IGHG3","IGHM","CD79A",
                  "CD83","CD141","CD1A","CD1C",
                  "RAMP2","FLT1","CLDN5","PECAM1",
                  "KRT18","CDH1","KRT19","EPCAM",
                  "THY1","COL1A2","COL1A1","DCN",
                  "LYZ","FCGR3A","MARCO","CD68",
                  "GATA2","MS4A2","KIT",
                  "KLRD1","GNLY","NKG7","NCAM1",
                  "OLIG2","CNP","FA2H","MBP",
                  "TRAC","CD3G","CD3E","CD3D")
DotPlot(scRNA, features = select_genes )+ coord_flip()+
  scale_y_discrete(limits = c(
    "0","1","3","5","9","12","13","15","24","45","51","53","55","56",
    "37",
    "7","38",
    "21","44","46",
    "2","6","11","14","22","42",
    "17","43",

"8","16","18","19","20","25","26","28","29","30","31","32","35","36","39"
,"40","41","48","49","50",
    "33",
    "10",
    "4","23","27","34","47","54"
  ))





#E----

load("scRNA.Rdata")
library(infercnv)

exp = as.data.frame(scRNA@assays[["RNA"]]@counts)
exp = cbind(gene = rownames(exp),exp)
write.table(exp, file="raw_counts_matrix.txt", sep="\t", quote=F,
row.names=F)


ann = scRNA@meta.data[3]
ann1 = cbind(id=rownames(ann),ann)
write.table(ann1, file="annotations_file.txt", sep="\t", quote=F,
row.names=F,col.names = F)

infercnv_obj =
CreateInfercnvObject(raw_counts_matrix="raw_counts_matrix.txt",
```

```
annotations_file="annotations_file.txt",
                                    delim="\t",

gene_order_file="gene_order_file.txt",
                                    ref_group_names=NULL)

# perform infercnv operations to reveal cnv signal
infercnv_obj = infercnv::run(infercnv_obj,
                             cutoff=0.1,  # use 1 for smart-seq, 0.1 for
10x-genomics
                             out_dir="output_dir1",  # dir is auto-
created for storing outputs
                             cluster_by_groups=T,   # cluster
                             denoise=T,
                             HMM=F
)


#F----

rt =
read.table("ECscore_Malignant.txt",sep="\t",header=T,check.names=F,row.na
mes = 1)
rt = rt[,c(2,3)]
x=colnames(rt)[1]
y=colnames(rt)[2]
colnames(rt)=c("Expression","cancer")


group=levels(factor(rt$cancer))
rt$cancer=factor(rt$cancer, levels=group)
comp=combn(group,2)
my_comparisons=list()
for(i in 1:ncol(comp)){my_comparisons[[i]]<-comp[,i]}


ggplot(data=rt,aes(x =reorder(cancer,Expression,  FUN = median),
y=Expression, color=cancer))+
  stat_boxplot(geom="errorbar",width=0.5,size=1.3)+
  geom_boxplot(alpha=1,outlier.shape = NA,size=1.3,width=0.5,fatten=1)+
  theme_bw()+
  theme(panel.grid=element_blank())+
  theme(legend.position = "right")+
  ggtitle("") +
  xlab("")+
  ylab("Epithelial Score")+
  rotate_x_text(60)+
  scale_color_manual(values=c("#00AFBB", "#E7B800"))+
  theme(
    axis.title.x=element_blank(),
    axis.text.y = element_text(size = 15,face = "bold",color = "black"),
    axis.title.y = element_text(size = 15,face = "bold",color = "black"),
    axis.text.x = element_text(size = 15,face = "bold",color = "black"),
    legend.position = "none",

    plot.title = element_text(face = "bold",size=15,hjust = 0.5))
```

```
#H----

library("clusterProfiler")
library("org.Hs.eg.db")
library("enrichplot")
library("ggplot2")

pvalueFilter=0.05
qvalueFilter=0.05


colorSel="qvalue"
if(qvalueFilter>0.05){
  colorSel="pvalue"
}

moduleLabels=read.table("moduleLabels.txt", header=T, sep="\t",
check.names=F, row.names=1)
rt = moduleLabels[moduleLabels$moduleLabels == "9" |
moduleLabels$moduleLabels == "5" |
                    moduleLabels$moduleLabels == "10" |
moduleLabels$moduleLabels == "8",]


genes=unique(as.vector(rownames(rt)))
entrezIDs=mget(genes, org.Hs.egSYMBOL2EG, ifnotfound=NA)
entrezIDs=as.character(entrezIDs)
gene=entrezIDs[entrezIDs!="NA"]


kk=enrichGO(gene=gene, OrgDb=org.Hs.eg.db, pvalueCutoff=1,
qvalueCutoff=1, ont="BP", readable=T)
GO=as.data.frame(kk)
GO=GO[(GO$pvalue<pvalueFilter & GO$qvalue<qvalueFilter),]


rt = GO

colnames(rt)
labels=rt[order(rt$GeneRatio),"Description"]
rt$Description = factor(rt$Description,levels=labels)


p = ggplot(rt,aes(GeneRatio, Description)) +
  geom_point(aes(size=Count, color=p.adjust))
p1 = p +
  scale_colour_gradient(low="red",high="blue") +
  labs(color="FDR",size="Count",x="Gene ratio",y="Term")+
  theme(axis.text.x=element_text(color="black",
size=10),axis.text.y=element_text(color="black", size=10)) +
  theme_bw()
p1





#I----
```

```
load("Malignant.Rdata")
DimPlot(pbmc1, reduction = "umap",label = F,group.by = "Origin")


#J----
ann = read.table("ann.txt",sep="\t",header=T,check.names=F,row.names = 1)
score =
read.table("All_score.txt",sep="\t",header=T,check.names=F,row.names = 1)
rt = merge(ann,score,by=0)

rt = rt[,c(4,3)]
x=colnames(rt)[1]
y=colnames(rt)[2]
colnames(rt)=c("Expression","cancer")

rt$cancer[which(rt$cancer =='B_cell')] <- "Immune Cell"
rt$cancer[which(rt$cancer =='DC')] <- "Immune Cell"
rt$cancer[which(rt$cancer =='Macrophage')] <- "Immune Cell"
rt$cancer[which(rt$cancer =='Mast')] <- "Immune Cell"
rt$cancer[which(rt$cancer =='NK_cell')] <- "Immune Cell"
rt$cancer[which(rt$cancer =='T_cells')] <- "Immune Cell"

group=levels(factor(rt$cancer))
rt$cancer=factor(rt$cancer, levels=group)
comp=combn(group,2)
my_comparisons=list()
for(i in 1:ncol(comp)){my_comparisons[[i]]<-comp[,i]}

ggplot(data=rt,aes(x =reorder(cancer,Expression,  FUN = median),
y=Expression, color=cancer))+
  stat_boxplot(geom="errorbar",width=0.5,size=1.3)+
  geom_boxplot(alpha=1,outlier.shape = NA,size=1.3,width=0.5,fatten=1)+
  theme_bw()+
  theme(panel.grid=element_blank())+
  theme(legend.position = "right")+
  ggtitle("") +
  xlab("")+
  ylab("RloopScore")+
  scale_y_continuous(limits = c(-1, 1))+
  rotate_x_text(60)+
  theme(
    axis.title.x=element_blank(),
    axis.text.y = element_text(size = 15,face = "bold",color = "black"),
    axis.title.y = element_text(size = 15,face = "bold",color = "black"),
    axis.text.x = element_text(size = 15,face = "bold",color = "black"),
    legend.position = "none",
    plot.title = element_text(face = "bold",size=15,hjust = 0.5))




#K----
load("Malignant.Rdata")
gene = read.table("gene.txt",sep="\t",header=T,check.names=F,row.names =
1)
cluster =
read.table("pbmc@meta.data.txt",sep="\t",header=T,check.names=F,row.names
= 1)
same = intersect(rownames(gene),rownames(pbmc1))
```

```
A = pbmc1[same,]
exp = as.data.frame(A@assays[["RNA"]]@data)
rt = t(exp)

data = merge(cluster,rt,by=0)

A <- data[,-c(1:8)]
colnames(A)[1] = "Type"
A=melt(A,id.vars=c("Type"))
colnames(A)=c("Type","Gene","Expression")
A[,3] = as.numeric(A[,3])


p=ggplot(data=A,aes(x = Gene, y=Expression, color=Type))+
  scale_color_manual(values=c("#00AFBB", "#E7B800"))+
  geom_boxplot(aes(color=Type),
               position=position_dodge(0.8),
               alpha=1,outlier.shape = NA,size=1.3,width=0.5,fatten=1,
               outlier.color = "white")+
  stat_compare_means(aes(group=Type),
                     method="t.test",
                     symnum.args=list(cutpoints = c(0, 0.001, 0.01, 0.05,
1), symbols = c("***", "**", "*", " ")),
                     label = "p.signif")+
  theme_bw()+
  theme(legend.position = "top",
        plot.title = element_text(hjust = 0.5),
        axis.text.x = element_text(angle = 40, hjust = 1))+
  theme(panel.grid.major =element_blank(),
        panel.grid.minor = element_blank(),
        panel.background = element_blank(),
        panel.border = element_blank())



load("Malignant.Rdata")
ms =
read.table("pbmc@meta.data.txt",sep="\t",header=T,check.names=F,row.names
= 1)
ms = ms[colnames(pbmc1),]
pbmc1@meta.data$Type = ms$group

s.genes <- cc.genes$s.genes
g2m.genes <- cc.genes$g2m.genes

pbmc3k <- CellCycleScoring(pbmc1, s.features = s.genes, g2m.features =
g2m.genes, set.ident = TRUE)
A = pbmc3k@meta.data

a <- data.frame(table(A$Type,A$Phase))
a<- ddply(a,.(Var1),transform,percent=Freq/sum(Freq)*100)
a$label = paste0(sprintf("%.1f", a$percent), "%")

a %>%
  drop_na() %>%
  ggplot(aes(fill=Var2, y= percent, x = Var1)) +
  geom_bar(position="fill", stat = "identity") +
  scale_fill_manual(values = c("#b3de69","#80b1d5","#bebada"))+
  scale_y_continuous(labels = scales::percent) +
  ylab("Percent(%)")+xlab("")+labs(fill="")+
```

```
  theme_gray()+
  theme(legend.position = "top",
        panel.grid.major =element_blank(),
        panel.grid.minor = element_blank(),
        panel.background = element_blank(),
        panel.border = element_blank()
  )



#S2----
#A----
dat=read.table("LUAD.TPM.txt",header=T,sep="\t",row.names=1,check.names=F
)
dat=log2(edgeR::cpm(exprSet)+1)
library(estimate)
estimate <- function(dat,pro){
  input.f=paste0(pro,'_estimate_input.txt')
  output.f=paste0(pro,'_estimate_gene.gct')
  output.ds=paste0(pro,'_estimate_score.gct')
  write.table(dat,file = input.f,sep = '\t',quote = F)
  library(estimate)
  filterCommonGenes(input.f=input.f,
                    output.f=output.f ,
                    id="GeneSymbol")
  estimateScore(input.ds = output.f,
                output.ds=output.ds,
                platform="illumina")
  scores=read.table(output.ds,skip = 2,header = T)
  rownames(scores)=scores[,1]
  scores=t(scores[,3:ncol(scores)])
  return(scores)
}
pro='LUAD'
scores=estimate(dat,pro)

TumorPurity = cos(0.6049872018+0.0001467884 * scores[,3])
scores1 = cbind(scores,TumorPurity)
scores1 = cbind(id=rownames(scores1),scores1)
write.table(scores1,file="ESTMATEScore.txt",sep="\t",quote=F,row.names=F)




rloopscore=read.table("CRDscore.txt",header=T,sep="\t",row.names=1,check.
names=F)
ES=read.table("ESTMATEScore.txt",header=T,sep="\t",row.names=1,check.name
s=F)

rt = merge(rloopscore,ES,by=0)
rt$Type = ifelse(rt$CRDscore <= median(rt$CRDscore), "Low", "High")

rt1 = rt[,c(5,9)]
colnames(rt1)=c("Expression","cancer")
rt1$cancer = as.character(rt1$cancer)

group=levels(factor(rt1$cancer))
rt1$cancer=factor(rt1$cancer, levels=group)
comp=combn(group,2)
my_comparisons=list()
```

```
for(i in 1:ncol(comp)){my_comparisons[[i]]<-comp[,i]}

p1=ggplot(data=rt1,aes(x =reorder(cancer,Expression,  FUN = median),
y=Expression, color=cancer))+
  stat_boxplot(geom="errorbar",width=0.5,size=1.3)+
  geom_boxplot(alpha=1,outlier.shape = NA,size=1.3,width=0.5,fatten=1)+
  theme_bw()+
  theme(panel.grid=element_blank())+
  stat_compare_means(comparisons =
my_comparisons,symnum.args=list(cutpoints = c(0, 0.001, 0.01, 0.05, 1),
symbols = c("***", "**", "*", "ns")),label = "p.signif")+
  theme(legend.position = "right")+
  ggtitle("") +
  xlab("")+
  ylab("StromalScore")+
  rotate_x_text(60)+
  scale_color_manual(values=c( "#E7B800","#00AFBB"))+
  theme(
    axis.title.x=element_blank(),
    axis.text.y = element_text(size = 15,face = "bold",color = "black"),
    axis.title.y = element_text(size = 15,face = "bold",color = "black"),
    axis.text.x = element_text(size = 15,face = "bold",color = "black"),
    legend.position = "none",
    plot.title = element_text(face = "bold",size=15,hjust = 0.5))



rt1 = rt[,c(6,9)]
colnames(rt1)=c("Expression","cancer")
rt1$cancer = as.character(rt1$cancer)

group=levels(factor(rt1$cancer))
rt1$cancer=factor(rt1$cancer, levels=group)
comp=combn(group,2)
my_comparisons=list()
for(i in 1:ncol(comp)){my_comparisons[[i]]<-comp[,i]}

p1=ggplot(data=rt1,aes(x =reorder(cancer,Expression,  FUN = median),
y=Expression, color=cancer))+
  stat_boxplot(geom="errorbar",width=0.5,size=1.3)+
  geom_boxplot(alpha=1,outlier.shape = NA,size=1.3,width=0.5,fatten=1)+
  theme_bw()+
  theme(panel.grid=element_blank())+
  stat_compare_means(comparisons =
my_comparisons,symnum.args=list(cutpoints = c(0, 0.001, 0.01, 0.05, 1),
symbols = c("***", "**", "*", "ns")),label = "p.signif")+
  theme(legend.position = "right")+
  ggtitle("") +
  xlab("")+
  ylab("ImmuneScore")+
  rotate_x_text(60)+
  scale_color_manual(values=c( "#E7B800","#00AFBB"))+
  theme(
    axis.title.x=element_blank(),
    axis.text.y = element_text(size = 15,face = "bold",color = "black"),
    axis.title.y = element_text(size = 15,face = "bold",color = "black"),
    axis.text.x = element_text(size = 15,face = "bold",color = "black"),
    legend.position = "none",
    plot.title = element_text(face = "bold",size=15,hjust = 0.5))
```

```
rt1 = rt[,c(7,9)]
colnames(rt1)=c("Expression","cancer")
rt1$cancer = as.character(rt1$cancer)

group=levels(factor(rt1$cancer))
rt1$cancer=factor(rt1$cancer, levels=group)
comp=combn(group,2)
my_comparisons=list()
for(i in 1:ncol(comp)){my_comparisons[[i]]<-comp[,i]}

p1=ggplot(data=rt1,aes(x =reorder(cancer,Expression,  FUN = median),
y=Expression, color=cancer))+
  stat_boxplot(geom="errorbar",width=0.5,size=1.3)+
  geom_boxplot(alpha=1,outlier.shape = NA,size=1.3,width=0.5,fatten=1)+
  theme_bw()+
  theme(panel.grid=element_blank())+
  stat_compare_means(comparisons =
my_comparisons,symnum.args=list(cutpoints = c(0, 0.001, 0.01, 0.05, 1),
symbols = c("***", "**", "*", "ns")),label = "p.signif")+
  theme(legend.position = "right")+
  ggtitle("") +
  xlab("")+
  ylab("ESTIMATEScore")+
  rotate_x_text(60)+
  scale_color_manual(values=c( "#E7B800","#00AFBB"))+
  theme(
    axis.title.x=element_blank(),
    axis.text.y = element_text(size = 15,face = "bold",color = "black"),
    axis.title.y = element_text(size = 15,face = "bold",color = "black"),
    axis.text.x = element_text(size = 15,face = "bold",color = "black"),
    legend.position = "none",
    plot.title = element_text(face = "bold",size=15,hjust = 0.5))




rt1 = rt[,c(8,9)]
colnames(rt1)=c("Expression","cancer")
rt1$cancer = as.character(rt1$cancer)


group=levels(factor(rt1$cancer))
rt1$cancer=factor(rt1$cancer, levels=group)
comp=combn(group,2)
my_comparisons=list()
for(i in 1:ncol(comp)){my_comparisons[[i]]<-comp[,i]}

p1=ggplot(data=rt1,aes(x =reorder(cancer,Expression,  FUN = median),
y=Expression, color=cancer))+
  stat_boxplot(geom="errorbar",width=0.5,size=1.3)+
  geom_boxplot(alpha=1,outlier.shape = NA,size=1.3,width=0.5,fatten=1)+
  theme_bw()+
  theme(panel.grid=element_blank())+
  stat_compare_means(comparisons =
my_comparisons,symnum.args=list(cutpoints = c(0, 0.001, 0.01, 0.05, 1),
symbols = c("***", "**", "*", "ns")),label = "p.signif")+
  theme(legend.position = "right")+
```

```
  ggtitle("") +
  xlab("")+
  ylab("TumorPurity")+
  rotate_x_text(60)+
  scale_color_manual(values=c( "#E7B800","#00AFBB"))+
  theme(
    axis.title.x=element_blank(),
    axis.text.y = element_text(size = 15,face = "bold",color = "black"),
    axis.title.y = element_text(size = 15,face = "bold",color = "black"),
    axis.text.x = element_text(size = 15,face = "bold",color = "black"),
    legend.position = "none",
    plot.title = element_text(face = "bold",size=15,hjust = 0.5))




#B----
inputFile="LUAD.TPM.txt"
gmtFile="immune.gmt"


rt=read.table(inputFile,sep="\t",header=T,check.names=F)
rt=as.matrix(rt)
rownames(rt)=rt[,1]
exp=rt[,2:ncol(rt)]
dimnames=list(rownames(exp),colnames(exp))
mat=matrix(as.numeric(as.matrix(exp)),nrow=nrow(exp),dimnames=dimnames)
mat=avereps(mat)
mat=mat[rowMeans(mat)>0,]
rt <- t(mat)
rt <- cbind(id=rownames(rt),rt)

id <- substr(as.character(rt[,1]),1,16)
rt <- data.frame(id,rt)
rt <- rt[,-c(2)]
rt = as.matrix(rt)
rownames(rt)=rt[,1]
rt = rt[,-1]
mat = t(rt)

cluster=read.table("CRDscore.txt",sep="\t",header=T,check.names=F)
sameSample = intersect(cluster[,1], colnames(mat))
mat = mat[,sameSample]

geneSet=getGmt(gmtFile,
               geneIdType=SymbolIdentifier())

exp=mat
dimnames=list(rownames(exp),colnames(exp))
mat=matrix(as.numeric(as.matrix(exp)),nrow=nrow(exp),dimnames=dimnames)

ssgseaScore=gsva(mat, geneSet, method='ssgsea', kcdf='Gaussian',
abs.ranking=TRUE ,min.sz = 10)

normalize=function(x){
  return((x-min(x))/(max(x)-min(x)))}

ssgseaOut=normalize(ssgseaScore)
ssgseaOut=rbind(id=colnames(ssgseaOut),ssgseaOut)
write.table(ssgseaOut,file="ssgseaOut.txt",sep="\t",quote=F,col.names=F)
```

```
rt=read.table("ssgseaOut.txt",sep="\t",header=T,check.names=F,row.names =
1)
cluster=read.table("CRDscore.txt",sep="\t",header=T,check.names=F,row.nam
es = 1)
cluster$Type = ifelse(cluster$CRDscore <= median(cluster$CRDscore),
"Low", "High")

rt = rbind(colnames(rt),rt)
rt = t(rt)

data = merge(cluster,rt,by.x=0,by.y=1)


A <- data[,-c(1:4)]
A=melt(A,id.vars=c("Type"))
colnames(A)=c("Type","Gene","Expression")
A[,3] = as.numeric(A[,3])

p=ggboxplot(A, x="Gene", y="Expression", color = "Type",
            ylab="Immune Infiltration",
            xlab="",
            palette = c("#E7B800","#00AFBB"),
            width=0.6, add = "none")
p=p+rotate_x_text(60)
p1=p+stat_compare_means(aes(group=Type),
                        method="anova",
                        symnum.args=list(cutpoints = c(0, 0.001, 0.01,
0.05, 1), symbols = c("***", "**", "*", "ns")),
                        label = "p.signif")

#C----
inputFile="LUAD.TPM.txt"
library(limma)
library(reshape2)
library(dplyr)


rt=read.table(inputFile,sep="\t",header=T,check.names=F)
rt=as.matrix(rt)
rownames(rt)=rt[,1]
exp=rt[,2:ncol(rt)]
dimnames=list(rownames(exp),colnames(exp))
mat=matrix(as.numeric(as.matrix(exp)),nrow=nrow(exp),dimnames=dimnames)
mat=avereps(mat)
mat=mat[rowMeans(mat)>0,]
rt <- t(mat)
rt <- cbind(id=rownames(rt),rt)

id <- substr(as.character(rt[,1]),1,16)
rt <- data.frame(id,rt)
rt <- rt[,-c(2)]
rt = as.matrix(rt)
rownames(rt)=rt[,1]
rt = rt[,-1]
mat = t(rt)


cluster=read.table("CRDscore.txt",sep="\t",header=T,check.names=F)
```

```
sameSample = intersect(cluster[,1], colnames(mat))
mat = mat[,sameSample]

exp=mat
dimnames=list(rownames(exp),colnames(exp))
mat=matrix(as.numeric(as.matrix(exp)),nrow=nrow(exp),dimnames=dimnames)
mat1 = log2(mat+1)

chemokines =
c("CCL3","CCL4","CCL5","CCL8","CCL20","CX3CL1","CXCL5","ICAM1","ICAM2","I
CAM3","ICAM5","VEGFA")
chemokines = as.data.frame(chemokines)

exp = mat1[chemokines[,1],]
cluster$Type = ifelse(cluster$CRDscore <= median(cluster$CRDscore),
"Low", "High")

rt = rbind(colnames(exp),exp)
rt = t(rt)

data = merge(cluster,rt,by.x=1,by.y=1)


A <- data[,-c(1:4)]
A=melt(A,id.vars=c("Type"))
colnames(A)=c("Type","Gene","Expression")
A[,3] = as.numeric(A[,3])

p=ggboxplot(A, x="Gene", y="Expression", color = "Type",
            ylab="log2 TPM",
            xlab="Immune Response-related Factors",
            #legend.title=x,
            palette = c("#E7B800","#00AFBB"),
            width=0.6, add = "none")
p=p+rotate_x_text(60)
p1=p+stat_compare_means(aes(group=Type),
                        method="anova",
                        symnum.args=list(cutpoints = c(0, 0.001, 0.01,
0.05, 1), symbols = c("***", "**", "*", "ns")),
                        label = "p.signif")


#D----
chemokines = c("CD40LG","ACE","CD28","LY9","TNFRSF14","CD84",
               "SLAMF1")
chemokines = as.data.frame(chemokines)

exp = mat1[chemokines[,1],]
cluster$Type = ifelse(cluster$CRDscore <= median(cluster$CRDscore),
"Low", "High")

rt = rbind(colnames(exp),exp)
rt = t(rt)

data = merge(cluster,rt,by.x=1,by.y=1)

A <- data[,-c(1:4)]
A=melt(A,id.vars=c("Type"))
colnames(A)=c("Type","Gene","Expression")
A[,3] = as.numeric(A[,3])
```

```
p=ggboxplot(A, x="Gene", y="Expression", color = "Type",
            ylab="log2 TPM",
            xlab="co-stimulatory",
            palette = c("#E7B800","#00AFBB"),
            width=0.6, add = "none")
p=p+rotate_x_text(60)
p1=p+stat_compare_means(aes(group=Type),
                        method="anova",
                        symnum.args=list(cutpoints = c(0, 0.001, 0.01, 
0.05, 1), symbols = c("***", "**", "*", "ns")),
                        label = "p.signif")



#E----
Antigen = c("HLA-DPB1","HLA-DQB2","HLA-DPA1","HLA-DRB5","HLA-DRB1","HLA-
DRA",
               "HLA-DQB1","HLA-DQA1","MICA","HLA-DQA2")
Antigen = as.data.frame(Antigen)
Antigen[,1] = gsub("\\.",replacement = "-",x=Antigen[,1] )
rownames(mat1) = gsub("\\.",replacement = "-",x=rownames(mat1))
same = intersect(Antigen[,1],rownames(mat1))

exp = mat1[Antigen[,1],]
cluster$Type = ifelse(cluster$CRDscore <= median(cluster$CRDscore), 
"Low", "High")

rt = rbind(colnames(exp),exp)
rt = t(rt)

data = merge(cluster,rt,by.x=1,by.y=1)


A <- data[,-c(1:4)]
A=melt(A,id.vars=c("Type"))
colnames(A)=c("Type","Gene","Expression")
A[,3] = as.numeric(A[,3])

p=ggboxplot(A, x="Gene", y="Expression", color = "Type",
            ylab="log2 TPM",
            xlab="Antigen Pressentation",
            #legend.title=x,
            palette = c("#E7B800","#00AFBB"),
            width=0.6, add = "none")
p=p+rotate_x_text(60)
p1=p+stat_compare_means(aes(group=Type),
                        method="anova",
                        symnum.args=list(cutpoints = c(0, 0.001, 0.01, 
0.05, 1), symbols = c("***", "**", "*", "ns")),
                        label = "p.signif")

#F----
inhibitory = c("LAG3","PDCD1","TNFRSF21",
               "CD247")
inhibitory = as.data.frame(inhibitory)

exp = mat1[inhibitory[,1],]
cluster$Type = ifelse(cluster$CRDscore <= median(cluster$CRDscore), 
"Low", "High")
```

```
rt = rbind(colnames(exp),exp)
rt = t(rt)

data = merge(cluster,rt,by.x=1,by.y=1)

A <- data[,-c(1:4)]
A=melt(A,id.vars=c("Type"))
colnames(A)=c("Type","Gene","Expression")
A[,3] = as.numeric(A[,3])

p=ggboxplot(A, x="Gene", y="Expression", color = "Type",
            ylab="log2 TPM",
            xlab="co-inhibitory",
            palette = c("#E7B800","#00AFBB"),
            width=0.6, add = "none")
p=p+rotate_x_text(60)
p1=p+stat_compare_means(aes(group=Type),
                        method="anova",
                        symnum.args=list(cutpoints = c(0, 0.001, 0.01,
0.05, 1), symbols = c("***", "**", "*", "ns")),
                        label = "p.signif")

#S3----
#A-C----
load("Tcell.Rdata")
select_genes <- c("GZMB","GNLY","NKG7",
                  "CTLA4","PDCD1","TIQIT","CXCL13",
                  "CCR7","LEF1","SELL","TCF7",
                  "IL12RB2","IFNG","STAT4",
                  "NR4A2","CREM","IRF4",
                  "STAT6","GATA3",
                  "IKZF2","FOXP3","IL2RA"
)

DotPlot(pbmc1, features = select_genes )+
  scale_y_discrete(limits =
c("12","24","15","18","22","0","1","16","5","6","7","10",

"11","13","14","17","20","27","4","19","2","8","9","21",
                              "25","26","3","23"))+
  coord_flip()


A = pbmc1@meta.data
A$seurat_clusters = as.numeric(A$seurat_clusters)
gene=read.table("T_cell.txt", header=T, sep="\t", check.names=F)
j=1
for (i in 1:28) {
  A$seurat_clusters[which(A$seurat_clusters == i)] <- gene[j,2]
  j=j+1
}

pbmc1@meta.data$singleR = A$seurat_clusters

DimPlot(pbmc1, reduction = "umap",label = F,group.by = "singleR")


#D-F----
load("scRNA.Rdata")
```

```
meat = scRNA@meta.data
meat = meat[meat$Type == "Macrophage",]
pbmc1 = scRNA[,rownames(meat)]
exp = as.data.frame(pbmc1@assays[["RNA"]]@counts)

pbmc1 <- CreateSeuratObject(counts = exp)


pbmc1 <- NormalizeData(pbmc1, normalization.method = "LogNormalize",
scale.factor = 10000)


pbmc1 <- FindVariableFeatures(pbmc1, selection.method = "vst", nfeatures
= 2000)

#Scaling the data
all.genes <- rownames(pbmc1)
pbmc1 <- ScaleData(pbmc1, features = all.genes)

#PCA
pbmc1 <- RunPCA(pbmc1, features = VariableFeatures(object = pbmc1))

A = pbmc1@meta.data
A$id = substr(rownames(A),18,25)
table(A$id)
pbmc1@meta.data$id=A$id

scRNA <- RunHarmony(pbmc1, group.by.vars="id", max.iter.harmony =
20,assay.use = "RNA")

pc.num=1:30
scRNA <- RunTSNE(scRNA, dims=pc.num) %>% RunUMAP(dims=pc.num)
scRNA <- FindNeighbors(scRNA, dims = pc.num)
scRNA <- FindClusters(scRNA, resolution = 0.5)


DimPlot(scRNA, reduction = "tsne",label = T)



select_genes <-
c("IL1A","IL1B","IL6","IL12A","IL12B","IL23A","TNF","FCGR3A","FCGR2A","FC
GR1A","CD68","CD80","CD86","CLEC7A","MERTK","IRF5","STAT1","HLA-
DPA1","HLA-DPB1","HLA-DQA1","HLA-DQA2","HLA-DQB1","HLA-DQB2","HLA-DQB1-
AS1","HLA-DRA","HLA-DRB1","HLA-DRB5",

"IDO1","IL10","TGFB1","CSF1R","MSR1","CD163","MRC1","CD209","FCER1A","VSI
G4","IRF4","STAT6")


DotPlot(scRNA, features = select_genes )+ coord_flip()+
  scale_y_discrete(limits = c(
    "15","10","4","1","17","12","5","3","11","8","9","18",
    "0","2","6","7","13","16"
  ))

score = read.table("score.txt", header=T, sep="\t", check.names=F)
score = score[colnames(scRNA),]
scRNA$Type = score$Type
```

```
celltype = read.table("Mac.txt", header=T, sep="\t", check.names=F)
clusters <- scRNA@meta.data$seurat_clusters
scRNA@meta.data$singleR=celltype[match(clusters,celltype$ClusterID),'cell type']
DimPlot(scRNA, group.by="singleR",  label.size=5, reduction='tsne')
DimPlot(scRNA, group.by="Type",  label.size=5, reduction='tsne')


save(scRNA,file='Macrophage.Rdata')



#S4----
#DE----
ann = read.table("cli.txt",sep="\t",header=T,check.names=F,row.names = 1)
CNV = read.table("CNV_score.txt",sep="\t",header=T,check.names=F)
ann = ann[14]
data = merge(CNV,ann,by.x=2,by.y= 0)
table(data$`Treatement Timepoint`)

rt = data[,c(2,5)]
colnames(rt)=c("Expression","cancer")

group=levels(factor(rt$cancer))
rt$cancer=factor(rt$cancer, levels=group)
comp=combn(group,2)
my_comparisons=list()
for(i in 1:ncol(comp)){my_comparisons[[i]]<-comp[,i]}


ggplot(data=rt,aes(x =reorder(cancer,Expression,  FUN = median), y=Expression, color=cancer))+
  stat_boxplot(geom="errorbar",width=0.5,size=1.3)+
  geom_boxplot(alpha=1,outlier.shape = NA,size=1.3,width=0.5,fatten=1)+
  theme_bw()+
  theme(panel.grid=element_blank())+
  stat_compare_means(comparisons = my_comparisons,symnum.args=list(cutpoints = c(0, 0.001, 0.01, 0.05, 1), symbols = c("***", "**", "*", "ns")),label = "p.signif")+
  theme(legend.position = "right")+
  ggtitle("") +
  xlab("")+
  ylab("CNV Score")+
  rotate_x_text(60)+
  theme(
    axis.title.x=element_blank(),
    axis.text.y = element_text(size = 15,face = "bold",color = "black"),
    axis.title.y = element_text(size = 15,face = "bold",color = "black"),
    axis.text.x = element_text(size = 15,face = "bold",color = "black"),
    legend.position = "none",
    plot.title = element_text(face = "bold",size=15,hjust = 0.5))


rt = data[,c(2,4)]
colnames(rt)=c("Expression","cancer")


group=levels(factor(rt$cancer))
rt$cancer=factor(rt$cancer, levels=group)
comp=combn(group,2)
```

```
my_comparisons=list()
for(i in 1:ncol(comp)){my_comparisons[[i]]<-comp[,i]}

p1=ggplot(data=rt,aes(x =reorder(cancer,Expression,  FUN = median),
y=Expression, color=cancer))+
  stat_boxplot(geom="errorbar",width=0.5,size=1.3)+
  geom_boxplot(alpha=1,outlier.shape = NA,size=1.3,width=0.5,fatten=1)+
  theme_bw()+
  theme(panel.grid=element_blank())+
  stat_compare_means(comparisons =
my_comparisons,symnum.args=list(cutpoints = c(0, 0.001, 0.01, 0.05, 1),
symbols = c("***", "**", "*", "ns")),label = "p.signif")+
  theme(legend.position = "right")+
  ggtitle("") +
  xlab("")+
  ylab("CNV Score")+
  rotate_x_text(60)+
  scale_color_manual(values=c("#00AFBB", "#E7B800"))+
  theme(
    axis.title.x=element_blank(),
    axis.text.y = element_text(size = 15,face = "bold",color = "black"),
    axis.title.y = element_text(size = 15,face = "bold",color = "black"),
    axis.text.x = element_text(size = 15,face = "bold",color = "black"),
    legend.position = "none",
    plot.title = element_text(face = "bold",size=15,hjust = 0.5))




#F----
load("scRNA.Rdata")
ann = read.table("cli.txt",sep="\t",header=T,check.names=F,row.names = 1)
A = scRNA@meta.data
ann = ann[rownames(A),]
scRNA@meta.data$cell = ann$singleR1
scRNA@meta.data$Type = ann$`Treatement Timepoint`
A = scRNA@meta.data
X = A[A$singleR1 != "Hepatocytes",]

B = A[A$Type == "PD",]
C = A[A$Type == "TN",]
D = A[A$Type == "RD",]

scRNA = scRNA[,rownames(X)]

scRNA_B = scRNA[,rownames(B)]
scRNA_C = scRNA[,rownames(C)]
scRNA_D = scRNA[,rownames(D)]

p1=DimPlot(scRNA_B, reduction = "umap",group.by = "cell")
p3=DimPlot(scRNA_C, reduction = "umap",group.by = "cell")
p2=DimPlot(scRNA_D, reduction = "umap",group.by = "cell")
p = p1+p2+p3
p



#G----
ann = read.table("cli.txt",sep="\t",header=T,check.names=F,row.names = 1)
```

```
rt = ann[,c(17,7)]
colnames(rt)=c("Expression","cancer")
rt[,"cancer"]=ifelse(rt[,"cancer"] <= 60 , "<=60", ">60")


group=levels(factor(rt$cancer))
rt$cancer=factor(rt$cancer, levels=group)
comp=combn(group,2)
my_comparisons=list()
for(i in 1:ncol(comp)){my_comparisons[[i]]<-comp[,i]}

p1=ggplot(data=rt,aes(x =reorder(cancer,Expression,  FUN = median),
y=Expression, color=cancer))+
  stat_boxplot(geom="errorbar",width=0.5,size=1.3)+
  geom_boxplot(alpha=1,outlier.shape = NA,size=1.3,width=0.5,fatten=1)+
  theme_bw()+
  theme(panel.grid=element_blank())+
  stat_compare_means(comparisons =
my_comparisons,symnum.args=list(cutpoints = c(0, 0.001, 0.01, 0.05, 1),
symbols = c("***", "**", "*", "ns")),label = "p.signif")+
  theme(legend.position = "right")+
  ggtitle("") +
  xlab("")+
  ylab("RloopScore")+
  rotate_x_text(60)+
  theme(
    axis.title.x=element_blank(),
    axis.text.y = element_text(size = 15,face = "bold",color = "black"),
    axis.title.y = element_text(size = 15,face = "bold",color = "black"),
    axis.text.x = element_text(size = 15,face = "bold",color = "black"),
    legend.position = "none",
    plot.title = element_text(face = "bold",size=15,hjust = 0.5))


#S5----
#Rloop

rt=read.table("TCGA.txt",sep="\t",header=T,check.names=F,row.names = 1)
rt$Type = ifelse(rt$CRDscore <= median(rt$CRDscore), "Low", "High")
rt$Type <- factor(rt$Type,levels=c("Low","High"))
rt$Type = ifelse(rt$CRDscore <= median(rt$CRDscore), "1", "0")

cox <- coxph(Surv(futime, fustat) ~ rt[,4], data = rt)
coxSummary = summary(cox)
coxP=coxSummary$coefficients[,"Pr(>|z|)"]
outTab=data.frame()
outTab=rbind(outTab,
             cbind(id="TCGA",
                   HR=coxSummary$conf.int[,"exp(coef)"],
                   HR.95L=coxSummary$conf.int[,"lower .95"],
                   HR.95H=coxSummary$conf.int[,"upper .95"],
                   pvalue=coxSummary$coefficients[,"Pr(>|z|)"])
)

samples=list.files("./")
out = file.path('./',samples)


A = c()
for (i in out) {
```

```
  rt=read.table(i,sep="\t",header=T,check.names=F,row.names = 1)
  rt$Type = ifelse(rt$CRDscore <= median(rt$CRDscore), "Low", "High")
  outTab=data.frame()
  B = sub(".//",replacement = "",i)
  C = sub(".txt",replacement = "",B)

  cox <- coxph(Surv(futime, fustat) ~ rt[,4], data = rt)
  coxSummary = summary(cox)
  coxP=coxSummary$coefficients[,"Pr(>|z|)"]
  outTab=rbind(outTab,
               cbind(id=C,
                     HR=coxSummary$conf.int[,"exp(coef)"],
                     HR.95L=coxSummary$conf.int[,"lower .95"],
                     HR.95H=coxSummary$conf.int[,"upper .95"],
                     pvalue=coxSummary$coefficients[,"Pr(>|z|)"])
  )

  A = rbind(A,outTab)
}

write.table(A, file="rloop.txt", sep="\t", quote=F, row.names=F)



#Age

Age=read.table("AGe.txt",sep="\t",header=T,check.names=F,row.names = 1)
os=read.table("Total.txt",sep="\t",header=T,check.names=F,row.names = 1)
Age = merge(os,Age,by=0)
rownames(Age) = Age[,1]
Age = Age[,-c(1,2)]

samples=list.files("./1/")
out = file.path('./1/',samples)

A = c()
for (i in out) {
  rt=read.table(i,sep="\t",header=T,check.names=F,row.names = 1)
  same = intersect(rownames(rt),rownames(Age))
  if(length(same) == 0){ next}
  rt=Age[rownames(rt),]
  outTab=data.frame()
  B = sub(".//",replacement = "",i)
  B = sub("./",replacement = "",B)
  C = sub(".txt",replacement = "",B)

  cox <- coxph(Surv(futime, fustat) ~ rt[,3], data = rt)
  coxSummary = summary(cox)
  coxP=coxSummary$coefficients[,"Pr(>|z|)"]
  outTab=rbind(outTab,
               cbind(id=C,
                     HR=coxSummary$conf.int[,"exp(coef)"],
                     HR.95L=coxSummary$conf.int[,"lower .95"],
                     HR.95H=coxSummary$conf.int[,"upper .95"],
                     pvalue=coxSummary$coefficients[,"Pr(>|z|)"])
  )

  A = rbind(A,outTab)
}
```

```
write.table(A, file="Age_HR.txt", sep="\t", quote=F, row.names=F)

#EGFR

Age=read.table("EGFR.txt",sep="\t",header=T,check.names=F,row.names = 1)
os=read.table("Total.txt",sep="\t",header=T,check.names=F,row.names = 1)
Age = merge(os,Age,by=0)
rownames(Age) = Age[,1]
Age = Age[,-c(1,2)]

samples=list.files("./1/")
out = file.path('./1/',samples)

A = c()
for (i in out) {
  rt=read.table(i,sep="\t",header=T,check.names=F,row.names = 1)
  same = intersect(rownames(rt),rownames(Age))
  if(length(same) == 0){ next}
  rt=Age[rownames(rt),]
  outTab=data.frame()
  B = sub(".//",replacement = "",i)
  B = sub("./",replacement = "",B)
  C = sub(".txt",replacement = "",B)

  cox <- coxph(Surv(futime, fustat) ~ rt[,3], data = rt)
  coxSummary = summary(cox)
  coxP=coxSummary$coefficients[,"Pr(>|z|)"]
  outTab=rbind(outTab,
               cbind(id=C,
                     HR=coxSummary$conf.int[,"exp(coef)"],
                     HR.95L=coxSummary$conf.int[,"lower .95"],
                     HR.95H=coxSummary$conf.int[,"upper .95"],
                     pvalue=coxSummary$coefficients[,"Pr(>|z|)"])
  )

  A = rbind(A,outTab)
}

write.table(A, file="EGFR_HR.txt", sep="\t", quote=F, row.names=F)

#KRAS
Age=read.table("KRAS.txt",sep="\t",header=T,check.names=F,row.names = 1)
os=read.table("Total.txt",sep="\t",header=T,check.names=F,row.names = 1)
Age = merge(os,Age,by=0)
rownames(Age) = Age[,1]
Age = Age[,-c(1,2)]

samples=list.files("./1/")
out = file.path('./1/',samples)

A = c()
for (i in out) {
  rt=read.table(i,sep="\t",header=T,check.names=F,row.names = 1)
  same = intersect(rownames(rt),rownames(Age))
  if(length(same) == 0){ next}
  rt=Age[rownames(rt),]
  outTab=data.frame()
  B = sub(".//",replacement = "",i)
  B = sub("./",replacement = "",B)
  C = sub(".txt",replacement = "",B)
```

```
  cox <- coxph(Surv(futime, fustat) ~ rt[,3], data = rt)
  coxSummary = summary(cox)
  coxP=coxSummary$coefficients[,"Pr(>|z|)"]
  outTab=rbind(outTab,
               cbind(id=C,
                     HR=coxSummary$conf.int[,"exp(coef)"],
                     HR.95L=coxSummary$conf.int[,"lower .95"],
                     HR.95H=coxSummary$conf.int[,"upper .95"],
                     pvalue=coxSummary$coefficients[,"Pr(>|z|)"])
  )

  A = rbind(A,outTab)
}

write.table(A, file="KRAS_HR.txt", sep="\t", quote=F, row.names=F)

#sex

Age=read.table("Gender.txt",sep="\t",header=T,check.names=F,row.names =
1)
os=read.table("Total.txt",sep="\t",header=T,check.names=F,row.names = 1)
Age = merge(os,Age,by=0)
rownames(Age) = Age[,1]
Age = Age[,-c(1,2)]

samples=list.files("./1/")
out = file.path('./1/',samples)

A = c()
for (i in out) {
  rt=read.table(i,sep="\t",header=T,check.names=F,row.names = 1)
  same = intersect(rownames(rt),rownames(Age))
  if(length(same) == 0){ next}
  rt=Age[rownames(rt),]
  outTab=data.frame()
  B = sub(".//",replacement = "",i)
  B = sub("./",replacement = "",B)
  C = sub(".txt",replacement = "",B)

  cox <- coxph(Surv(futime, fustat) ~ rt[,3], data = rt)
  coxSummary = summary(cox)
  coxP=coxSummary$coefficients[,"Pr(>|z|)"]
  outTab=rbind(outTab,
               cbind(id=C,
                     HR=coxSummary$conf.int[,"exp(coef)"],
                     HR.95L=coxSummary$conf.int[,"lower .95"],
                     HR.95H=coxSummary$conf.int[,"upper .95"],
                     pvalue=coxSummary$coefficients[,"Pr(>|z|)"])
  )

  A = rbind(A,outTab)
}

write.table(A, file="sex_HR.txt", sep="\t", quote=F, row.names=F)

#smoker

Age=read.table("Smoker.txt",sep="\t",header=T,check.names=F,row.names =
1)
```

```
os=read.table("Total.txt",sep="\t",header=T,check.names=F,row.names = 1)
Age = merge(os,Age,by=0)
rownames(Age) = Age[,1]
Age = Age[,-c(1,2)]

samples=list.files("./1/")
out = file.path('./1/',samples)

A = c()
for (i in out) {
  rt=read.table(i,sep="\t",header=T,check.names=F,row.names = 1)
  same = intersect(rownames(rt),rownames(Age))
  if(length(same) == 0){ next}
  rt=Age[rownames(rt),]
  outTab=data.frame()
  B = sub(".//",replacement = "",i)
  B = sub("./",replacement = "",B)
  C = sub(".txt",replacement = "",B)

  cox <- coxph(Surv(futime, fustat) ~ rt[,3], data = rt)
  coxSummary = summary(cox)
  coxP=coxSummary$coefficients[,"Pr(>|z|)"]
  outTab=rbind(outTab,
               cbind(id=C,
                     HR=coxSummary$conf.int[,"exp(coef)"],
                     HR.95L=coxSummary$conf.int[,"lower .95"],
                     HR.95H=coxSummary$conf.int[,"upper .95"],
                     pvalue=coxSummary$coefficients[,"Pr(>|z|)"])
  )

  A = rbind(A,outTab)
}

write.table(A, file="smoker_HR.txt", sep="\t", quote=F, row.names=F)

#stage
Age=read.table("Stage.txt",sep="\t",header=T,check.names=F,row.names = 1)
os=read.table("Total.txt",sep="\t",header=T,check.names=F,row.names = 1)
Age = merge(os,Age,by=0)
rownames(Age) = Age[,1]
Age = Age[,-c(1,2)]

samples=list.files("./1/")
out = file.path('./1/',samples)

A = c()
for (i in out) {
  rt=read.table(i,sep="\t",header=T,check.names=F,row.names = 1)
  same = intersect(rownames(rt),rownames(Age))

  if(length(same) == 0){ next}
  rt=Age[rownames(rt),]
  outTab=data.frame()
  B = sub(".//",replacement = "",i)
  B = sub("./",replacement = "",B)
  C = sub(".txt",replacement = "",B)
  if( C == "GSE31210"){ next}
  if( C == "GSE50081"){ next}

  cox <- coxph(Surv(futime, fustat) ~ rt[,3], data = rt)
```

```
  coxSummary = summary(cox)
  coxP=coxSummary$coefficients[,"Pr(>|z|)"]
  outTab=rbind(outTab,
               cbind(id=C,
                     HR=coxSummary$conf.int[,"exp(coef)"],
                     HR.95L=coxSummary$conf.int[,"lower .95"],
                     HR.95H=coxSummary$conf.int[,"upper .95"],
                     pvalue=coxSummary$coefficients[,"Pr(>|z|)"])
  )

  A = rbind(A,outTab)
}

write.table(A, file="stage_HR.txt", sep="\t", quote=F, row.names=F)

#TP53
Age=read.table("TP53.txt",sep="\t",header=T,check.names=F,row.names = 1)
os=read.table("Total.txt",sep="\t",header=T,check.names=F,row.names = 1)
Age = merge(os,Age,by=0)
rownames(Age) = Age[,1]
Age = Age[,-c(1,2)]

samples=list.files("./1/")
out = file.path('./1/',samples)

A = c()
for (i in out) {
  rt=read.table(i,sep="\t",header=T,check.names=F,row.names = 1)
  same = intersect(rownames(rt),rownames(Age))
  if(length(same) == 0){ next}
  rt=Age[rownames(rt),]
  outTab=data.frame()
  B = sub(".//",replacement = "",i)
  B = sub("./",replacement = "",B)
  C = sub(".txt",replacement = "",B)

  cox <- coxph(Surv(futime, fustat) ~ rt[,3], data = rt)
  coxSummary = summary(cox)
  coxP=coxSummary$coefficients[,"Pr(>|z|)"]
  outTab=rbind(outTab,
               cbind(id=C,
                     HR=coxSummary$conf.int[,"exp(coef)"],
                     HR.95L=coxSummary$conf.int[,"lower .95"],
                     HR.95H=coxSummary$conf.int[,"upper .95"],
                     pvalue=coxSummary$coefficients[,"Pr(>|z|)"])
  )

  A = rbind(A,outTab)
}

write.table(A, file="TP53_HR.txt", sep="\t", quote=F, row.names=F)

#all
inputFile="all.txt"

rt=read.table(inputFile,header=T,sep="\t",row.names=1,check.names=F)
gene=rownames(rt)
hr=sprintf("%.3f",rt$"HR")
hrLow=sprintf("%.3f",rt$"HR.95L")
hrHigh=sprintf("%.3f",rt$"HR.95H")
```

```
Hazard.ratio=paste0(hr,"(",hrLow,"-",hrHigh,")")
pVal=ifelse(rt$pvalue<0.001, "<0.001", sprintf("%.3f", rt$pvalue))

pdf(file=outFile, width = 7, height =10)
n=nrow(rt)
nRow=n+1
ylim=c(1,nRow)
layout(matrix(c(1,2),nc=2),width=c(3,2))


xlim = c(0,3)
par(mar=c(4,2,1.5,1.5))
plot(1,xlim=xlim,ylim=ylim,type="n",axes=F,xlab="",ylab="")
text.cex=0.8
text(0,n:1,gene,adj=0,cex=text.cex)
text(1.5-0.5*0.2,n:1,pVal,adj=1,cex=text.cex);text(1.5-
0.5*0.2,n+1,'pvalue',cex=text.cex,font=2,adj=1)
text(3,n:1,Hazard.ratio,adj=1,cex=text.cex);text(3,n+1,'Hazard
ratio',cex=text.cex,font=2,adj=1,)


par(mar=c(4,1,1.5,1),mgp=c(2,0.5,0))
xlim = c(0,max(as.numeric(hrLow),as.numeric(hrHigh)))
plot(1,xlim=xlim,ylim=ylim,type="n",axes=F,ylab="",xaxs="i",xlab="Hazard
ratio")
arrows(as.numeric(hrLow),n:1,as.numeric(hrHigh),n:1,angle=90,code=3,lengt
h=0.03,col="darkblue",lwd=2.5)
abline(v=1,col="black",lty=2,lwd=2)
boxcolor = ifelse(as.numeric(hr) > 1, 'red', 'blue')
points(as.numeric(hr), n:1, pch = 15, col = boxcolor, cex=1.3)
axis(1)




#S6----
dat=read.table("LUAD.TPM.txt",header=T,sep="\t",row.names=1,check.names=F
)
os=read.table("os.txt",header=T,sep="\t",row.names=1,check.names=F)
colnames(dat) = substr(as.character(colnames(dat)),1,16)
A = as.data.frame(t(dat["FANCI",]))
rownames(A) = gsub(".",replacement = "-",rownames(A),fixed = T)

rt1 = merge(A,os,by=0)

var="FANCI"
rt1$futime = rt1$futime/365

res.cut=surv_cutpoint(rt1, time="futime", event="fustat",
variables=c("FANCI"))
cutoff=as.numeric(res.cut$cutpoint[1])
print(cutoff)
group=ifelse(rt1[,"FANCI"]<=cutoff, "Low", "High")


diff=survdiff(Surv(futime, fustat) ~group,data = rt1)
pValue=1-pchisq(diff$chisq,df=1)
if(pValue<0.001){
  pValue="p<0.001"
}else{
```

```
  pValue=paste0("p=",sprintf("%.03f",pValue))
}
fit <- survfit(Surv(futime, fustat) ~ group, data = rt1)

p=ggsurvplot(
  fit, #
  pval = TRUE, #
  pval.method = TRUE,
  pval.size=5,#
  legend.title='FANCI',
  legend.labs=c('High','Low'),
  conf.int = TRUE,
  risk.table = TRUE,
  linetype = "strata",
  xlab="Time(years)",
  break.time.by = 2,
  surv.median.line = "hv",
  ggtheme = theme_bw(),
  palette = c("#E31A1C","#1F78B4")
)
p
```
