# Supplementary material for: Aberrant R-loop–mediated immune evasion, cellular communication, and metabolic reprogramming affect cancer progression: a single-cell analysis
Source: Mol Cancer. 2024 Jan 10;23:11. doi: 10.1186/s12943-023-01924-6 (PMC10777569; doi:10.1186/s12943-023-01924-6)
Supplement: Supplementary file 2 — Additional file 2: Figure S1. Systematic literature search in the databases PubMed. Numbers show the results acquired for each step. [file 12943_2023_1924_MOESM2_ESM.pdf]

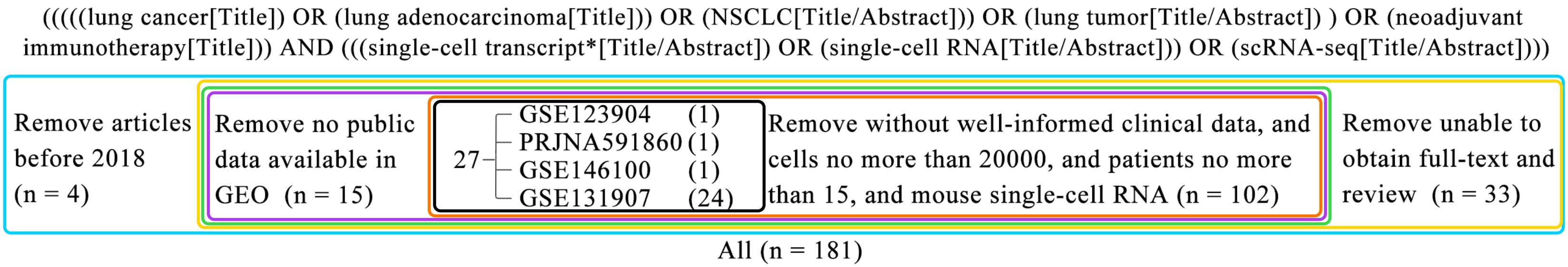
(((((lung cancer[Title]) OR (lung adenocarcinoma[Title])) OR (NSCLC[Title/Abstract])) OR (lung tumor[Title/Abstract])) OR (neoadjuvant immunotherapy[Title])) AND (((single-cell transcript*[Title/Abstract]) OR (single-cell RNA[Title/Abstract])) OR (scRNA-seq[Title/Abstract])))
Remove articles before 2018 (n = 4)
Remove no public data available in GEO (n = 15)
27
GSE123904 (1)
PRJNA591860 (1)
GSE146100 (1)
GSE131907 (24)
Remove without well-informed clinical data, and cells no more than 20000, and patients no more than 15, and mouse single-cell RNA (n = 102)
Remove unable to obtain full-text and review (n = 33)
All (n = 181)
