# Supplementary figures and images for: Aberrant R-loop–mediated immune evasion, cellular communication, and metabolic reprogramming affect cancer progression: a single-cell analysis
Source: Mol Cancer. 2024 Jan 10;23:11. doi: 10.1186/s12943-023-01924-6 (PMC10777569; doi:10.1186/s12943-023-01924-6)

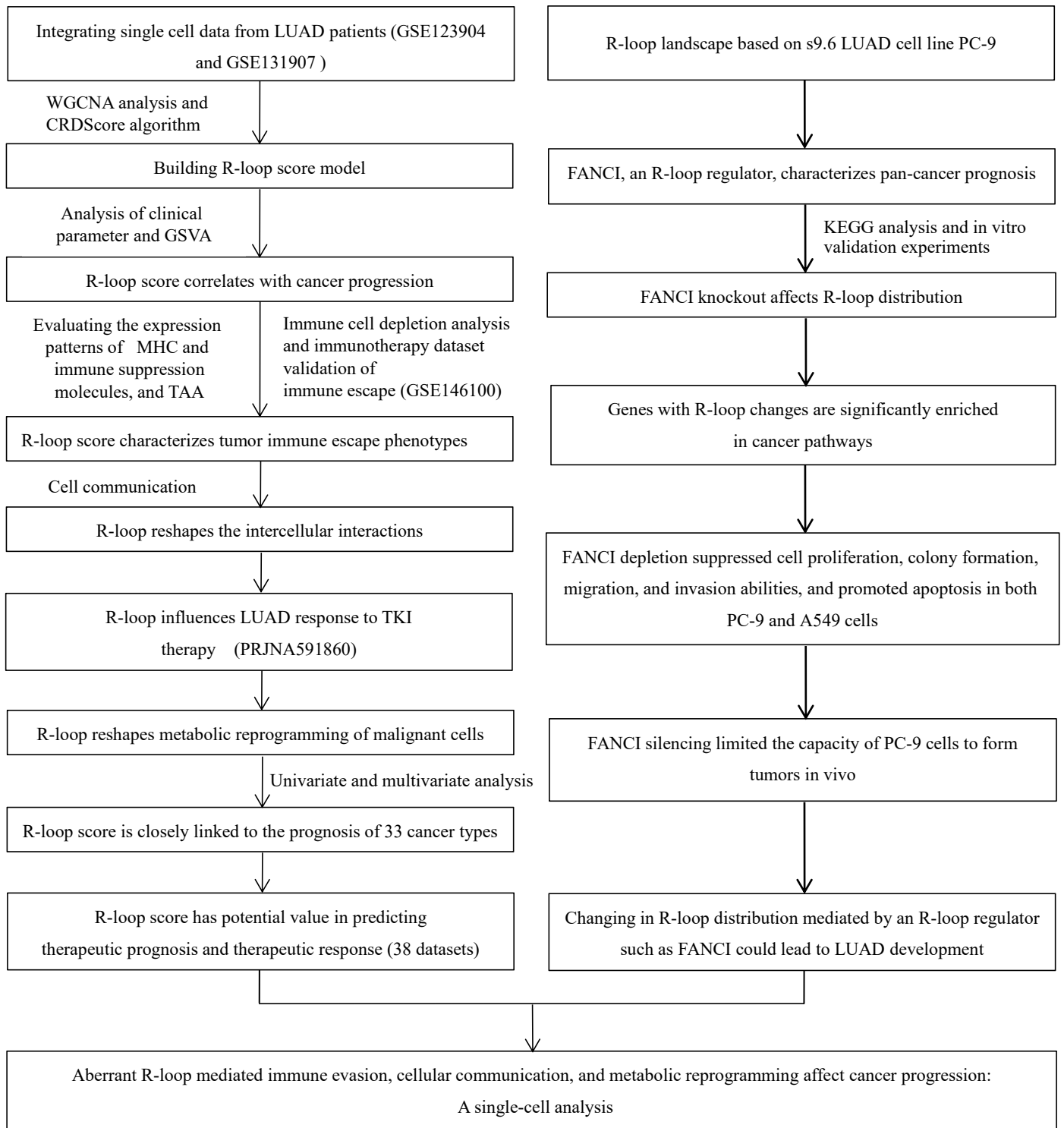

Supplement: Supplementary file 3 — Additional file 3: Figure S2. Overview of study design. [file 12943_2023_1924_MOESM3_ESM.pdf]

**A**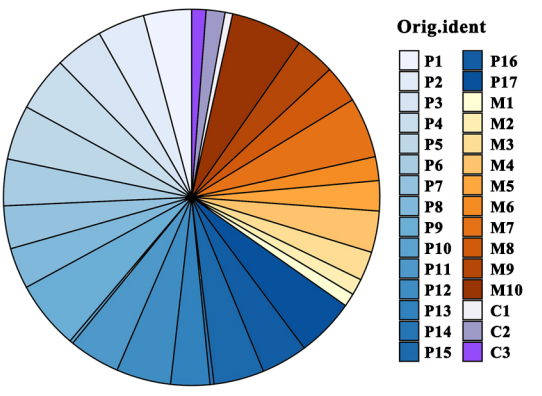**B**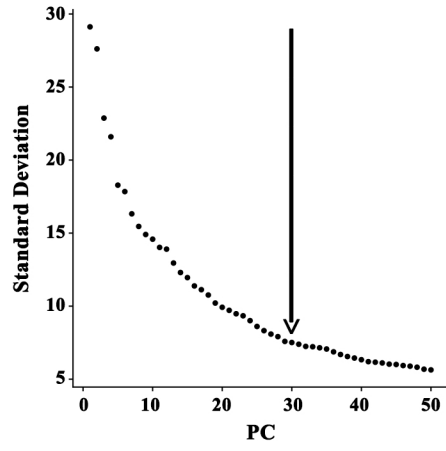**C**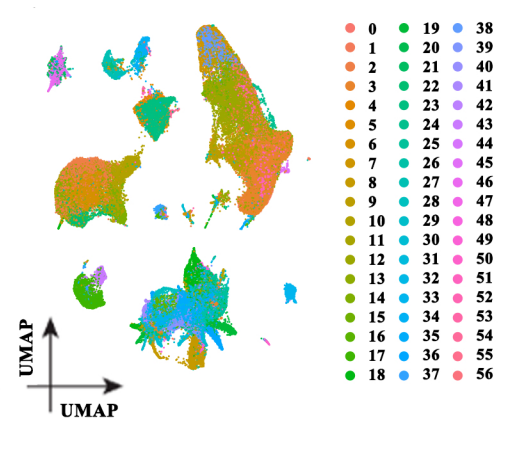**D**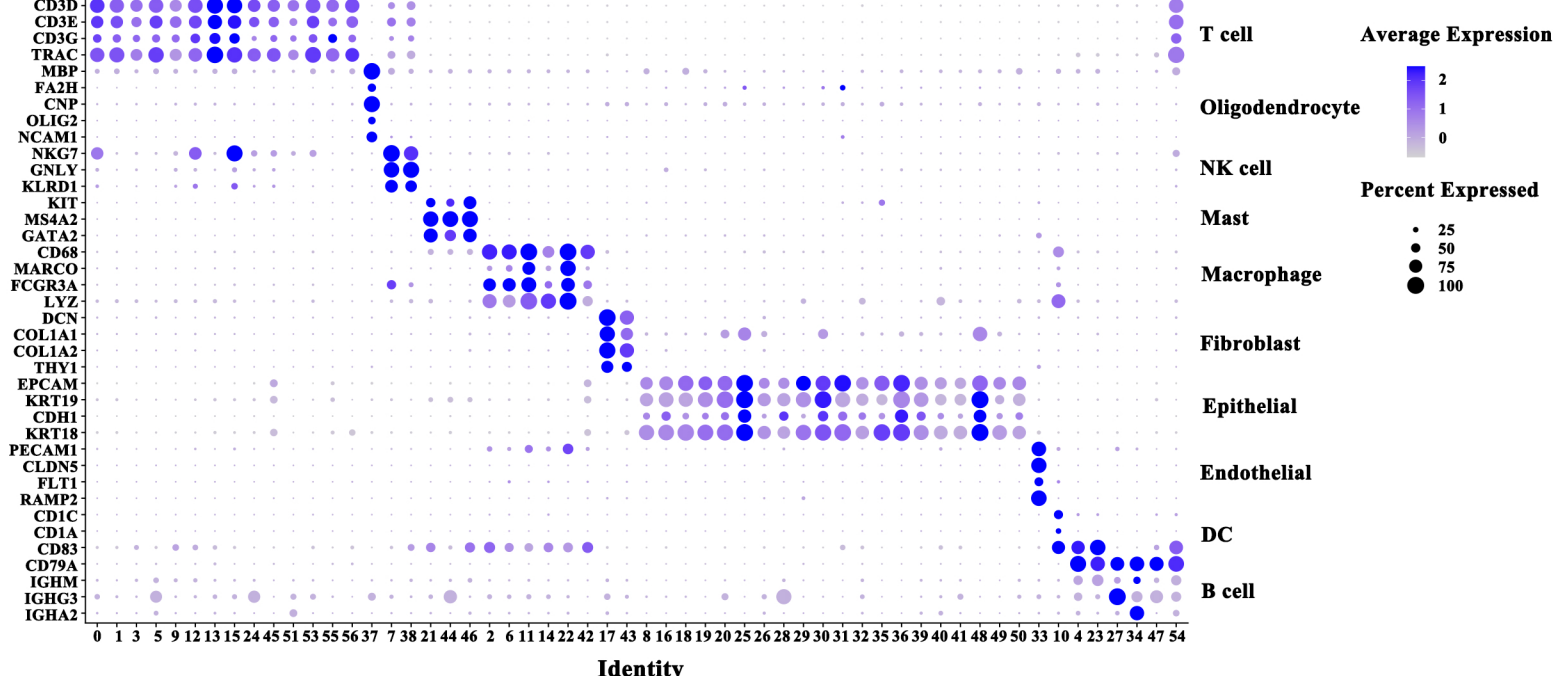**E**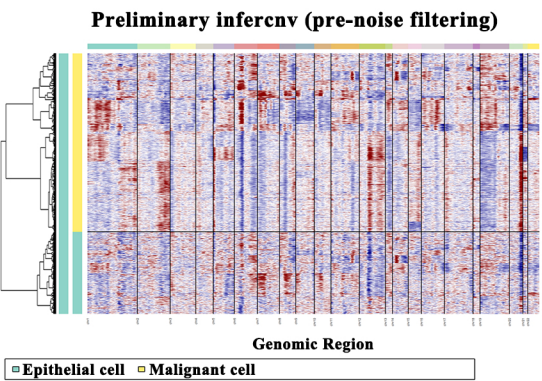**F**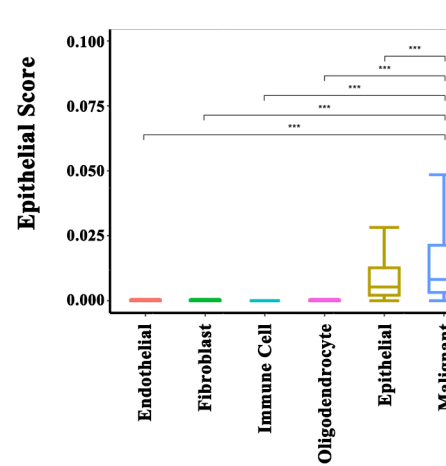**G**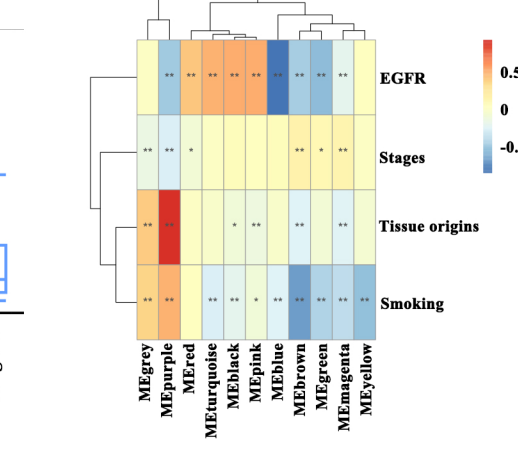**H**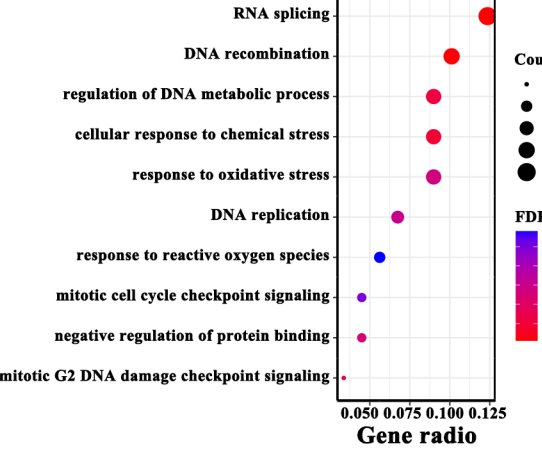**I**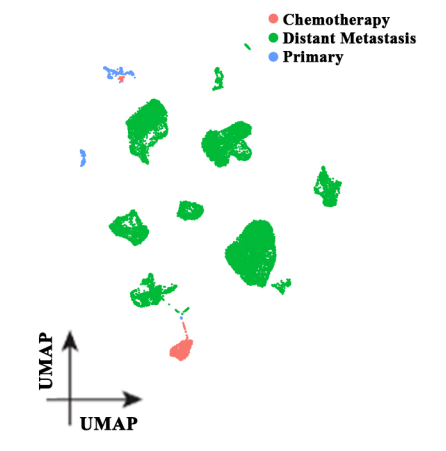**J**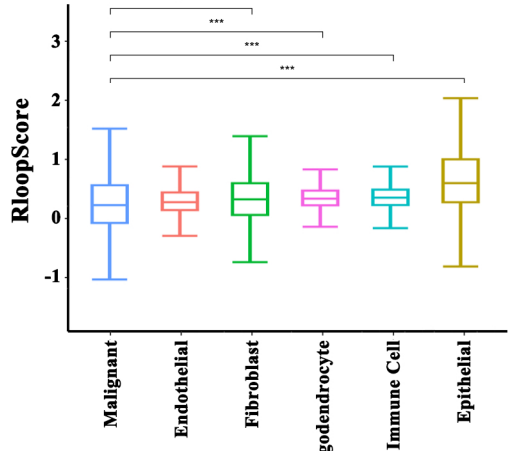**K**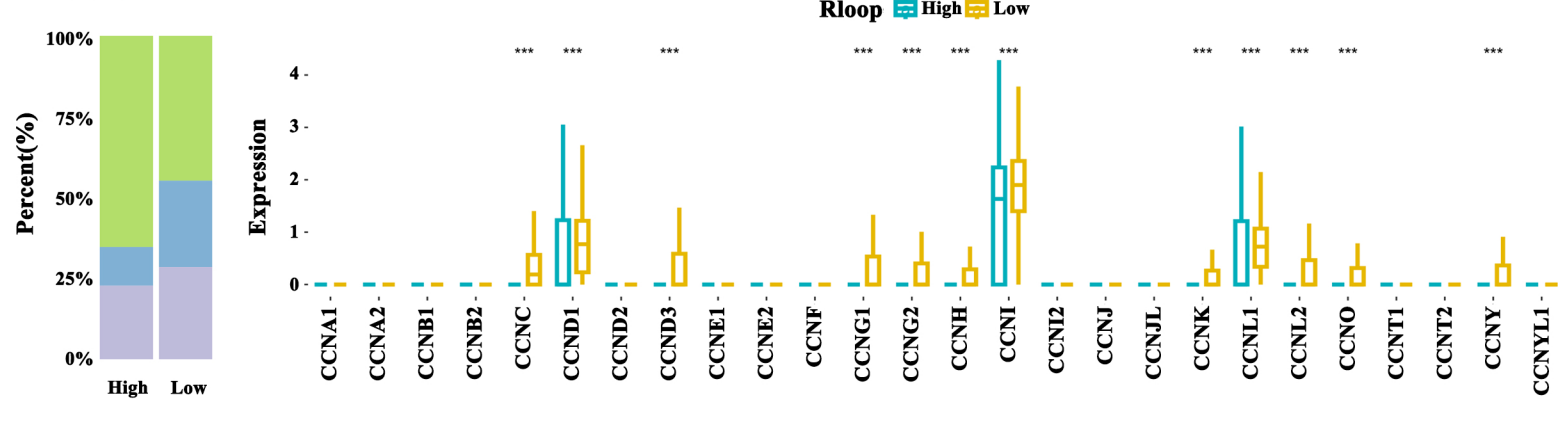

Supplement: Supplementary file 4 — Additional file 4: Figure S3. Determining cell types for integrated cohorts (GSE123904 and GSE131907). (A) The proportion of cells in each patient. (B) The number of principal components for clustering. (C) Uniform manifold approximation and projection (UMAP) plot of 92,842 cells colored according to cell subgroup. (D) Average and percentage of the expression of marker genes in 57 subgroups. (E) Relative expression intensity in each chromosome identified by inferCNV. Amplifications and deletions on the indicated chromosomes shown in red and blue, respectively. (F) Epithelial scores for each cell type such as malignant cells, epithelial cells, melanocytes, immune cells, fibroblasts, and endothelial cells according to the expression of epithelial marker genes (Wilcoxon rank test). (G) Association between module eigengenes and clinical characteristics examined using weighted gene coexpression network analysis (WGCNA; Pearson’s correlation test). (H) Gene Ontology (GO) biological process (BP) terms enriched in 92 R-loop regulators. (I) UMAP plot of malignant cells colored according to sample origins. (J) R-loop score in each cell type (Wilcoxon rank test). (K) Proportions of malignant cells in G1, G2M, and S stages from high- and low-score groups, and the difference in the expression of cyclin genes between these two groups (Wilcoxon rank test). (*p < .05, **p < .01, ***p < .001). [file 12943_2023_1924_MOESM4_ESM.pdf]

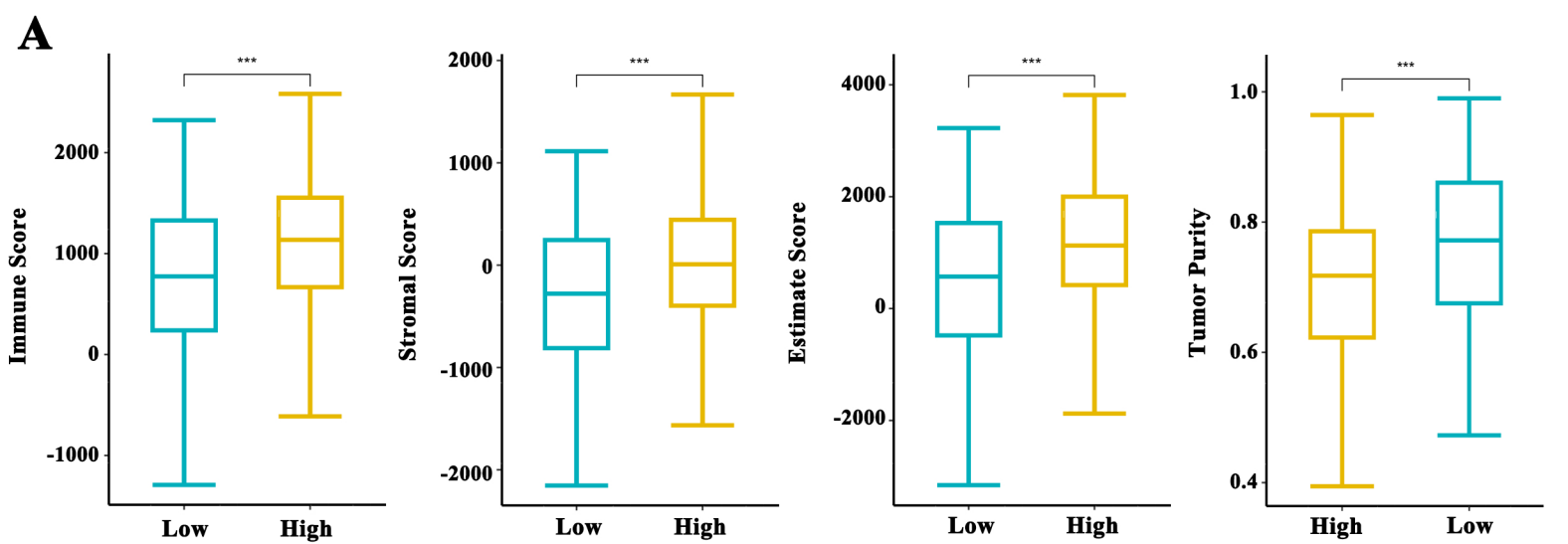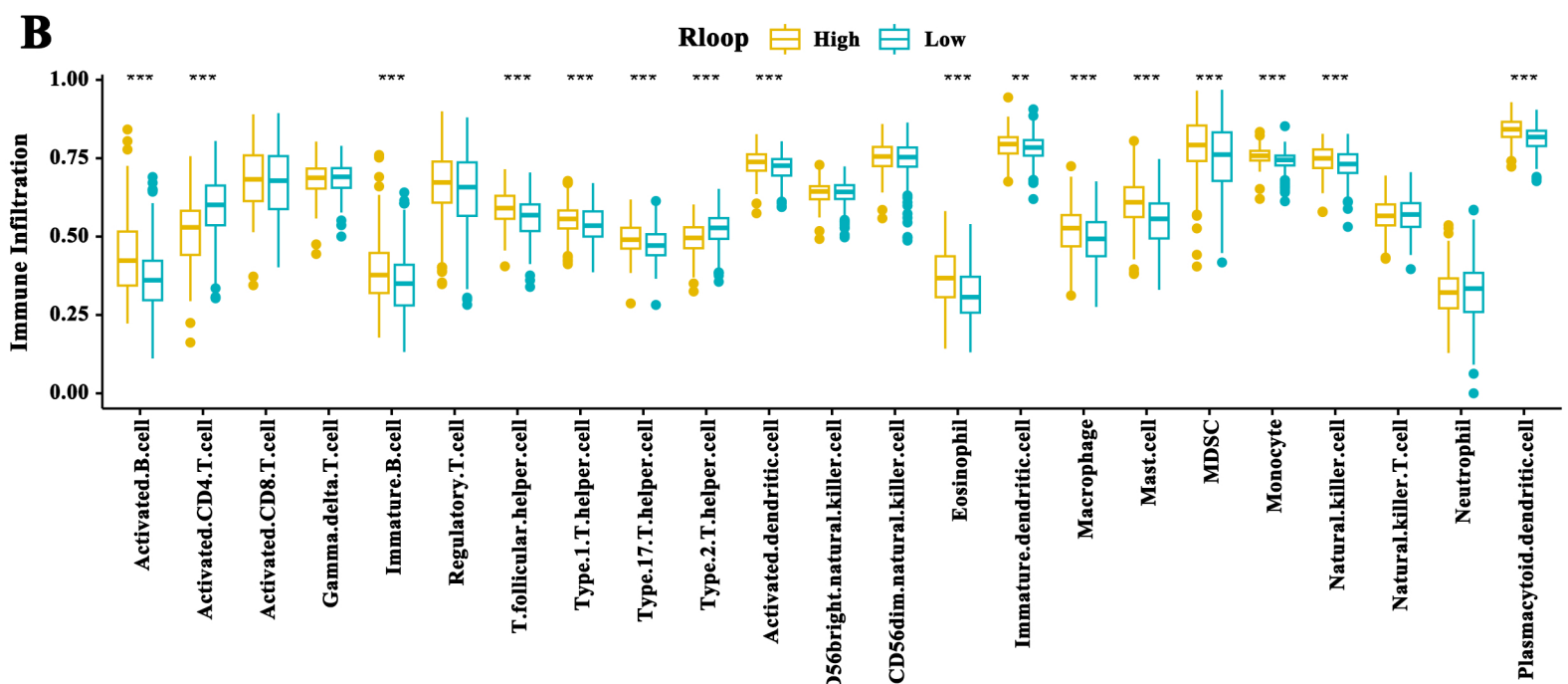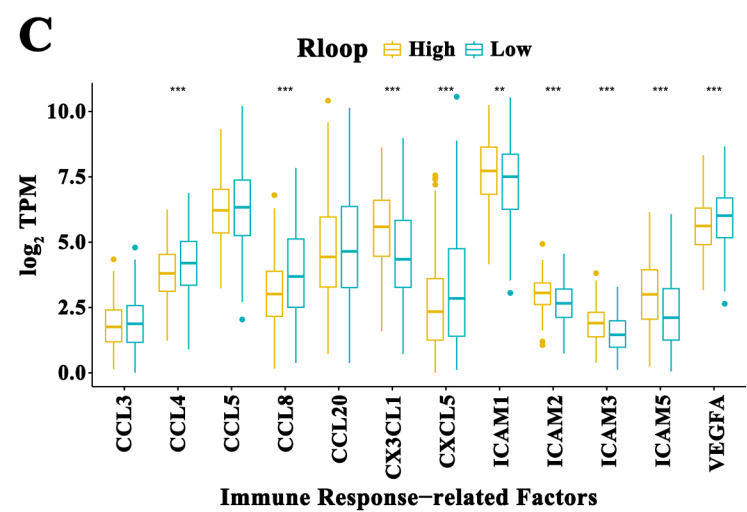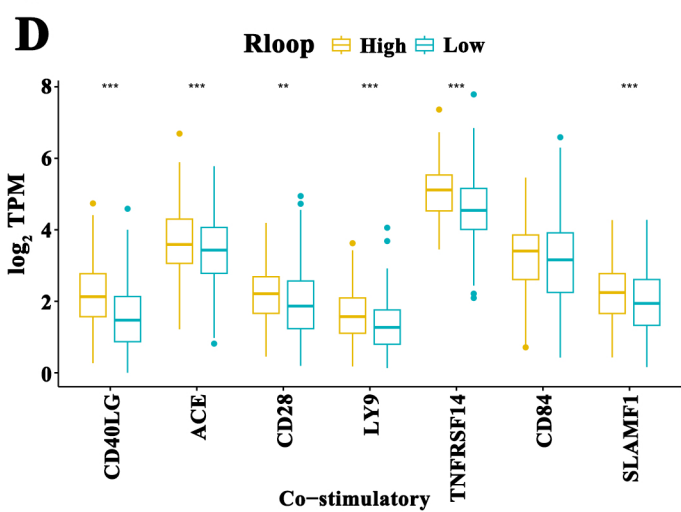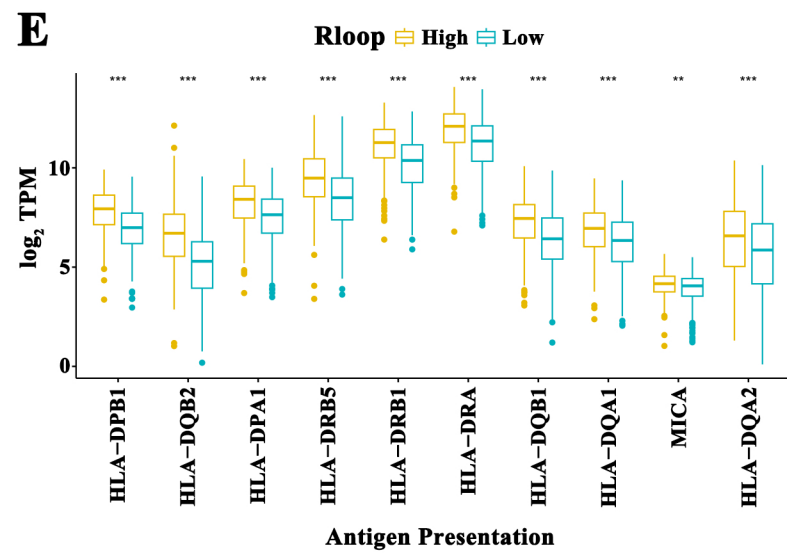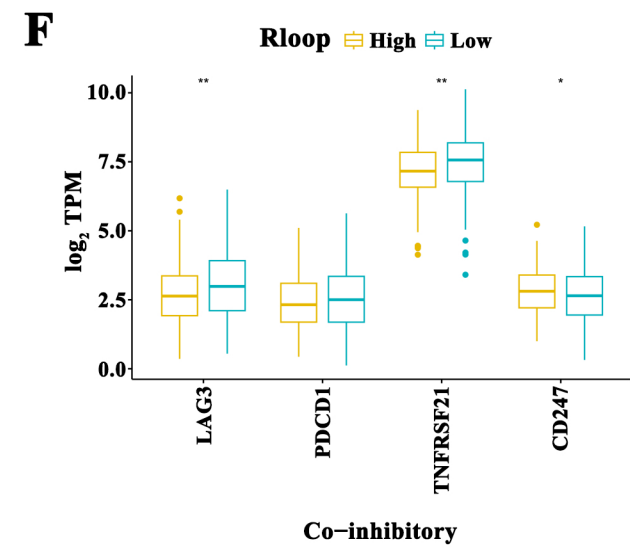

Supplement: Supplementary file 5 — Additional file 5: Figure S4. Differences in the immune microenvironment of patients from TCGA-LUAD in the R-loop score subgroups. (A) Patients with LUAD were divided into high- and low-score subgroups according to the median value of R-loop scores. Comparison of immune score, stromal score, ESTIMATE score, and tumor purity between the high- and low-score subgroups. (B) The difference in the enrichment scores of immune cells between the two subgroups. (C-F) The expression profiles of immune response–related factors (C), costimulatory molecules (D), antigen presentation molecules (E), and coinhibitory molecules (F) in the high- and low-score subgroups. (Wilcoxon rank test; *p < .05, **p < .01, ***p < .001). [file 12943_2023_1924_MOESM5_ESM.pdf]

**A**

UMAP

UMAP

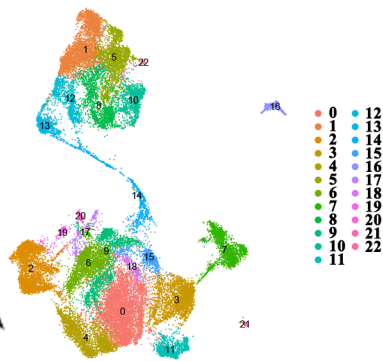**B**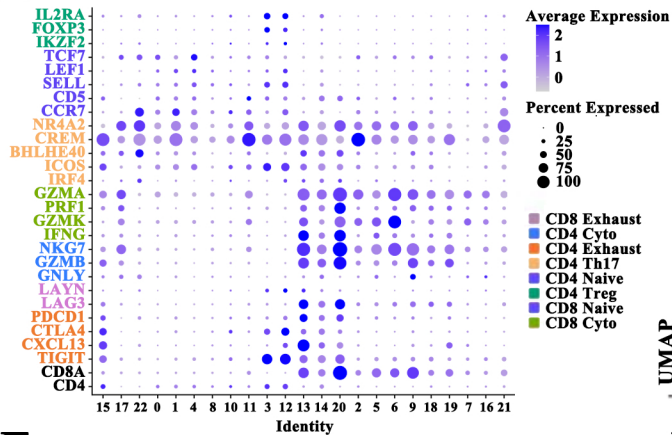**C**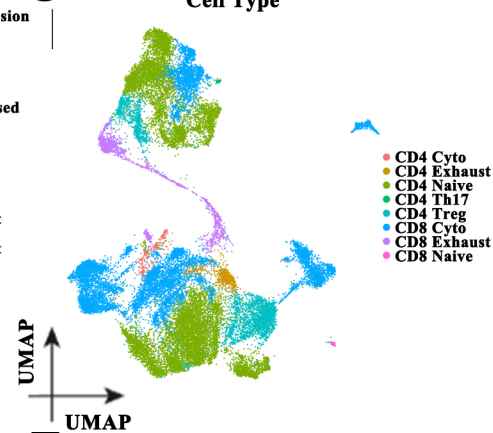**D**

tSNE

tSNE

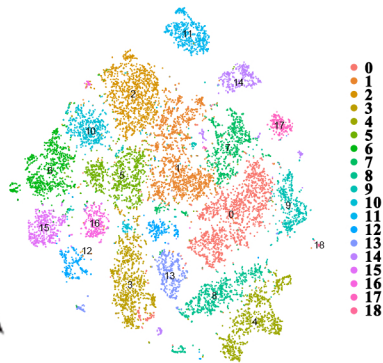**E**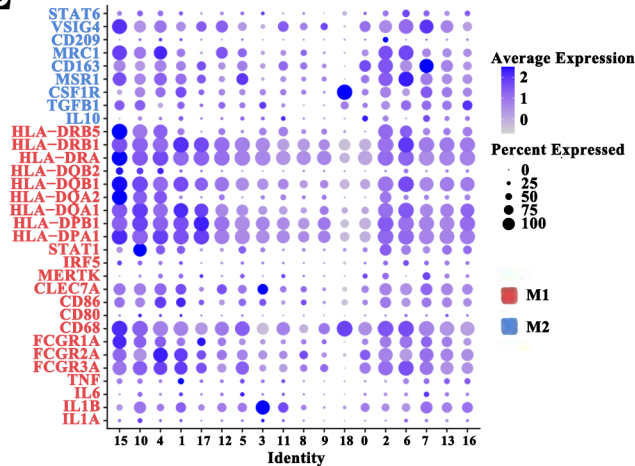**F**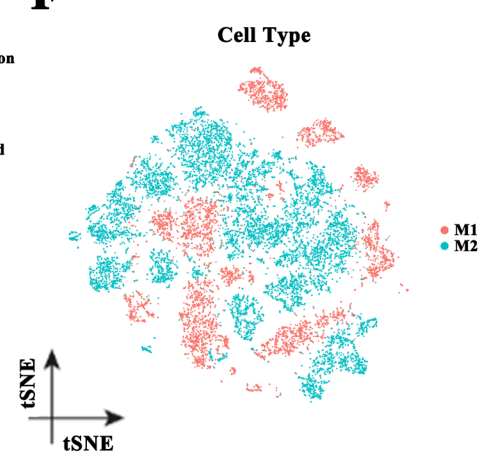

Supplement: Supplementary file 6 — Additional file 6: Figure S5. Subpopulations of T cells and macrophage cells from GSE123904 and GSE131907. (A) Uniform manifold approximation and projection (UMAP) plot of 31,201 T cells colored according to cell subclusters. (B) Average and percentage of the expression of marker genes in the T cell subclusters. (C) UMAP plot of T cells colored according to T cell type. (D) T-distributed stochastic neighbor embedding (t-SNE) plot of 14,737 macrophage cells colored according to cell subclusters. (E) Average and percentage of the expression of marker genes in the macrophage subclusters. (F) Subpopulations of the identified macrophage cells. [file 12943_2023_1924_MOESM6_ESM.pdf]

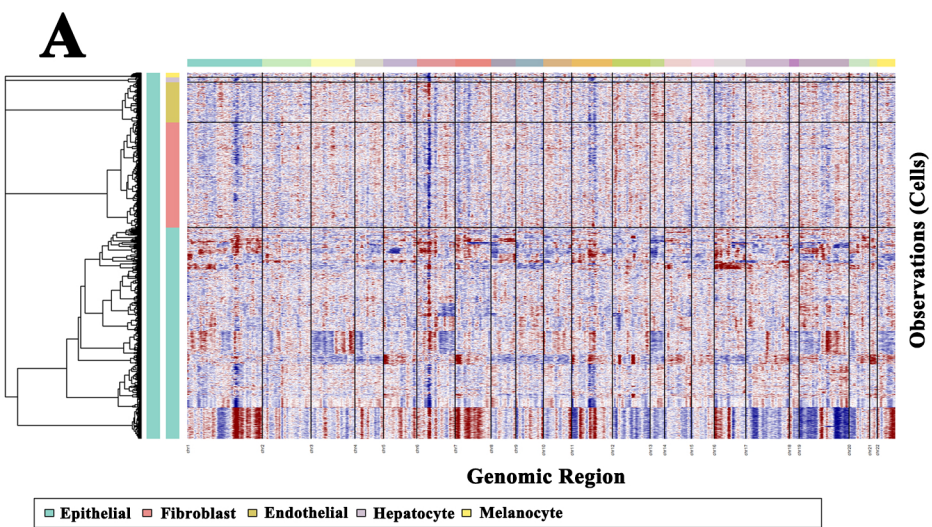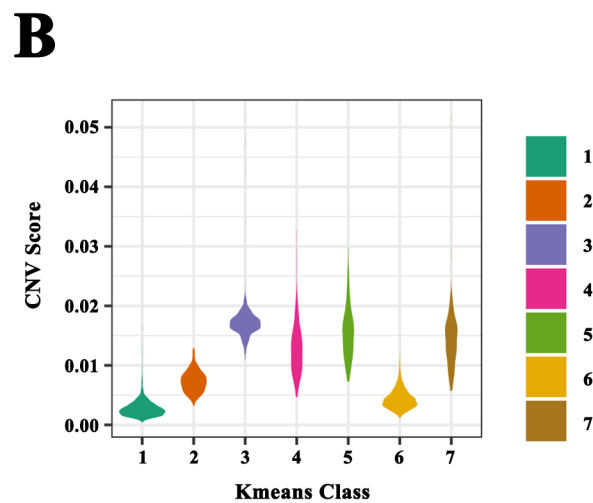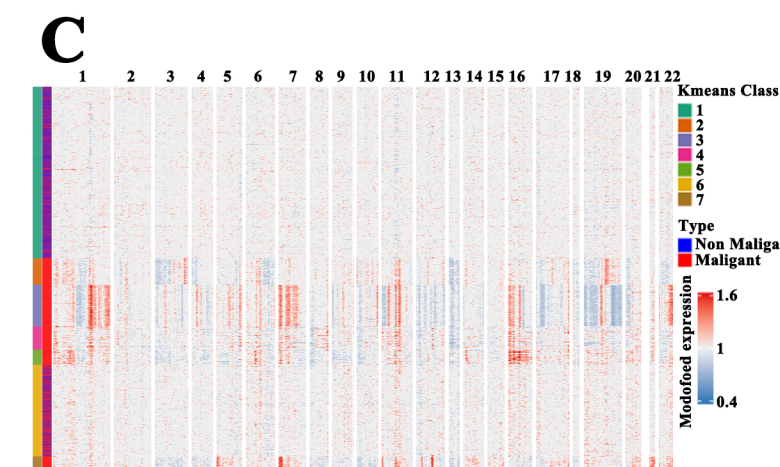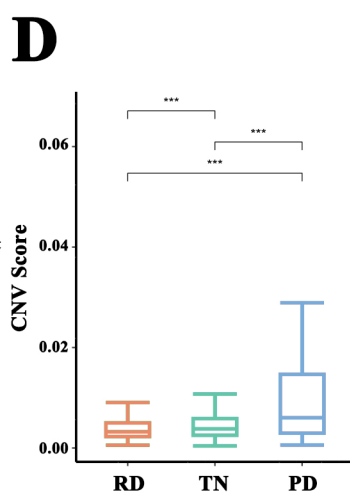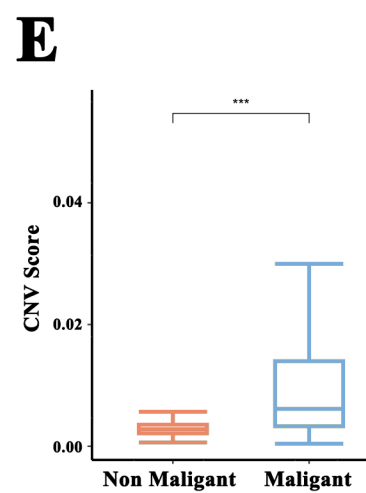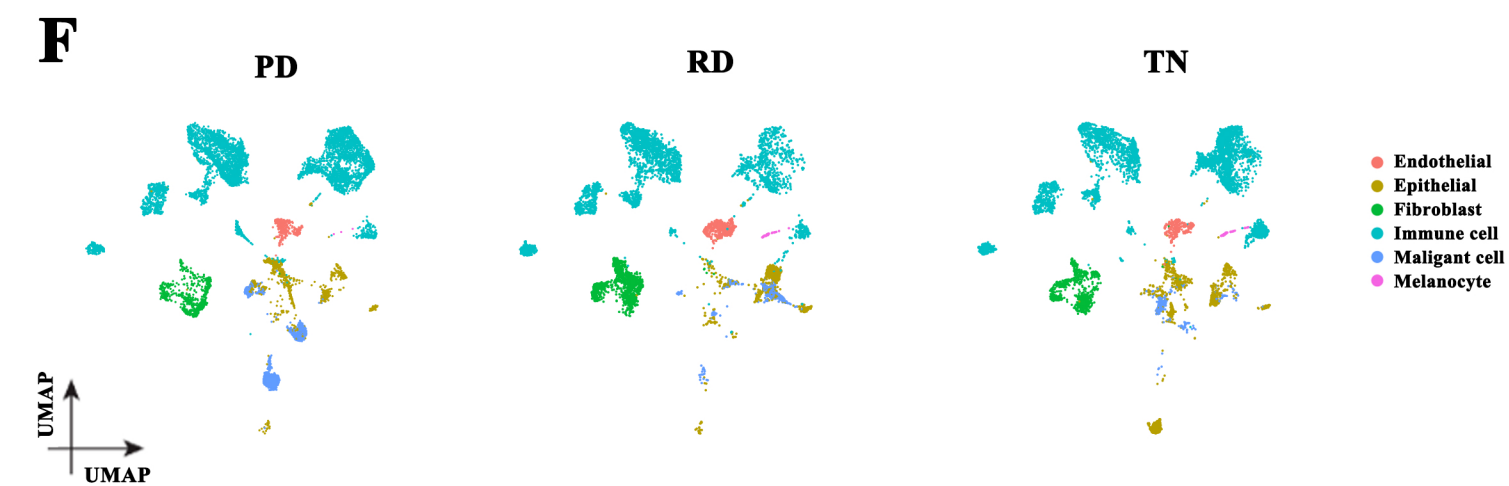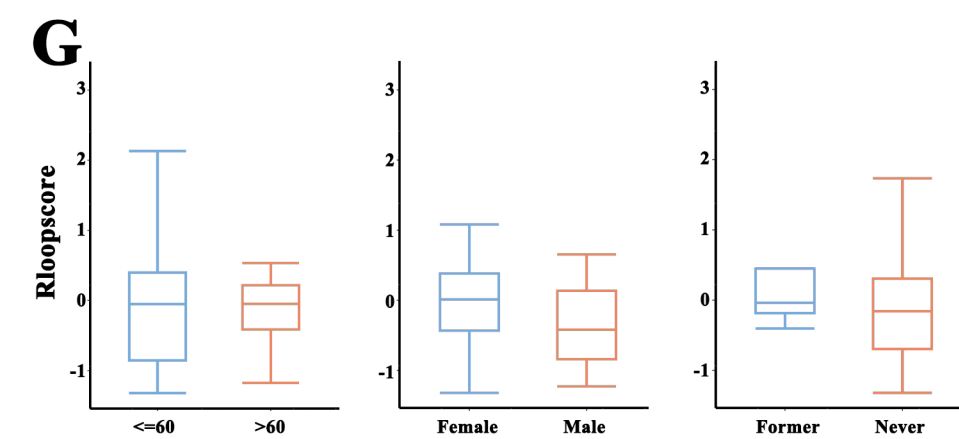

Supplement: Supplementary file 7 — Additional file 7: Figure S6. Identification of malignant cells from patients who underwent tyrosine kinase inhibitor (TKI) therapy (PRJNA591860). (A) Relative expression intensity in each chromosome identified by inferCNV (all cell types). Amplifications and deletions on the indicated chromosomes shown in red and blue, respectively. (B) Copy number variation (CNV) scores for each epithelial cell subgroup. (C) Relative expression intensity in each chromosome (epithelial cells). (D) The difference in the CNV scores among patients classified into the initiating targeted therapy (TN), complete or partial response state (RD), and progressive disease (PD) subgroups. (E) CNV levels calculated by the quadratic sum of CNV regions for malignant and nonmalignant subgroups. (F) Uniform manifold approximation and projection (UMAP) plot of the PD, RD, and TN subgroups colored according to cell type. (G) R-loop scores of age, sex, and smoking status subgroups according to the mean value of all malignant cells. (Wilcoxon rank test; *p < .05, **p < .01, ***p < .001). [file 12943_2023_1924_MOESM7_ESM.pdf]

**A**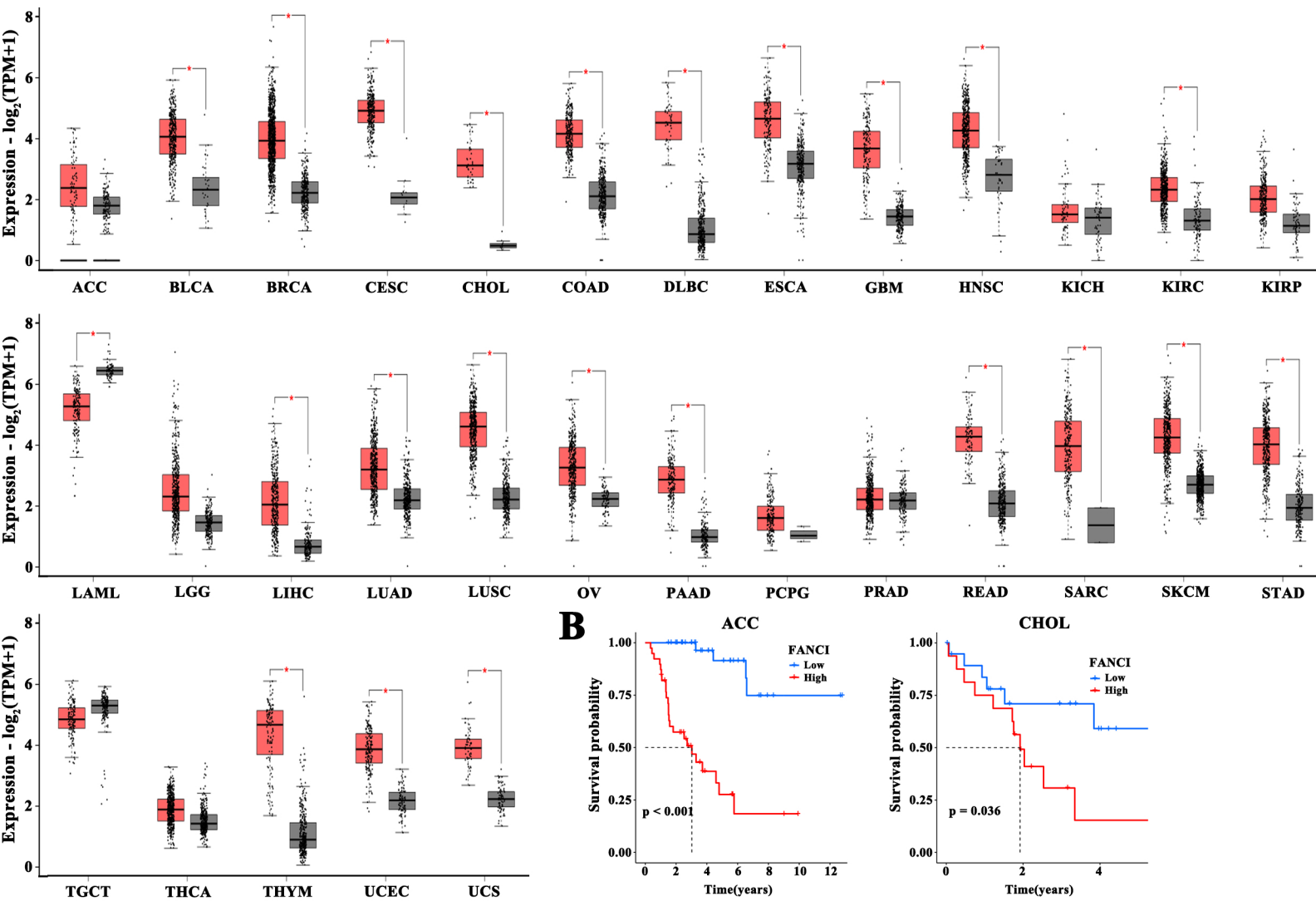**B**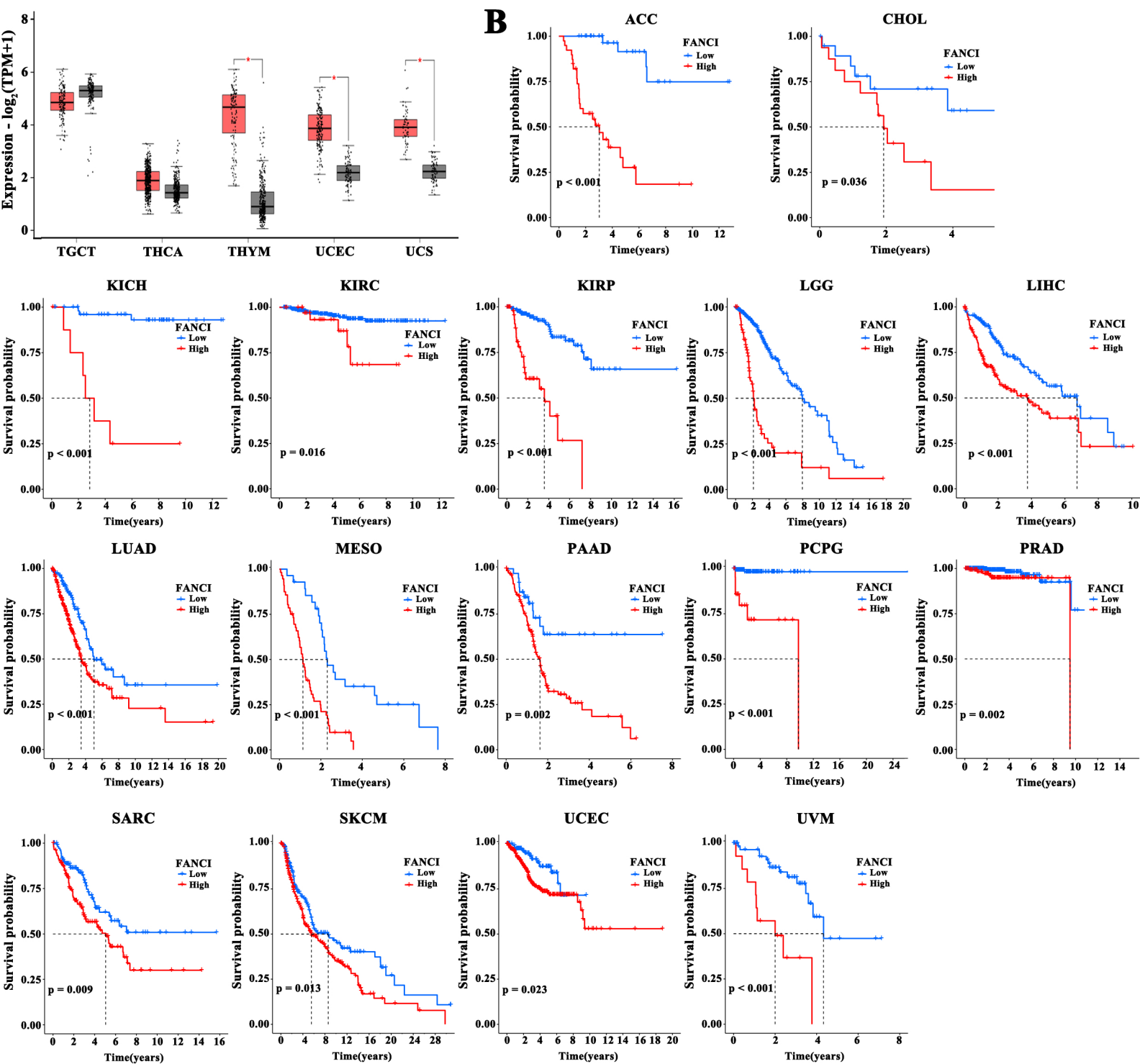

Supplement: Supplementary file 9 — Additional file 9: Figure S8. FANCI was highly expressed in all cancer types, and its high expression was associated with poor outcomes. (A) The difference in FANCI expression between tumor and normal control tissues in 31 cancer types (Wilcoxon rank test). (B) The difference in the overall survival probability between patients with high and low FANCI expression (log-rank test). (*p < .05, **p < .01, ***p < .001). [file 12943_2023_1924_MOESM9_ESM.pdf]

**p-ERK**

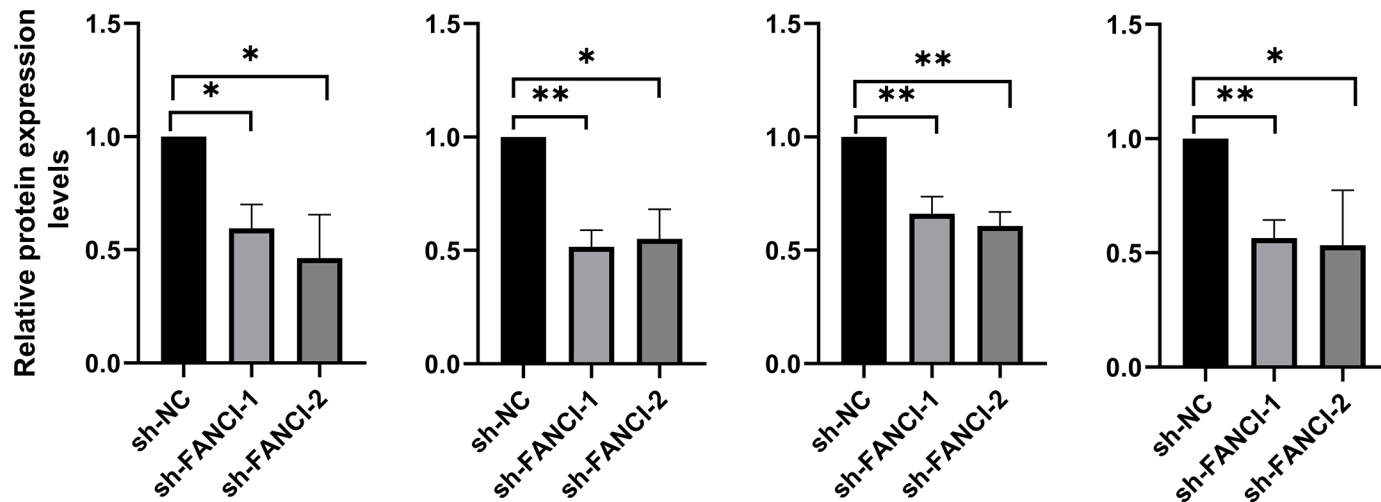

**NFκB**

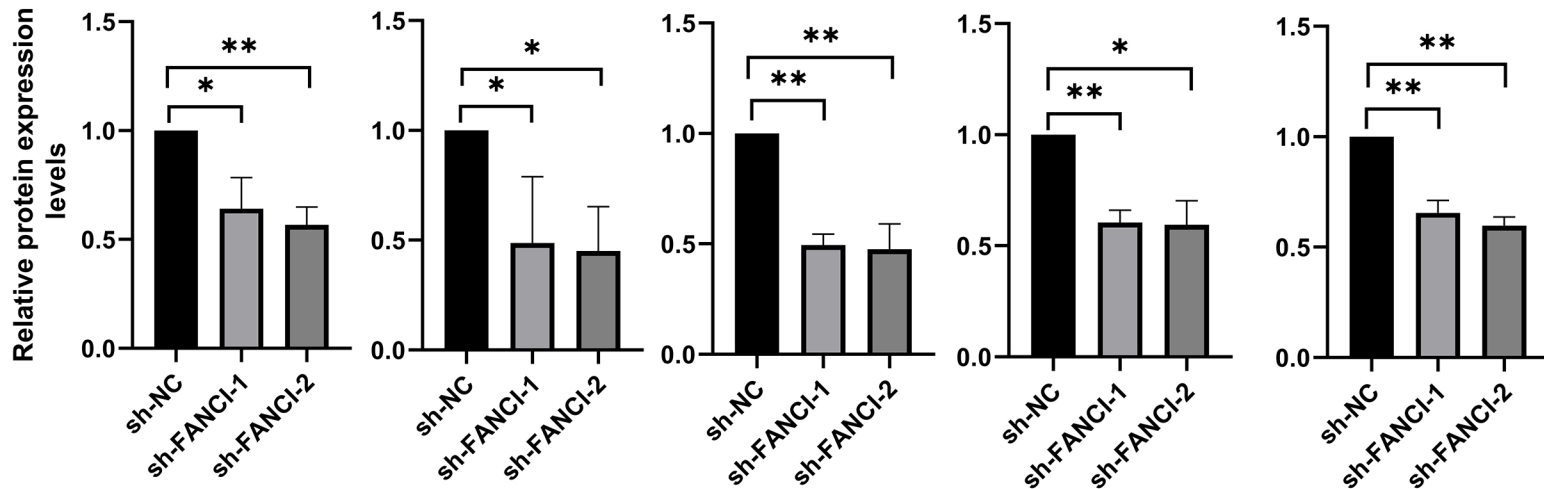

Supplement: Supplementary file 10 — Additional file 10: Figure S9. FANCI silencing decreased the protein levels of RasGRF1, p-Raf1, p-MEK, p-ERK, PI3K, p-PI3K, p-AKT, p-IKK, and NFκB in PC-9 cells. (Student’s t-test; *p < 0.05; **p < 0.01; ***p < 0.001). [file 12943_2023_1924_MOESM10_ESM.pdf]
