# Supplementary material for: Aberrant R-loop–mediated immune evasion, cellular communication, and metabolic reprogramming affect cancer progression: a single-cell analysis
Source: Mol Cancer. 2024 Jan 10;23:11. doi: 10.1186/s12943-023-01924-6 (PMC10777569; doi:10.1186/s12943-023-01924-6)
Supplement: Supplementary file 8 — Additional file 8: Figure S7. Clinical characteristics of multiple factors in the independent cohorts. (A) Nonparametric estimates of the dependence of all-time risk of mortality on R-loop score using smoothHR, and the difference in the overall survival rate between patients with high and low R-loop scores. (B) Hazard ratios (HRs; 95% confidence intervals) based on Cox proportional hazard models (log-rank test). [file 12943_2023_1924_MOESM8_ESM.pdf]

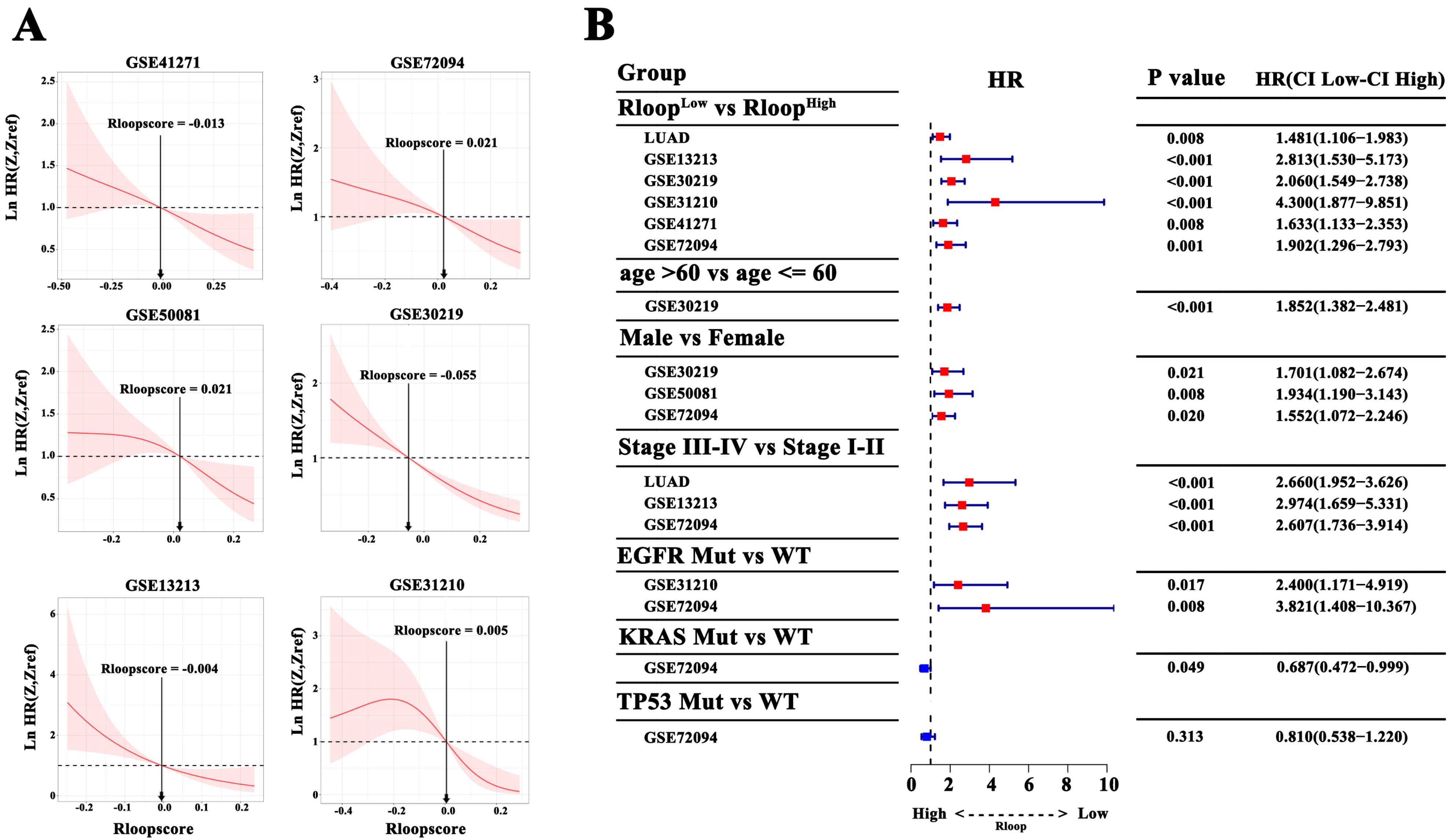
A
GSE41271
Rloopscore = -0.013
GSE72094
Rloopscore = 0.021
GSE50081
Rloopscore = 0.021
GSE30219
Rloopscore = -0.055
GSE13213
Rloopscore = -0.004
GSE31210
Rloopscore = 0.005
Ln HR(Z,Zref)
Rloopscore
B
Group
HR
P value
HR(CI Low-CI High)
Rloop Low vs Rloop High
LUAD 0.008 1.481(1.106−1.983)
GSE13213 <0.001 2.813(1.530−5.173)
GSE30219 <0.001 2.060(1.549−2.738)
GSE31210 <0.001 4.300(1.877−9.851)
GSE41271 0.008 1.633(1.133−2.353)
GSE72094 0.001 1.902(1.296−2.793)
age >60 vs age <= 60
GSE30219 <0.001 1.852(1.382−2.481)
Male vs Female
GSE30219 0.021 1.701(1.082−2.674)
GSE50081 0.008 1.934(1.190−3.143)
GSE72094 0.020 1.552(1.072−2.246)
Stage III-IV vs Stage I-II
LUAD <0.001 2.660(1.952−3.626)
GSE13213 <0.001 2.974(1.659−5.331)
GSE72094 <0.001 2.607(1.736−3.914)
EGFR Mut vs WT
GSE31210 0.017 2.400(1.171−4.919)
GSE72094 0.008 3.821(1.408−10.367)
KRAS Mut vs WT
GSE72094 0.049 0.687(0.472−0.999)
TP53 Mut vs WT
GSE72094 0.313 0.810(0.538−1.220)
High <- - - - - - - - - - > Low
Rloop
